# Large-scale identification of genes involved in septal pore plugging in multicellular fungi

**DOI:** 10.1038/s41467-023-36925-y

**Published:** 2023-03-17

**Authors:** Md. Abdulla Al Mamun, Wei Cao, Shugo Nakamura, Jun-ichi Maruyama

**Affiliations:** 1 Department of Biotechnology, The University of Tokyo, Tokyo, Japan; 2 Research Center for Agricultural Information Technology, National Agriculture and Food Research Organization (NARO), Tsukuba, Ibaraki Japan; 3 Department of Information Networking for Innovation and Design, Faculty of Information Networking for Innovation and Design, Toyo University, Tokyo, Japan; 4 Collaborative Research Institute for Innovative Microbiology, The University of Tokyo, Tokyo, Japan

**Keywords:** Cellular microbiology, Fungal genetics, Cell biology, Fungal physiology

## Abstract

Multicellular filamentous fungi have septal pores that allow cytoplasmic exchange, and thus connectivity, between neighboring cells in the filament. Hyphal wounding and other stress conditions induce septal pore closure to minimize cytoplasmic loss. However, the composition of the septal pore and the mechanisms underlying its function are not well understood. Here, we set out to identify new septal components by determining the subcellular localization of 776 uncharacterized proteins in a multicellular ascomycete, *Aspergillus oryzae*. The set of 776 uncharacterized proteins was selected on the basis that their genes were present in the genomes of multicellular, septal pore-bearing ascomycetes (three *Aspergillus* species, in subdivision Pezizomycotina) and absent/divergent in the genomes of septal pore-lacking ascomycetes (yeasts). Upon determining their subcellular localization, 62 proteins were found to localize to the septum or septal pore. Deletion of the encoding genes revealed that 23 proteins are involved in regulating septal pore plugging upon hyphal wounding. Thus, this study determines the subcellular localization of many uncharacterized proteins in *A. oryzae* and, in particular, identifies a set of proteins involved in septal pore function.

## Abstract (web summary)

Multicellular fungi have septal pores that allow cytoplasmic exchange between adjacent cells; cell wounding and other stress conditions induce septal pore closure. Here, Mamun et al. determine the subcellular localization of hundreds of uncharacterized proteins in a multicellular fungus, identifying 62 proteins associated with the septum. Of these, 23 proteins are involved in septal pore plugging upon hyphal wounding.

## Introduction

The emergence of multicellularity represents an important transition in evolutionary history. Organisms from diverse origins have organized into simple multicellular morphologies such as filaments, clusters, and sheets, where intercellular communication is limited owing to a lack of direct passageways in the subsequent cell population^1,2^. In relatively late evolutionary history, several groups of eukaryotic organisms have developed complex multicellularity through cell–cell adhesion and developmental programs to differentiate into tissues, reproductive organs, and fruiting bodies^1,3^. Further, complex multicellular organisms have evolved cell-to-cell connectivity via ultrastructural passageways to facilitate signal transmission^4–6^. Such cell-to-cell connectivity has evolved independently in animals, plants, and multicellular fungi; moreover, its regulation confers selective advantages in unfavorable environments. In animals, gap junctions, which allow ions and small molecules to pass, are perturbed structurally and functionally by oxidative stress^7^. In plants, plasmodesmata, which mediate cell-to-cell trafficking of transcription factors and signaling molecules, can be blocked by callose deposition in response to stresses^8^.

Fungi include both septal pore-bearing (filamentous fungi) and -lacking (yeast) species, facilitating the characterization of septal pore-related organization based on comparisons. Fungal hyphae grow by extending polarized tips and further compartmentalize with septa in relation to cell size and nuclear division^9^. At the center of the septum lies a septal pore that allows the exchange of cytoplasmic constituents between flanking cells^10,11^. In the subphylum Pezizomycotina of Ascomycota, these pores can be plugged by the peroxisome-derived, fungal-specific Woronin body^12^. In contrast, species of the subphylum Agaricomycotina of Basidiomycota have developed an endoplasmic reticulum-derived septal pore cap (SPC) to block the pores^13,14^. A mutant lacking the Woronin body matrix protein Hex1 exhibited extensive loss of cytoplasm from flanking cells via septal pores upon hyphal wounding^12^.

Septal pore function is dynamically regulated in response to mechanical, environmental, and physiological conditions. Stresses such as low temperature and low pH reduce cell-to-cell connectivity via unknown mechanisms^10,11^. Hyphal wounding and abiotic stresses induce the accumulation of septal pore-associated (SPA) proteins and the SO (or SOFT) protein at the pore^15–17^. Furthermore, the pore closes transiently during mitosis and opens during interphase when Nima kinase, a cell-cycle regulator, moves from the nucleus to the septal pore^18^. Septal pores are also modulated by physiological conditions such as hyphal age^11,19^, suggesting the involvement of additional components in regulating their functions. Considering these dynamic behaviors of septal pore regulation, our understanding of the underlying mechanisms is lacking, particularly given limited knowledge of Woronin body function.

Hyphae, the signature of fungal multicellularity, are proposed to have evolved early in fungal evolution, possibly in early diverging fungi such as Blastocladiomycota, Chytridiomycota, Zoopagomycota, and Mucoromycota^20^. Using large-scale genomic comparison, Kiss et al. proposed a set of genes involved in hyphal morphogenesis that are shared among fungal classes regardless of the presence or absence of filamentous hyphae^20^. Moreover, while fungal species within Pezizomycotina and Agaricomycotina typically show a substantial gain of gene families, as exemplified by morphological complexities such as septal pore, yeasts show a higher rate of gene losses^20,21^. Hyphal morphology is generally considered to be the foundation of fungal multicellularity, while the emergence and regulation of the septal pore have evolved independently in multicellular lineages, conferring further morphological complexities. However, these evolutionary steps in septal pore emergence and regulation remain poorly understood.

Here, we combined genomic comparisons between septal pore-bearing and -lacking ascomycetes with large-scale localization screening of candidate septal pore proteins to identify novel septal components. We found that the septal pore is a focus for localizing the proteins involved in its dynamic regulation. Using a bioinformatics approach including extensive phylogenetic analyses, we elucidated the patterns of gene evolution involved in septal pore plugging upon hyphal wounding.

## Results

### Selection of candidate septal pore proteins

**Fig. 1:**
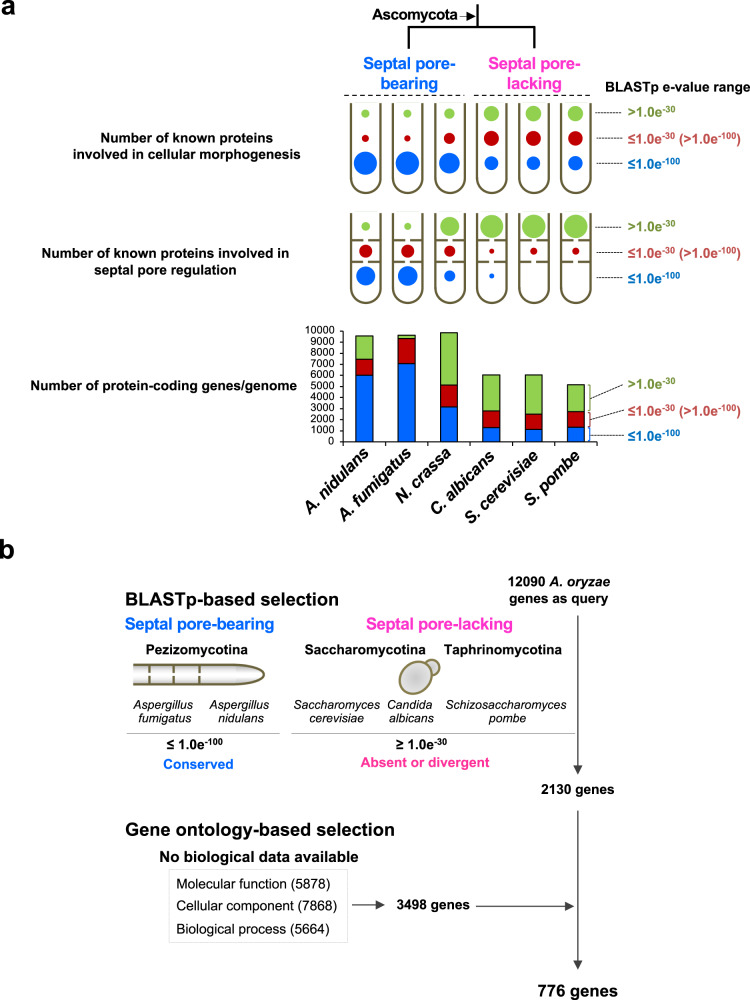
Strategy for selecting candidate septal pore proteins. **a** BLASTp-based genomic comparison among the septal pore-bearing and -lacking ascomycetes. Upper cartoons of hyphae show the hyphal morphology, and lower cartoons of hyphae with perforated septa indicate the septal pore and its regulation. The bubble size is proportional to the number of known proteins involved in cellular morphogenesis (upper bubble chart, Supplementary Data 1a) and septal pore regulation (lower bubble chart, Supplementary Data 1b). In the bar graph, the *Y* axis represents the number of total protein-coding genes per analyzed genome. Colors in bubbles and bars represent the proteins within the analyzed BLASTp *e* values; blue, red, and green indicate BLASTp *e* value ranges ≤1.0e-100, ≤1.0e-30 (>1.0e-100), and >1.0e-30, respectively. **b** Strategy for the selection of candidate septal pore proteins.

Here, *Aspergillus oryzae* was used owing to the availability of advanced experimental techniques for quantifying septal pore plugging upon hyphal wounding induced by hypotonic shock, as well as for quantifying cell-to-cell connectivity upon cold stress using photoconvertible fluorescent proteins^10,16,19,22,23^. The *A. oryzae* genome was compared with those of septal pore-bearing ascomycetes *A. nidulans*, *A. fumigatus*, and *Neurospora crassa* (Pezizomycotina) as well as septal pore-lacking ascomycetes *Saccharomyces cerevisiae*, *Candida albicans* (Saccharomycotina), and *Schizosaccharomyces pombe* (Taphrinomycotina) using BLASTp (Fig. 1a). First, the proteins known to function in cellular morphogenesis (encoded by 243 genes that have orthologs in *A. fumigatus*^20^) (Supplementary Data 1a) were compared. The majority of proteins from the septal pore-bearing species showed higher sequence homologies (Fig. 1a, blue bubble in upper bubble chart), and a considerable number of proteins also exhibited sequence homologies with proteins from the septal pore-lacking species (Fig. 1a, red and blue bubbles in upper bubble chart). This suggested that cellular morphogenesis-related genes evolved before their divergence. Similarly, we analyzed proteins known to function in septal pore regulation (Supplementary Data 1b based on previous reports^15–17,23,24^). Most of these proteins showed no or limited homologies with proteins from the septal pore-lacking species (Fig. 1a, green bubble in lower bubble chart), while many exhibited sequence homologies with the septal pore-bearing species (Fig. 1a, blue bubble in lower bubble chart). This result suggested that these proteins arose in the lineage leading to Pezizomycotina, but were lost or diverged in ascomycete yeasts. Next, to analyze the conservation of protein-coding genes more widely, we compared whole proteomic datasets from the aforementioned species with the proteome of *A. oryzae*. A large number of proteins with higher sequence homologies were found within the septal pore-bearing species, but a lesser number was found in the septal pore-lacking species (Fig. 1a, blue bar in bar graph). This conservation tendency is similar to that obtained in comparative analysis using the proteins involved in septal pore regulation (Fig. 1a, lower bubble chart). Based on these findings, we hypothesized that proteins involved in septal pore regulation might be conserved in septal pore-bearing species but absent or divergent in septal pore-lacking species.

To select candidate septal pore proteins, we screened for genes conserved in the septal pore-bearing ascomycetes *A. oryzae*, *A. fumigatus*, and *A. nidulans* (*e* values ≤1.0e-100 in BLASTp), but absent or divergent in the septal pore-lacking ascomycetes *S. cerevisiae*, *C. albicans*, and *S. pombe* (*e* values ≥1.0e-30 in BLASTp) (Fig. 1b). In total, 2130 genes were identified as potential candidates. These genes were further used for selecting uncharacterized candidates for which no biological data are available regarding any gene ontology (GO) terms; molecular function, cellular component, and biological process (Fig. 1b). Consequently, a subset of 776 uncharacterized candidate genes for septal pore proteins (Supplementary Data 2) were finally selected.

### Subcellular localization of candidate proteins

**Fig. 2:**
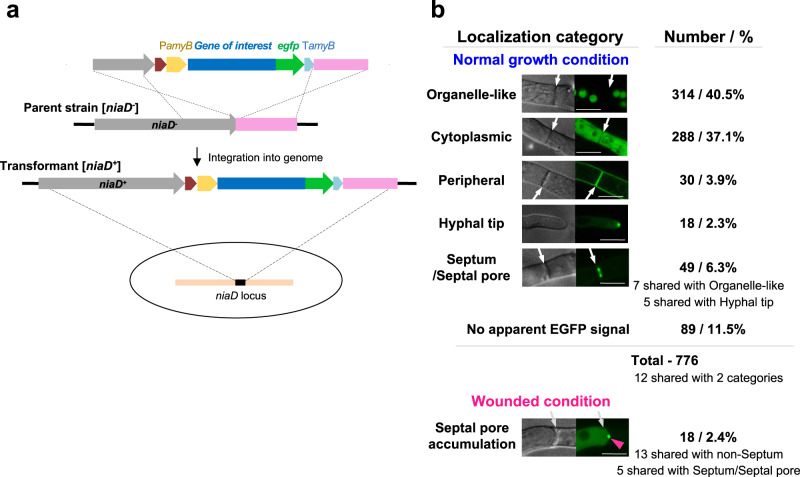
Subcellular localization of candidate septal pore proteins. **a** Schematic of the expression cassette for EGFP fusions of selected proteins to be inserted in the *A. oryzae* genome. **b** Subcellular localizations of the 776 proteins. Representative confocal fluorescence micrographs of localization categories are shown; arrows indicate septa. Scale bar, 5 μm.

Candidate gene sequences were retrieved from the Aspergillus genome database AspGD (currently closed, but the dataset is available in the FungiDB database; https://fungidb.org/fungidb/app). As reported in previous global analyses of protein localization in budding^25^ and fission^26^ yeasts, each of the 776 candidate genes was fused to *egfp* at its 3′ end under the control of the inducible *amyB* promoter^27^ (Fig. 2a). The expression cassettes were ectopically inserted into the *niaD* locus of *A. oryzae* strain NSlD1^10^ by homologous recombination (Fig. 2a and Supplementary Data 3: Strain list).

Of the 776 resulting strains, we detected EGFP fluorescence in the live hyphae of 687 (88.5%) strains (Fig. 2b). Their localization patterns were then classified into five categories: organelle-like, cytoplasmic, peripheral, hyphal tip, and septum/septal pore (Fig. 2b). In total, 314 (40.5%) proteins predominantly localizing as spherical structures, networks, and puncta resembling cell organelles were categorized as “organelle-like” localization (Fig. 2b and Supplementary Fig. 1). Diffuse, uniform cytoplasmic staining was observed for 288 (37.1%) proteins, while 30 (3.9%) exhibited peripheral localization (Fig. 2b and Supplementary Figs. 2 and 3a). However, many of the peripheral proteins overlapped with organelle-like distributions (Supplementary Fig. 3a), probably because of their presence in the secretory pathway. Nineteen of the peripherally localized proteins contained signal peptides and/or transmembrane domains (Supplementary Fig. 3a), supporting this hypothesis. Eighteen (2.3%) proteins localized to the hyphal tip (Fig. 2b and Supplementary Fig. 3b), whereas the remaining 89 proteins (listed in Supplementary Data 4) did not yield detectable EGFP signals.

**Fig. 3:**
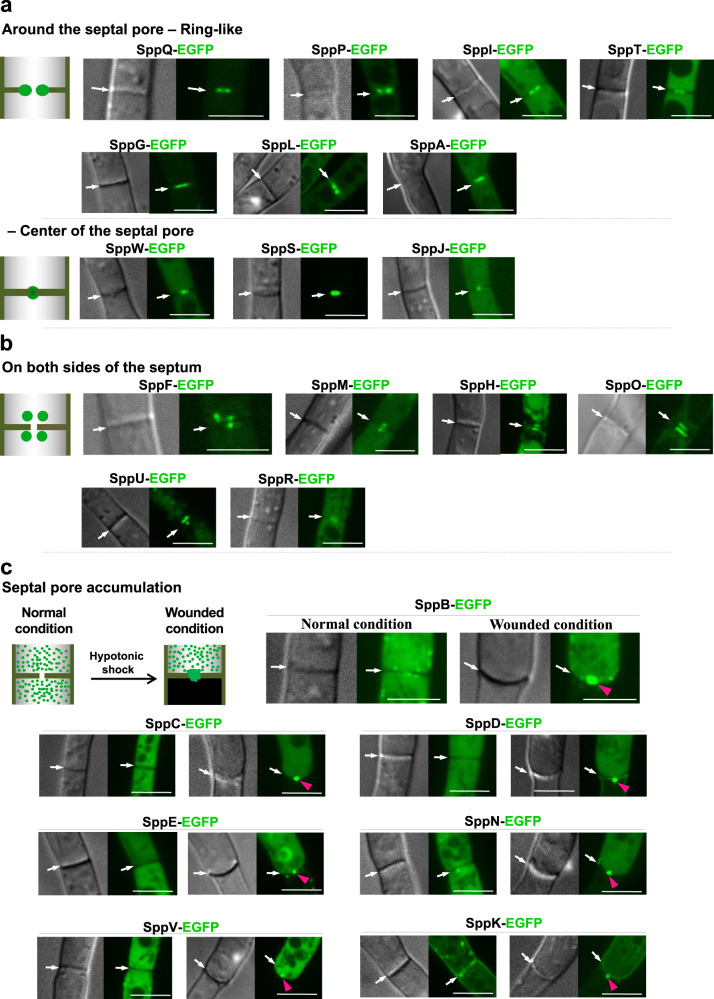
Localization of SPP proteins to the septum and septal pore. The apical septa of strains expressing EGFP-fused proteins were analyzed using confocal fluorescence microscopy under normal, control growth conditions and upon hyphal wounding. Cartoons represent the localization pattern categories observed at the septum. **a** Localization around the septal pore. **b** Localization on both sides of the septum. **c** Accumulation of non-septal SPP proteins at the septal pore upon hyphal wounding induced by hypotonic shock. White arrows indicate the septa, and red arrowheads indicate septal-pore accumulation. Scale bars, 5 μm.

Importantly, 62 proteins (8.0%) localized to the septum or septal pore (Fig. 2b). Forty-nine of these were septal under normal growth conditions (Fig. 3a, b and Supplementary Fig. 4a–c), and the remaining 13 accumulated at the septal pore upon hyphal wounding (Fig. 3c and Supplementary Fig. 4d). Interestingly, five septal proteins had overlapping distributions with those of the hyphal tip category (Supplementary Fig. 3b), suggesting shared regulatory networks between the septum and hyphal tip.

Collectively, our bioinformatics-based screening strategy allowed us to find many proteins showing localization related to the septum.

### The septal pore is a subcellular site to which many proteins localize

**Fig. 4:**
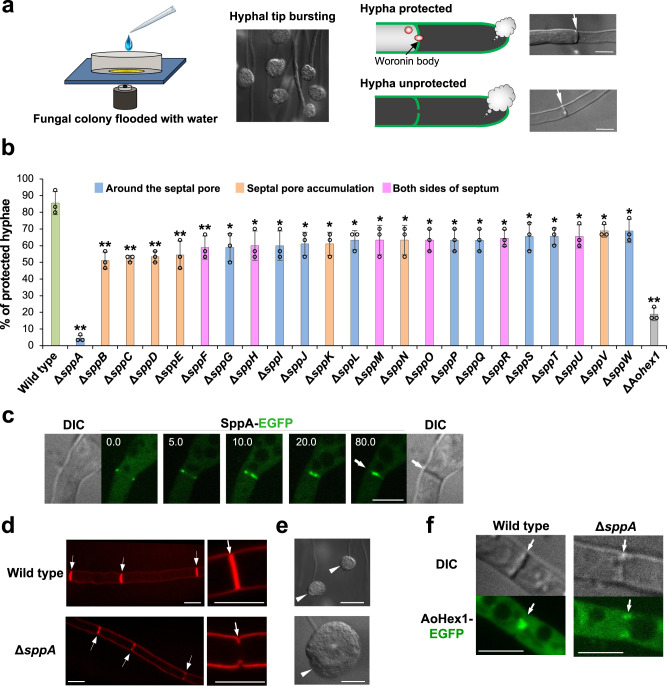
Regulation of septal pore plugging by SPP proteins upon wounding. **a** Schematic showing hyphal tip bursting induced by hypotonic shock. Cartoons and DIC images show wounded hyphae capable (top) or incapable (bottom) of protecting adjacent cells from cytoplasmic loss. Scale bars, 5 μm. **b** Protection of flanking cells from excessive loss of cytoplasm upon hyphal wounding. Thirty randomly selected hyphae showing hyphal tip bursting were observed in each experiment. Three independent experiments were performed, and the percentage of hyphae protected from the excessive loss of cytoplasm is shown on the *Y* axis. The data are presented as the mean of replicate experiments, and error bars represent standard deviations. Statistical significance was tested using two-tailed Student *t* test: *P < 0.05, **P < 0.01. Source data are provided as a Source Data file. **c** SppA localization at the site of septum formation. Strains were grown for 8 h in CD (2% glucose) liquid medium supplemented with 1% casamino acids. Time is indicated in minutes. White arrows indicate septa. Scale bars, 5 μm. **d** Incomplete septum formation in Δ*sppA* visualized using FM4-64. White arrows indicate septa. Scale bars, 5 μm. **e** Leaked cytoplasmic constituents in Δ*sppA* upon hyphal tip bursting induced by hypotonic shock. White arrowheads indicate leaked cytoplasmic constituents at the hyphal tip. Scale bars, 20 μm. **f** Abnormal tethering of Woronin bodies to the incomplete septum in Δ*sppA*. Single confocal fluorescence images at the apical septa are shown. White arrows indicate septa. Scale bars, 5 μm.

Of the 62 septal proteins, we later found that 23 were functionally involved in septal pore plugging upon hyphal wounding (see Fig. 4b) and designated them as septal pore plugging (SPP) proteins with an alphabetical order of functional importance in the septal pore-plugging activity (Fig. 4b).

Under normal growth, 49 proteins were classified into three categories based on their septal localizations: (1) around the septal pore (Fig. 3a and Supplementary Fig. 4a), (2) on both sides of the septum (Fig. 3b and Supplementary Fig. 4b), and (3) along the septum (Supplementary Fig. 4c).

The first category was further divided into two subgroups: ring-like localization, as two dots around the septal pore under confocal microscopy (26 proteins); and focal localization at the center of the septal pore (eight proteins) (Fig. 3a and Supplementary Fig. 4a).

Thirteen proteins were present on both sides of the septum near the septal pore, similar to the location of the Woronin body (Fig. 3b and Supplementary Fig. 4b). SppF and SppM showed punctate localization on both sides, parallel to the septal pore. However, the remaining proteins under this category localized broadly to both sides with multiple puncta (Fig. 3b and Supplementary Fig. 4b). SppF-EGFP colocalized with mCherry-tagged Woronin body matrix protein AoHex1 at the septum and in the cytoplasm (Supplementary Fig. 5a). However, the localization of the Woronin body to the septum was independent of SppF (Supplementary Fig. 5b). Two proteins were localized in the third category, along the septum, but not peripherally along the hyphae (Supplementary Fig. 4c).

The interconnected array of hyphal cells is vulnerable to mechanical injury, causing extensive loss of cytoplasm in the absence of septal pore plugging^10,12,16,22,23^. A number of cytoplasmic proteins accumulate at the septal pore upon wounding^10,15,16^. Therefore, all 776 EGFP-fusion-expressing strains were subjected to hypotonic shock-induced hyphal wounding^10,16,22,23^. Interestingly, 13 proteins showing cytoplasmic (five proteins), organelle-like (four proteins), and hyphal tip (four proteins) localizations under normal growth conditions were found to accumulate at the septal pore upon wounding (Fig. 3c and Supplementary Fig. 4d). Furthermore, SppI, SppT, and SppL, which localized around the septal pore, as well as SppO and SppF, which localized on both sides of the septum under normal growth, showed concentrated localization to the septal pore upon wounding (Supplementary Fig. 4e). The results highlighted these as candidate proteins with functions in response to wounding, as reported for the septal pore-accumulating proteins^15–17^.

### Role of SPP proteins in septal pore plugging

In animals, mechanical wounding induces dynamic remodeling of membrane function via mechanisms including fusion of secretory vesicles to the plasma membrane to promote healing^28^. Pezizomycotina employs the Woronin body to plug the septal pore upon wounding^12^. Here, septal pore-plugging activity was analyzed after the deletion of 62 candidate genes by replacing them with the *pyrG* selectable marker. Four deletions exhibited reduced colony growth (Supplementary Fig. 6a). The deletion strains were then subjected to hypotonic shock, which causes hyphal tip wounding (Fig. 4a)^10,16,22,23^. Hyphae protected from excessive cytoplasmic loss from the adjacent cells were counted using differential interference contrast microscopy (Fig. 4a).

In total, 23 (37%) deletion strains showed a significantly decreased ability to protect flanking cells from excessive cytoplasmic loss upon wounding compared with that in the wild-type control (Fig. 4b), indicating impaired septal pore plugging. Notably, deletion of *sppA* did not prevent cytoplasmic loss (Fig. 4b). This inability is possibly caused by a defect in septum formation, as SppA-EGFP transiently appeared at the site of septum formation, similar to contractile actin ring assembly and constriction (Fig. 4c). SppA, which possesses the C2 domain, is the ortholog of Inn1 and Fic1 in budding and fission yeasts, respectively (Supplementary Fig. 8a), both of which are essential for plasma membrane ingression during cytokinesis^29,30^. Because septum formation in Pezizomycotina is analogous to cytokinesis, we evaluated the septum morphology in Δ*sppA*. Wild-type controls exhibited disc-shaped walls compartmentalizing the hyphae (Fig. 4d). In contrast, Δ*sppA* showed abnormal septa with incomplete ingression of the plasma membrane from the cortex (Fig. 4d). A larger volume of cytoplasmic constituents from continuously interconnected cells leaked in Δ*sppA* upon hyphal wounding (Fig. 4e). In addition, Woronin bodies were abnormally tethered to the incomplete septum at the hyphal cortex in Δ*sppA* (Fig. 4f).

The absence of SppB, SppC, SppD, SppE, and SppF increased cytoplasmic loss via the septal pore relative to that in the wild-type strain (Fig. 4b), indicating the functional relevance of these proteins in septal pore plugging upon wounding. Expression of EGFP-fusion proteins attenuated the septal pore-plugging defect of the corresponding gene deletions (Supplementary Fig. 6b), confirming that the fusions are functional. We then visualized Woronin bodies in the gene deletion backgrounds and found that they remained tethered to the septum (Supplementary Fig. 5b), suggesting that their tethering occurs independently of the SPP proteins. However, more than half of the 62 analyzed proteins did not appear to play a significant role in septal pore plugging upon wounding.

To investigate functional relationships among SPP proteins, we generated double deletion strains. Representative genes *sppG*, *sppF*, and *sppE* were selected from localization categories; around the septal pore, on both sides of the septum, and septal pore accumulation, respectively. The selected representative genes were deleted in the deletion background of another gene under the same localization category. After hyphal wounding, none of the double deletions except for Δ*sppG*Δ*sppA* showed a further reduction in septal pore plugging compared with that in the single deletion (Supplementary Fig. 6c). Moreover, the single deletions revealed a measurable phenotype, whereas the corresponding double deletions did not display further deficiencies in septal pore plugging. These results suggested that each SPP protein played a non-overlapping role and could not be fully substituted by one another.

### SPP proteins and cell-to-cell connectivity under cold stress

**Fig. 5:**
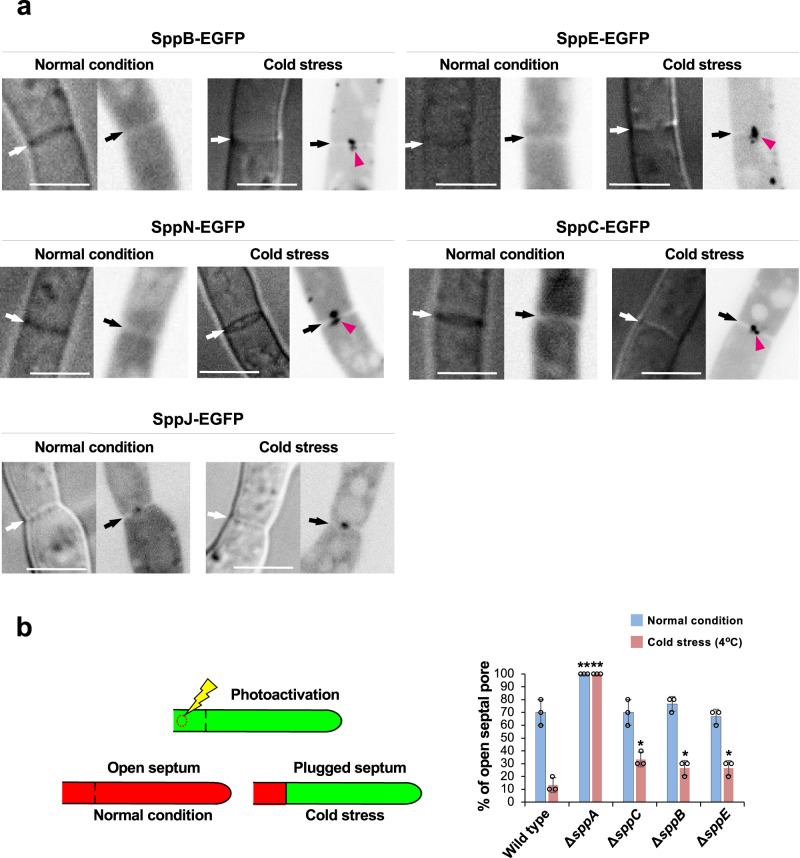
Regulation of cell-to-cell connectivity by SPP proteins under cold stress. **a** Localization of SPP-EGFP-fusion proteins. Black/white arrows indicate septa, and pink arrowheads indicate septal pore accumulation. Single confocal fluorescence images are shown. Scale bars, 5 μm. **b** Models for photoactivation and cell-to-cell transfer of Dendra2 (left); quantification of transfer (right). Scale bars, 10 μm. Dendra2 transfer through the septal pore was monitored at ten randomly chosen apical septa in each experiment. Three independent experiments were performed, and the percentage of open septal pore is shown on the *Y* axis. The data are presented as the mean of replicate experiments, and error bars represent standard deviations. Statistical significance was tested using two-tailed Student’s *t* test: *P < 0.05, **P < 0.01. Source data are provided as a Source Data file.

Because the septal pore closes under stress^10,11^, 62 strains expressing the septum-localizing proteins tagged with EGFP were subjected to cold stress (4 °C) to assess their dynamic behavior at the septal pore. Interestingly, four SPP proteins that normally localized to the cytoplasm were observed at the septal pore under cold stress (Fig. 5a). SppB, SppE, and SppN accumulated at the septal pore from both flanking cells, while SppC was present as puncta at the center of the septal pore (Fig. 5a). Localization of SppJ to the septal pore was promoted by cold stress (Fig. 5a).

The status of cell-to-cell connectivity in the gene deletion backgrounds lacking 62 septal proteins was monitored under cold stress by tracking the cell-to-cell transfer of Dendra2, a green-to-red photoconvertible fluorescent protein^10^ (Fig. 5b, left). In wild-type controls, the red fluorescence of photoconverted Dendra2 was transferred to adjacent cells via the septal pore, but this transfer was dramatically decreased at 4 °C (Fig. 5b, right). In contrast, the diffusion of Dendra2 was not at all restricted in Δ*sppA* under both normal and cold temperatures (Fig. 5b, right), indicating continuous cell-to-cell connectivity caused by incomplete septum formation (Fig. 4d). The Δ*sppB*, Δ*sppE*, and Δ*sppC* strains still allowed Dendra2 transfer at low temperature (Fig. 5b, right), indicating a partially aberrant regulation of septal pore function upon cold stress.

### Role of the disordered region of SPP proteins in septal pore accumulation

**Fig. 6:**
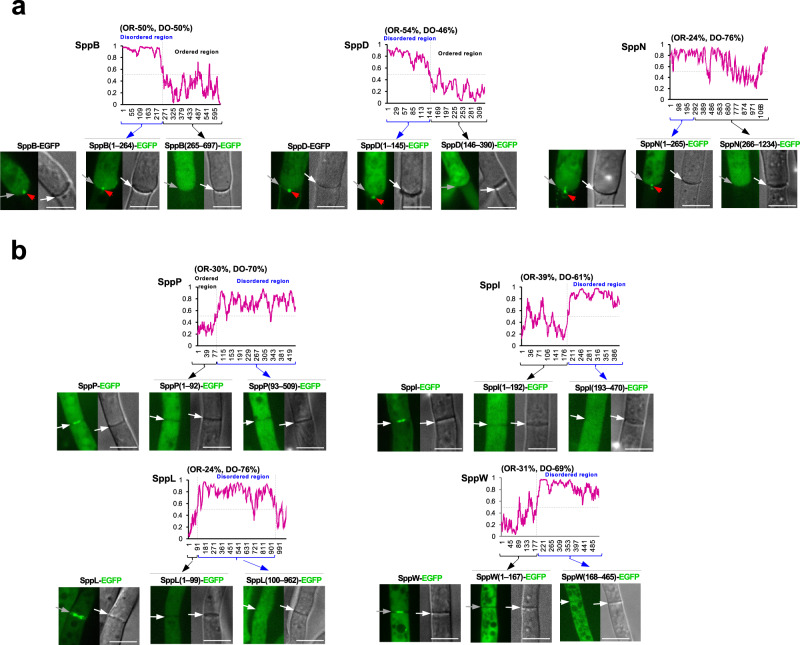
Prediction of disordered regions and analysis of truncated SPP protein variant localization. **a** Three SPP proteins showing septal pore accumulation upon hyphal wounding harbored a considerable length of a disordered region separated from the ordered region. **b** Four SPP proteins localized around the septal pore and harbored a considerable length of the disordered region separated from the ordered region. Graphs show the prediction of disordered regions using IUPred2A; the *Y* axis indicates the predicted probability of disorder, and the *X* axis represents the amino acid sequence. In the fluorescence microscopic analysis, full-length and truncated variants of EGFP-fused SPP proteins were expressed in the corresponding deletion background. Strains were grown in CD medium supplemented with 1% casamino acids for 18 h. Single confocal fluorescence images are shown. Arrows indicate apical septa, and red arrowheads indicate septal pore accumulation. Scale bars, 5 μm. OR ordered, DO disordered.

A large number of septal proteins including SPP proteins are associated with a tiny subcellular site, the septal pore. Next, we elucidated whether SPP proteins possess specialized sequences or structural features, which mechanically target them to the septum as reported with the disordered region of *N. crassa* SPA proteins^17^. First, we analyzed the degree and distribution of disordered regions present in the SPP proteins using IUPred2A^31^. Thirteen of the 23 SPP proteins possess sequences with a high probability of intrinsic disorder (Fig. 6 and Supplementary Fig. 7a, b). Typically, SPP proteins localizing on both sides of the septum were structurally ordered, while those localizing around or accumulating at the septal pore were mostly disordered (Fig. 6 and Supplementary Fig. 7b). To investigate the importance of the disordered region in septal localization, we expressed EGFP-tagged truncated variants. Interestingly, the disordered N-termini of both SppB and SppD were sufficient and essential for septal accumulation upon wounding (Fig. 6a). Disordered regions containing proline-rich domains are important for protein aggregation in phase separation behavior^32,33^. Beside SppB, the SppN sequence was prone to having disordered regions across its entire length and contained conserved proline-rich motifs in the N-terminal residues 1–265 (Supplementary Fig. 7c, d), which were sufficient and required for wound-induced septal accumulation (Fig. 6a). Contrastingly, in SppP, SppI, SppL, and SppW, both the ordered and disordered regions were essential for their normal septal localization (Fig. 6b). Collectively, the disordered region is important for wound-induced septal accumulation and normal septal localization.

Next, we analyzed the disordered regions in terms of amino acid composition and compared them with those of *N. crassa* SPA proteins, and disordered proteins/regions from the DisProt dataset, as well as the disordered proteins from phenylalanine/glycine (FG)-repeat nucleoporins (FG-Nups) and serine/arginine (SR)-repeat splicing factors. The disordered regions of both SPP and SPA proteins revealed biases to arginine and histidine, but showed antipathies to lysine and glycine when compared with FG nucleoporins, SR splicing factors, and the disordered proteins/regions from the Disprot dataset (Supplementary Fig. 7e).

### Orthologous relation of SPP proteins in fungi

**Fig. 7:**
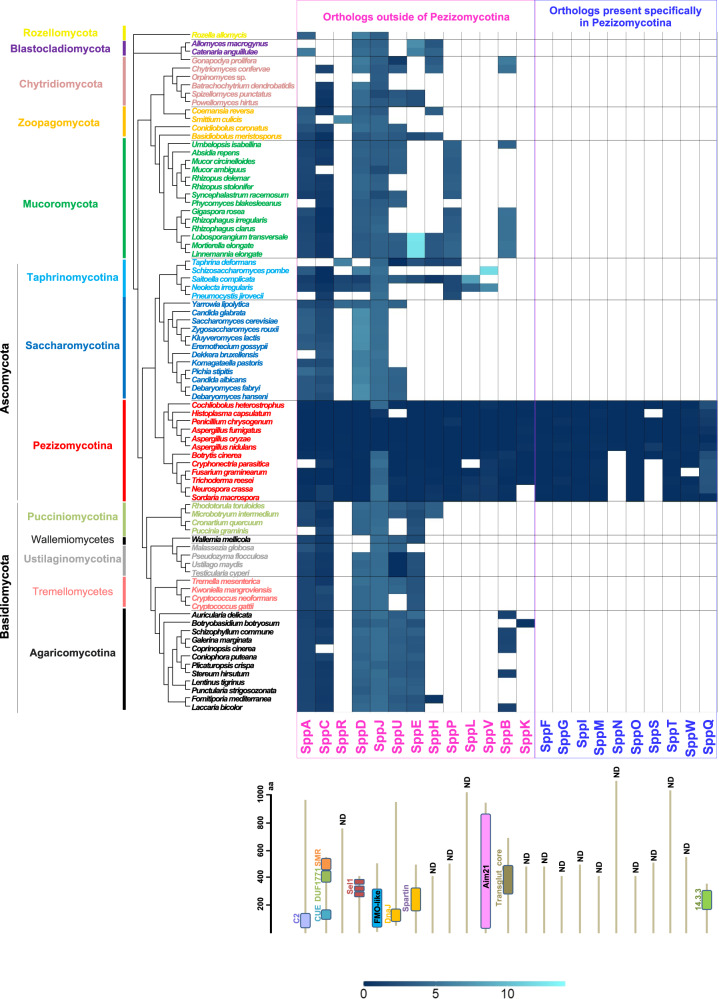
Orthologous relations and substitution rates of SPP proteins among fungal taxa. Amino-acid substitutions per site are shown in blue as a heat map. Filled and empty colors denote the presence and absence of orthologous proteins, respectively, according to the results of OrthoFinder. The phylogenetic tree was generated using the orthologous group proteins with a single ortholog present in almost all species. The scale on the left of the models of SPP protein structures represents the number of amino acids. ND no domain.

We analyzed the phylogenetic distribution and degree of divergence of SPP proteins compared with broad data of protein sequences retrieved from representative species covering all major fungal phyla and subphyla. Accordingly, we selected species from subphyla within Ascomycota (Taphrinomycotina, Saccharomycotina, and Pezizomycotina) and Basidiomycota (Agaricomycotina, Pucciniomycotina, and Ustilaginomycotina), as well as representatives of early diverging lineages, Mucoromycota, Zoopagomycota, Chytridiomycota, Blastocladiomycota, and Cryptomycota (Rozellomycota)^20,34^. Owing to its high rate of evolution^35^, Microsporidia were excluded from this analysis. Firstly, the proteome data of 81 fungal species were categorized into orthologous groups using OrthoFinder^36^. Subsequently, substitution rates of orthologous proteins in fungal phyla/subphyla from the Pezizomycotina root were calculated based on maximum likelihood phylogenetic trees (Fig. 7 and Supplementary Data 5a–c). To further verify orthologous relations, SPP protein phylogenies were generated for each orthologous group (Supplementary Figs. S8 and 9). Together with phylogenetic analyses, SPP proteins were finally classified into two evolutionary groups; (1) those with orthologs outside of Pezizomycotina, and (2) those present specifically in Pezizomycotina (Fig. 7).

Thirteen proteins (SppA, SppC, SppR, SppD, SppJ, SppU, SppE, SppH, SppP, SppL, SppV, SppB, and SppK) exhibited orthologs outside of Pezizomycotina. Among them, SppA, SppC, and SppR possessed orthologs in septal pore-lacking ascomycete yeasts, Saccharomycotina and Taphrinomycotina, in the same clade. SppA contains the C2 domain (PF00168)^37^ and belongs to a broad orthologous group that includes Inn1 and Fic1 from budding and fission yeasts, respectively (Fig. 7 and Supplementary Fig. 8a). SppC possesses an N-terminal CUE (PF02845)^38^, a C-terminal SMR (PF01713)^39^, and DUF1771 (SM001162)^40^ domains. Similar to SppA, SppC belongs to a broad orthologous group (Fig. 7 and Supplementary Fig. 8b). SppR was grouped with the peroxisomal membrane protein Pex8 from some fungal species (Fig. 7 and Supplementary Fig. 8c).

SppD, SppJ, and SppU exhibited multiple subclades, including Pezizomycotina, within their orthologous groups. SppD possesses three repeats of the SEL1 domain (SM00671)^41^ and forms a Pezizomycotina-specific subclade within the large clade including other fungal orthologous proteins (Fig. 7 and Supplementary Fig. 8d). SppJ contains a DnaJ domain (PF00226)^42^ and forms a Pezizomycotina-specific subclade close to some species from Chytridiomycota and Mucoromycota (Fig. 7 and Supplementary Fig. 8e). SppU contains an FMO-like domain (PF00743)^43^ and forms a subclade including Pezizomycotina and a limited number of Ustilaginomycotina, Taphrinomycotina, and Chytridiomycota species (Fig. 7 and Supplementary Fig. 8f).

Furthermore, SppE, SppH, SppP, SppL, and SppV proteins exhibited orthologous relations with other fungal phyla/subphyla, including the ascomycete yeasts Taphrinomycotina. SppE contains a plant-related senescence domain (PF06911)^44^ and belongs to an orthologous group, including Basidiomycota, early diverging fungi, and Taphrinomycotina (Fig. 7 and Supplementary Fig. 8g). SppH and SppP, which do not possess known domains, shared orthologous relations with proteins from early diverging fungi and some species of Taphrinomycotina (Fig. 7 and Supplementary Fig. 8h, i). SppL and SppV, with the latter harboring an Aim21 domain (PF11489)^45^, exhibited orthologous relations with a limited number of species from Taphrinomycotina (Fig. 7 and Supplementary Fig. 8j, k).

SppB and SppK also exhibited orthologs outside of Pezizomycotina, but not in any of the ascomycete yeasts. SppB, which contains a Transglut_core domain (PF01841)^46^, belonged to an orthologous group that includes some species from Chytridiomycota, Mucoromycota, and Agaricomycotina (Fig. 7 and Supplementary Fig. 8l). SppK showed an orthologous relation only with some species from Agaricomycotina (Fig. 7 and Supplementary Fig. 8m).

As the other group, a total of ten SPP proteins were identified as Pezizomycotina-specific existence. Nine SPP proteins, including SppF, SppG, SppI, SppM, SppN, SppO, SppS, SppT, and SppW, do not harbor any domains but lack orthologous relations with other fungal phyla and subphyla (Fig. 7 and Supplementary Fig. 9a-i). SppQ contains a 14-3-3 domain (PF00244)^47^ and belongs to an orthologous group including Pezizomycotina species only, which is distinct from another group containing the same domain (Fig. 7 and Supplementary Fig. 9j). These SPP proteins are conserved commonly within Pezizomycotina with the exceptions of SppN and SppS (Fig. 7).

### Functional dissection of SppA and SppC

Despite our bioinformatics-based screen to select genes absent or divergent in ascomycete yeasts (Fig. 1b), two SPP proteins, SppA and SppC, contain respective yeast orthologs within the same clades (Supplementary Fig. 8a, b). Therefore, these proteins were functionally analyzed based on their structural differences.

**Fig. 8:**
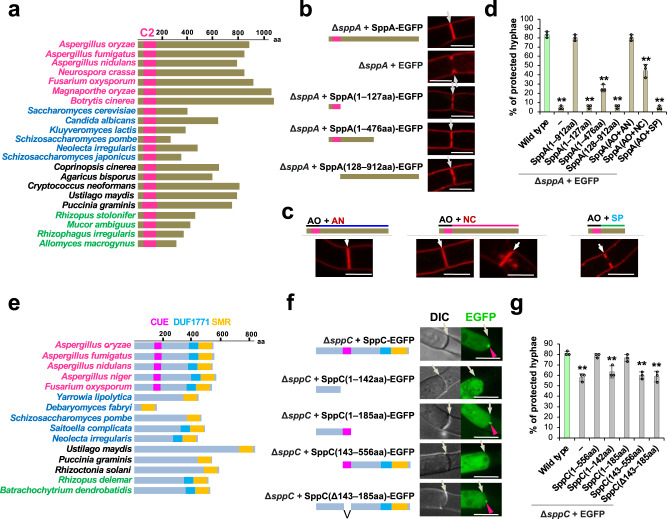
Functional dissection of SppA and SppC. **a, e** Lengths of SppA and SppC with their fungal orthologs. Pink, blue, black, and green texts indicate species in Pezizomycotina, ascomycete yeasts, Basidiomycetes, and early diverging fungi, respectively. Bars represent known protein domains. The numbers of amino acid residues are shown in the scale at the top. **b** Both N- and extended C-terminal regions of SppA are essential for normal septum formation. Apical septa were visualized using FM4-64 dye. **c** Septa in strains expressing SppA chimeric proteins with the N-terminus along with the C2 domain derived from *A. oryzae* and the C-terminus derived from the septal pore-bearing or -lacking ascomycetes. AO *Aspergillus oryzae*, AN *Aspergillus nidulans*, NC *Neurospora crassa,* SP *Schizosaccharomyces pombe*. Septa were visualized using FM4-64 dye. SppA(AO + NC) rescued the defective phenotypes to a notable extent, and the two typical images of normal and abnormal septum formation are shown. Scale bars, 5 μm. **d**, **g** Protection of flanking cells from cytoplasmic loss upon hyphal wounding. Thirty randomly selected hyphae showing hyphal tip bursting were observed in each experiment. Three independent experiments were performed, and the percentage of hyphae protected from the excessive loss of cytoplasm is shown on the *Y* axis. The data are presented as the mean of replicate experiments, and error bars represent standard deviations. Statistical significance was tested using two-tailed Student’s *t* test: **P < 0.01. Source data are provided as a Source Data file. **f** The N-terminus of SppC is essential for accumulation at the septal pore upon hyphal wounding. Single confocal fluorescence images are shown. White arrows indicate septa, and red arrowheads represent septal pore localization. Scale bars, 5 μm.

SppA possesses an N-terminal C2 domain, which is conserved in the fungal orthologous proteins. However, the length of the C-terminus varies among the orthologous proteins and is longer in Pezizomycotina species than in ascomycete yeasts and other fungi (Fig. 8a). To elucidate the functional importance of individual regions, truncated variants of SppA were expressed as EGFP fusions in the Δ*sppA* background. Their expression levels were similar to that of the full-length SppA (Supplementary Fig. 10a, left). An SppA variant (residues 128–912) lacking the N-terminal region along with C2 domain could neither complete septum formation nor prevent excessive cytoplasmic loss upon hyphal wounding (Fig. 8b, d). In addition, this variant did not localize to the septum (Supplementary Fig. 10b), consistent with the known function of membrane targeting by the C2 domain^37^. Similarly, SppA truncations lacking the C-terminus (residues 1–127 and 1–476) failed to attenuate the septum pore-defective phenotype caused by s*ppA* deletion (Fig. 8b, d), indicating the requirement of these regions for proper septum formation and septal pore plugging. Next, we fused the N-terminal region of SppA along with the C2 domain of *A. oryzae* and the C-terminal region of septal pore-bearing *A. nidulans* and *N. crassa*, as well as of septal pore-lacking *S. pombe* (Fig. 8c). EGFP fusions of these chimeric constructs were expressed similarly to the full-length SppA (Supplementary Fig. 10a, left). Chimeric proteins containing the C-termini from *A. nidulans* [SppA(AO + AN)] and *N. crassa* [SppA(AO + NC)] rescued the septal pore-defective phenotypes completely and partially, respectively (Fig. 8c, d). In contrast, the construct with the C-terminus from the *S. pombe* ortholog Fic1 [SppA(AO + SP)] exhibited a defect similar to that of Δ*sppA* (Fig. 8c, d), suggesting that the yeast protein is not completely functional ortholog of SppA. Therefore, we concluded that the extended C-terminal region of SppA provided septum-related function(s), and the region has diversified in the lineage leading to Pezizomycotina but was shortened in ascomycete yeasts.

SppC possesses an N-terminal CUE domain, which shows diversity of domain presence or absence among its orthologs (Fig. 8e and Supplementary Fig. 8b). Multiple sequence alignment revealed diversity in MFP motifs but conservation of the LL motif (Supplementary Fig. 11), both of which are required for high-affinity binding with ubiquitin^48,49^. To identify the regions responsible for the septal pore-targeted accumulation, truncations of SppC were expressed as EGFP fusions in the Δ*sppC* background. Their expression levels were similar to that of the full-length SppC (Supplementary Fig. 10a, right). The N-terminal 185 residues containing the CUE domain were sufficient for accumulation at the septal pore upon wounding, similar to the full-length protein, whereas truncation of the N-terminal 142 residues prevented this response (Fig. 8f). However, only the N-terminal 142 residues without the CUE domain did not clearly accumulate at the septal pore (Fig. 8f), indicating that the N-terminal 142 residues are essential but not sufficient to accumulate at the septal pore. A longer N-terminal construct (residues 1–185) containing the CUE domain fully rescued the septal pore-defective phenotype of the Δ*sppC* (Fig. 8g). Two other SppC truncated variants (residues 143–556 and CUE domain-lacking Δ143–185) did not rescue the defective phenotype (Fig. 8g). Collectively, these results indicated that a complete N-terminus including the CUE domain was essential for the septal pore-related function of SppC.

Taken together, these results provide evidence that Pezizomycotina-specific features, such as the extended C-terminus of SppA and the N-terminal region of SppC, are important for septal function.

## Discussion

In this study, we compared two morphologically diverse fungal groups to better understand the genetic innovations leading to the evolution of the fungal septal pore and its regulation. Localization screening identified numerous functionally important septal proteins that were either absent or divergent in the septal pore-lacking yeasts. Our findings show that the Pezizomycotina has evolved at least 23 SPP proteins, which are classified either as those with orthologs outside of Pezizomycotina, or as those present specifically in Pezizomycotina, for septal pore regulation against adverse conditions such as wounding and cold stress.

Though large-scale localization screenings have previously been used as a powerful reverse-genetic approach in animals^50^, yeasts^25,26^, and bacteria^51^, we employed this strategy in filamentous fungi for the first time in the present study. Proteins localizing at the hyphal tip were found less frequently than those localizing at the septum (Fig. 2b and Supplementary Fig. 3b). This could be because some ascomycete yeasts, including the species used for our bioinformatics-based screening (Fig. 1b), can undergo pseudohyphal or hyphal growth^52^. Therefore, Pezizomycotina has not evolved many proteins that function specifically in hyphal tip growth. Five of the hyphal tip proteins also localized to the septum/septal pore (Fig. 2b and Supplementary Fig. 3b), similar to several known septal proteins^10,24^. Of these, three deletion strains lacking such proteins showed reduced colony growth compared to the wild-type strain (Supplementary Fig. 6a; except for SppA), suggesting their involvement in hyphal morphogenesis. The formation of perforated septa is evolutionarily specific to and emerged uniquely in multicellular fungi. Over half of our identified septal proteins (34/62) exhibited localization around the septal pore (Fig. 3a and Supplementary Fig. 4a), possibly contributing to septal-pore structure maintenance.

Filamentous fungi have evolved wound-management systems with multicellularity. The septate subphyla Pezizomycotina and Agaricomycotina have the Woronin body and the septal pore cap, respectively, to limit cytoplasmic leakage upon wounding^12–14^. In contrast, the aseptate phylum Mucoromycota exhibits protoplasmic gelation^53^. In this study, quantitative analysis of protected hyphae upon wounding revealed that more than a third (23/62) of the candidate septal proteins were involved in septal pore plugging (Fig. 4b). Double deletions of the *spp* genes did not cause further defects in the septal pore plugging (Supplementary Fig. 6c), which did not reach the severe defect level in the deletion of *Aohex1* encoding a Woronin body matrix protein (Fig. 4b). Woronin bodies, in which Hex1 protein forms the crystal dense core larger than the septal pore to withstand wound-induced turgor pressure^54^, play a primary role in the pore plugging function. However, Woronin body-mediated plugging may require plasma membrane resealing to complete the wound healing process for subsequent growth resumption. A portion of SPP proteins, which accumulated at the septal pore (Fig. 6a), exhibit disordered features with amino acid compositions common to those of *N. crassa* SPA proteins^17^ (Supplementary Fig. 7e). Disordered regions of the SPP proteins were important in septal accumulation upon wounding (Fig. 6a), and aggregation characteristics of disordered regions of the *N. crassa* SPA proteins suggest a function to consolidate Woronin bodies with septal pore^17^. Thus, these aspects together propose that SPP proteins along with *N. crassa* SPAs play a secondary role in septal pore plugging.

Further characterization of the functional domains present in several SPP proteins will help address the convergent evolution of regulatory components/mechanisms for cell-to-cell connectivity, including gap junctions, plasmodesmata, and septal pores. SppA possesses the C2 domain, which has been implicated in anchoring to plasmodesmata^55^. The SppC CUE domain possesses conserved ubiquitin-binding MFP and LL motifs^48,49^ (Supplementary Fig. 11). The gap-junction protein Cx43 undergoes ubiquitin-mediated degradation upon abiotic stress^56^, suggesting that ubiquitination could be the shared biochemical process regulating cell-to-cell connectivity in fungi and animals.

Cell-to-cell connectivity via gap junctions^56^, plasmodesmata^8^, and septal pores^10^ can be blocked in response to both biotic and abiotic stresses. In this study, the status of cell-to-cell connectivity was quantitively analyzed in response to cold stress (Fig. 5b). A strain lacking SppA showed an unrestricted cell-to-cell transfer of Dendra2 even under cold stress due to defective septum morphology (Fig. 5b). Three SPP proteins (SppB, SppC, and SppE) had a function in septal pore plugging under both wounding and cold stress (Figs. 4b and 5b), suggesting a common stress-response machinery. In general, SPP proteins responded less to cold stress than to hyphal wounding (Figs. 4b and 5b), suggesting their predominant involvement in protecting flanking cells from wound-induced cytoplasmic loss.

According to the orthologous group and phylogenetic analyses, 13 SPP proteins have orthologs outside of Pezizomycotina (Fig. 7). Particularly, in the SPP proteins with orthologs from early diverging fungi, their origins predate the emergence of the septal pore, suggesting co-option of the preexisting genes for septal pore regulation. Our bioinformatics-based screening was originally designed to identify genes that were divergent or absent in the septal pore-lacking yeasts (Fig. 1b). Contrary to our aim, 12 SPP proteins showed orthologous relations with those of the septal pore-lacking species from ascomycete yeasts and early diverging fungi (Fig. 7 and Supplementary Fig. 8). This finding raises a fundamental question regarding the principal and general functions of these proteins. SppA exhibits an extension at the C-terminus in Pezizomycotina (Fig. 8a), which could be evolutionarily specific to the porous septum formation. Yeast cytokinesis is morphologically different from that of Pezizomycotina, as yeast daughter cells are completely separated from the mother by SppA orthologs^29,30^. In this scenario, the inability of the *sppA* deletion mutant to plug the septal pore can be explained by defective cytokinesis or septum formation. Nif1 and Dsf2 are orthologs of SppD in fission and budding yeasts, respectively (Supplementary Fig. 8d). Nif1 acts as a mitotic inhibitor via interaction with Nim1 protein kinase^57^, whereas Dsf2 localizes to the bud neck^58^, the site of cytokinesis. This suggests a possible role of SppD in cell-cycle regulation or cytokinesis. The SppD subclade is distinct from another subclade containing Pezizomycotina and yeast orthologs (Supplementary Fig. 8d). Therefore, we hypothesize that SppD evolved in Pezizomycotina by gene duplication for performing septal pore-related functions. Thus, the functional role of SppD in porous septum formation requires further investigation.

SppC possesses a functionally important N-terminal region containing the CUE domain, which shows diversity of domain presence or absence among its orthologs (Fig. 8e and Supplementary Fig. 8b). The Cue2 protein, SppC ortholog in budding yeast, plays a role in ubiquitin binding^49^. SppJ containing the DnaJ domain exhibits an orthologous relation with budding yeast Sis1 (Supplementary Fig. 8e), which is involved in degrading cytosolic misfolded proteins^59^. However, the involvement of ubiquitination and misfolded protein responses in septal pore regulation remains to be investigated. In contrast, SppB does not possess any orthologs in ascomycete yeasts (Fig. 7). The transglutaminase domain of SppB is known for crosslinking properties, which function in mammalian wound healing and blood clotting upon injury^46^. This function is similar to wound-induced plugging of the fungal septal pore.

In the fungal kingdom, hyphal morphology is supposed to have evolved in the early diverging fungal phyla Blastocladiomycota, Chytridiomycota, and Zoopagomycota^20^. Septal pore and its regulation appear to have emerged independently in the subphyla Pezizomycotina and Agaricomycotina^1^. Therefore, during the evolutionary transition from aseptate ancestors, many gene evolutionary events were needed to modulate septal　pore organization and regulation. Our orthologous group and phylogenetic analyses (Fig. 7 and Supplementary Fig. 9) demonstrated that ten SPP genes are present specifically in Pezizomycotina. Similarly, specific existence within Pezizomycotina has been reported for the Woronin body-related genes for Hex1, its sorting receptor WSC, the Woronin body tether Leashin, and several of SPA proteins^21^. Such lineage-specific genes have been proposed to have arisen either from ancestrally non-genic regions or by excessive divergence following genomic rearrangement such as recombination, retrotransposition, and horizontal gene transfer^60,61^. The Pezizomycotina-specific existence of the ten SPP (Fig. 7 and Supplementary Fig. 9) and Woronin body-related genes^21^ suggests their emergence in a common ancestor of Pezizomycotina and subsequent acquisition by vertical transfer for aiding septal pore-related functions. This phenomenon may be similar to that underlying multiple lineage-specific genes present in yeast and worms that are functionally associated with chromosomal segregation^62^.

Our findings demonstrate the effectiveness of bioinformatics-based selection to identify many novel proteins involved in fungal septal pore plugging upon hyphal wounding. During the evolution of the septal structure, the interconnected cellular organization might have been vulnerable to environmental threats and physiological stresses such as mechanical wounding^10^, uninterrupted spreading of mycoviruses^63^, and allorecognition-mediated heterokaryon incompatibility^64^. Such adverse conditions could impose selection pressure for adaptation either by the co-option of preexisting genes or by the acquisition of new genes for aiding in septal pore regulation.

## Methods

### Selection of candidate septal pore proteins

Whole proteomes of seven fungal species were retrieved from the genome database FungiDB (https://www.fungidb.org). We used 12090 *A. oryzae* proteins and queried each using BLASTp. The presence or absence of the septal pore, as a specialized morphological structure, was set as the criterion for genomic comparison. We searched for conserved genes in the septal pore-bearing ascomycetes *A. oryzae*, *A. fumigatus*, and *A. nidulans* with e values less than or equal to 1e-100; we then searched for genes absent or diverged in the ascomycete yeasts *S. cerevisiae*, *S. pombe*, and *C. albicans*, with e values greater than or equal to 1e-30. This process yielded 2130 candidate septal pore proteins in *A. oryzae*.

We preferentially selected uncharacterized proteins, defined as those lacking the biological data for GO terms regarding molecular functions, cellular components, and biological processes. In total, 5878, 7868, and 5664 genes were identified for molecular functions, cellular components, and biological processes, respectively. A total of 3498 genes lacking biological data for all three GO terms were finally determined as uncharacterized genes. We then searched for the aforementioned 2130 candidate proteins within this uncharacterized subset, and shortlisted 776 genes for localization screening.

### Strains, growth conditions, and transformation

The strains used in this study are listed in Supplementary Data 3. *A. oryzae* was transformed as previously described^65^. *A. oryzae* strain NSPlD1 (*niaD*^−^ *sC*^−^ Δ*pyrG* Δ*ligD*)^10^ was used as the parent strain. Using the *pyrG* selectable marker, transformants were selected using M + Met medium [0.2% NH_4_Cl, 0.1% (NH_4_)_2_SO_4_, 0.05% KCl, 0.05% NaCl, 0.1% KH_2_PO_4_, 0.05% MgSO_4_·7H_2_O, 0.002% FeSO_4_·7H_2_O, 0.15% methionine, and 2% glucose; pH 5.5]. Transformants with the *niaD* and *sC* selectable markers were selected using CD medium (0.3% NaNO_3_, 0.2% KCl, 0.1% KH_2_PO_4_, 0.05% MgSO_4_·7H_2_O, 0.002% FeSO_4_·7H_2_O, and 2% glucose; pH 5.5) and CD + Met medium supplemented with 0.0015% methionine, respectively. *A. oryzae* strains were maintained in Potato Dextrose (PD) medium (Nissui, Tokyo, Japan) at 30 °C. For transformation, strains were inoculated into DPY liquid medium (0.5% yeast extract, 1% Hipolypeptone, 2% dextrin, 0.5% KH_2_PO_4_, 0.05% MgSO_4_·7H_2_O, pH 5.5) and grown overnight. For microscopy, conidia were inoculated on glass base dishes containing 100 μL CD liquid medium supplemented with 1% casamino acids. For hyphal wounding, agar medium was used instead of liquid medium. For complete induction and repression of the *amyB* promoter, 2% dextrin and 2% glycerol, respectively, were included in CD medium as carbon sources.

### DNA techniques

Nucleic-acid sequences for the 776 candidate septal pore proteins were retrieved from the Aspergillus genome database AspGD (currently closed, but the dataset is available in the FungiDB database; https://fungidb.org/fungidb/app). The open-reading frame encoding each protein was amplified from RIB40 genomic DNA with PrimeSTAR® HS DNA Polymerase (TaKaRa Bio, Otsu, Japan) for high-fidelity PCR, using the primers listed in Supplementary Data 6. The amplified fragments were then fused with *Sma*I-linearized pUt-C-EGFP^10^ using the In-Fusion HD Cloning Kit (Clontech Laboratories, Mountain View, CA, USA). Gene sequences fused with the *egfp* gene were positioned in tandem between the linker sequences. Recombinant plasmids used for the expression of EGFP-fused proteins were digested using *Not*I and inserted into the *niaD* locus of the wild-type strain NSlD1^10^ by homologous recombination. Transformants were selected based on nitrate assimilation. EGFP-fusion-expressing strains generated for localization screening are listed in Supplementary Data 3.

Primers used for generating the deletion strains are listed in Supplementary Data 6. Individual genes with the *pyrG* marker were replaced by amplifying the 1.5-kbp regions upstream and downstream of the genes using the primer sets gene-upstream1_F/gene-upstream2_R and gene-downstream1_F/gene-downstream2_R, respectively. The *pyrG* marker was amplified from RIB40 genomic DNA using the primers PyrG_F and PyrG_R. The three amplified DNA fragments (upstream and downstream regions of targeted genes and *pyrG*) and linearized-pUC19 vector were fused using the In-Fusion HD Cloning Kit. Deletion constructs were further amplified using the primer set gene-upstream1_F/gene-downstream2_R from the resultant plasmid as a template, and then introduced into the native locus of the *A. oryzae* strain NSPlD1^10^ by homologous recombination. Transformants were selected using uridine/uracil prototrophy.

Deletions of *sppG*, *sppF*, and *sppE* with the *sC* marker were performed by amplifying 1.5-kbp upstream and downstream regions using the primer sets gene_upstream1_F/gene-upstream2 (sC)_R and gene-downstream1(sC)_F/gene-downstream2_R, respectively. The *sC* marker was amplified from RIB40 genomic DNA using the primers sC_F and sC_R. The three fragments and linearized-pUC19 vector were fused. PCR-amplified deletion constructs of *sppG*, *sppF*, and *sppE* were introduced into the native loci of Δ*sppA*/Δ*sppP*/Δ*sppI*/Δ*sppT*/Δ*sppW*/Δ*sppL*/Δ*sppS*/Δ*sppJ*/Δ*sppQ*, Δ*sppM*/Δ*sppH*/Δ*sppO*/Δ*sppU*/Δ*sppR*, and Δ*sppB*/Δ*sppC*/Δ*sppD*/Δ*sppK*/Δ*sppN*/Δ*sppV*, respectively.

To analyze cell-to-cell connectivity via the septal pore under cold stress (Fig. 5), Dendra2, a green-to-red photoconvertible fluorescent protein, was used^10^. The Dendra2-expressing plasmid pUt-NA-dendra2^10^ was amplified and digested using *Not*I. Ethanol-precipitated DNA was inserted into the *niaD* locus of deletion strains lacking 62 septum-localizing proteins by homologous recombination.

To determine the importance of the disordered region, fragments encoding residues 1–264 and 265–697 of SppB; 1–145 and 146–390 of SppD; 1–265 and 265–1234 of SppN; 1–92, 93–509, 1–192, and 193–470 of SppI; 1–99 and 100–962 of SppL; and 1–167 and 168–465 of SppW were amplified. For truncated variants lacking the N-terminal region, a start codon was added. The individual fragment was then fused with *Sma*I-linearized pUt-C-EGFP. Recombinant plasmids were digested with *Not*I and inserted into the *niaD* locus of the corresponding deletion backgrounds by homologous recombination.

The primers used to truncate SppA and SppC are listed in Supplementary Data 6. The amplified truncated fragments were fused with *Sma*I-digested pUt-C-EGFP, and then a *Not*I-digested expression cassette was inserted into the *niaD* locus. To generate chimeric proteins of SppA (Fig. 7), a DNA encoding the N-terminus (residues 1–127) was amplified from RIB40 genomic DNA, and those for the C-termini of *A. nidulans* (residues 126–810), *N. crassa* (residues 127–863), and *S. pombe* (residues 135–810) were amplified using the genomic DNA of corresponding strains FGSC_A26, OR74A, and 972 h, respectively. The fragments were fused with *Sma*I-digested pUt-C-EGFP^10^ using the In-Fusion HD Cloning Kit. The constructed plasmids were digested using *Not*I and inserted into the *niaD* locus of Δ*sppA* by homologous recombination.

SppC was truncated from both the C- and N-termini. DNA fragments encoding the N-terminal residues 1–142 and 1–185 were amplified from RIB40 genomic DNA. They were fused with *Sma*I-digested pUt-C-EGFP. To truncate N-terminal regions containing the CUE domain, a DNA fragment encoding residues 143–556 was amplified by adding an ATG start codon. The fragment and *Sma*I-digested pUt-C-EGFP were fused using the In-Fusion HD Cloning Kit. The constructed plasmids were digested using *Not*I and used to insert expression cassettes into the *niaD* locus of Δ*sppC* by homologous recombination.

### Microscopy

Apical septa of living hyphae expressing EGFP-tagged proteins were observed using an IX71 inverted microscope (Olympus, Tokyo, Japan) equipped with 100× Neofluar objective lenses (1.40 numerical aperture); 488- (Furukawa Electric, Tokyo, Japan) and 561-nm (Melles Griot, Rochester, NY, USA) semiconductor lasers; GFP filters (Nippon Roper, Tokyo, Japan); a CSU22 confocal scanning system (Yokogawa Electronics, Tokyo, Japan); and an Andor iXon cooled digital CCD camera (Andor Technology PLC, Belfast, UK). Images were analyzed using Andor iQ 1.9 software (Andor Technology PLC).

### Hypotonic shock and Dendra2 transfer analyses

Hyphal tips were burst by flooding colonies with 1 mL of water after growth on a thin layer of DPY agar medium in a glass base dish at 30 °C for 18 h. After flooding, the colony was left for 2 min before observation. Thirty randomly selected hyphae showing burst tips were observed using differential interference contrast microscopy.

To analyze Dendra2 transfer, hyphae were grown on a thin layer of CD (2% dextrin as carbon source) medium supplemented with 1% casamino acids at 30 °C for 17 h. The culture was further incubated in normal or cold stress (4 °C, 30 min) conditions. Before observation, hyphae were illuminated with a UV mercury lamp positioned at least 20 μm from the selected apical septum to avoid illumination of the adjacent cell, as previously described^10^. Dendra2 transfer via the septal pore was observed after photoconversion with 553-nm excitation^10^.

### Disorder prediction and analysis of the amino acid composition

Disordered regions of SPP proteins were annotated using IUPred2A^31^. For amino acid composition analysis, the sequences of *N. crassa* SPA proteins were retrieved from the FungiDB genome database. As the disordered proteins/regions, IUPred2A-defined disordered regions of SPP and SPA proteins, disordered proteins of serine/arginine-rich (SR) splicing factors^66^ and phenylalanine/glycine repeats (FG) nucleoporins^67^, and disordered proteins/regions from the DisProt database^68^ were used. The ordered proteins/regions were taken from the O_PDB_S25 dataset^69^. Amino acid compositions of different disordered proteins/regions set were compared for each amino acid according to the previously developed method^17,70^. The fractional difference was calculated as (*P*_*X*_ − *P*_*order*_)/*P*_*order*_, where *P*_*X*_ represents the percentage of a particular amino acid in the query disordered proteins/regions set, and *P*_*order*_ denotes the corresponding percentage of ordered proteins/regions in the O_PDB_S25 dataset.

### Orthologous group and phylogenetic analyses

Twenty-three SPP proteins were analyzed using Simple Modular Architecture Research Tools (SMART: http://smart.embl-heide lberg.de/)^71^ to detect predicted domains. To analyze primary sequence conservation, multiple sequence alignments were performed using ClustalW^72^, and outputs were presented using BoxShade server version 3.21.

For orthologous group analysis, we obtained fungal proteome data available from the National Center for Biotechnology Information (NCBI) (https://www.ncbi.nlm.nih.gov/) and MycoCosm of Joint Genome Institute (JGI) fungal portal (https://mycocosm.jgi.doe.gov/mycocosm/home) databases. In total, 81 fungal species were selected to cover all major clades of fungi from the subphyla of Ascomycota (Taphrinomycotina, Saccharomycotina, and Pezizomycotina) and Basidiomycota (Agaricomycotina, Pucciniomycotina, and Ustilaginomycotina), as well as representative phyla of Mucoromycota, Zoopagomycota, Chytridiomycota, Blastocladiomycota, and Rozellomycota (Supplementary Data 5d). Orthologous groups were analyzed using OrthoFinder 2.3.14^36^ with the default parameters.

For substitution rate analysis, we followed a previously described method^21^. A representative protein phylogenetically close to orthologous Pezizomycotina proteins was selected from each fungal species (Supplementary Data 5b). Multiple sequence alignments were built for individual proteins using ClustalW^72^ with unified gap penalties, including gap open penalties and gap extension penalties. Maximum likelihood trees were generated for each protein using PhyML. Phylogenetic trees were confirmed by constructing them with a different tool, Molecular Evolutionary Genetics Tool, MEGA 7^73^. The substitution rate was calculated for a given species, *x*, in the corresponding tree, and the evolutionary distance between protein sequences and Pezizomycotina ancestral sequences of *A. oryzae* SPP proteins was estimated by the score $s\left(x\right)=d\left(x\right)-\frac{{\sum }_{y\in {Pezizomycotina}}d(y)}{P}$ (unit: substitutions/site), where *d*(*x*) indicates the branch length from species *x* to the Pezizomycotina root. *P* represents the number of species *y* in Pezizomycotina used in phylogenetic analysis, and therefore $\frac{{\sum }_{y\in {Pezizomycotina}}d(y)}{P}$ represents the average branch length from each species *y* in Pezizomycotina to the Pezizomycotina root.

To further elucidate orthologous relationships, phylogenetic trees were generated using all proteins classified into the same orthologous group. Multiple sequence alignments were performed, and unrooted phylogenetic trees for analyzed proteins were constructed using MEGA 7^73^.

### Western blot analysis

Strains were cultured on DPY medium for 18 h. The fungal mycelia were frozen in liquid nitrogen and subsequently ground using a multi-bead shocker. After extraction, cell lysates were incubated with a buffer containing detergent NP-40 [50 mM Tris-HCl (pH 8.0), 200 mM NaCl, 1 mM/mL PMSF, 1× Protease Inhibitor Cocktail (Sigma, St. Louis, MO, USA: P8215), 1% NP-40] to solubilize the membrane proteins. Mycelial extract solubilized with the buffer was incubated in ice for 20 min. After centrifugation at 1000×*g* for 10 min, the supernatant was collected. Cell lysates were separated using SDS-PAGE. The proteins were transferred to Immobilon-P polyvinylidene difluoride (PVDF) membranes (Millipore Sigma, Burlington, MA, USA) using a semidry blotting system (Nihon Eido, Tokyo, Japan). To detect EGFP, Living Colors® A.V. (anti-GFP) monoclonal antibody (1: 2000 dilution, cat # 632380, Clontech) and peroxidase-labeled anti-mouse IgG (H + L) antibody (1: 2000 dilution, cat # PI-2000, Vector Laboratories, Newark, CA, USA) were used as primary and secondary antibodies, respectively. Chemiluminescence was detected using a Western Lightning-ECL system (PerkinElmer, Waltham, MA, USA) and an LAS-4000 image analyzer (GE Healthcare, Buckinghamshire, UK).

### Statistics and reproducibility

The results of at least three independent experiments are presented as means. Error bars represent standard deviations as indicated in the figure legends. Statistical significance was tested using the two-tailed Student’s *t* test in Microsoft Excel; significance is indicated as *P < 0.05 or **P < 0.01.

When representative images are shown (Figs. 2b; 3a–c; 4a, c–f; 5a; 6a, b; 8b, c, f; and Supplementary Figs. 1, 2, 3a, b, 4a–e, 5a, b, 10a, b), three independent hyphae were observed and one representative image is shown.

### Reporting summary

Further information on research design is available in the Nature Portfolio Reporting Summary linked to this article.

## Supplementary information

Supplementary Information

Description of Additional Supplementary Files

Supplementary Data 1

Supplementary Data 2

Supplementary Data 3

Supplementary Data 4

Supplementary Data 5

Supplementary Data 6

Reporting Summary

## Data Availability

All data supporting the findings of the present study are available within this paper, Supplementary Information, and Source Data. Gene sequences of *A. oryzae* can be found in FungiDB (https://fungidb.org/fungidb/app), and the sequences of fungal orthologs can be found in NCBI (https://www.ncbi.nlm.nih.gov/). Source data are provided with this paper.

## Source data

Source Data
