# Supplementary material for: Large-scale identification of genes involved in septal pore plugging in multicellular fungi
Source: Nat Commun. 2023 Mar 17;14:1418. doi: 10.1038/s41467-023-36925-y (PMC10023807; doi:10.1038/s41467-023-36925-y)
Supplement: Supplementary file 1 — Supplementary Information [file 41467_2023_36925_MOESM1_ESM.pdf]

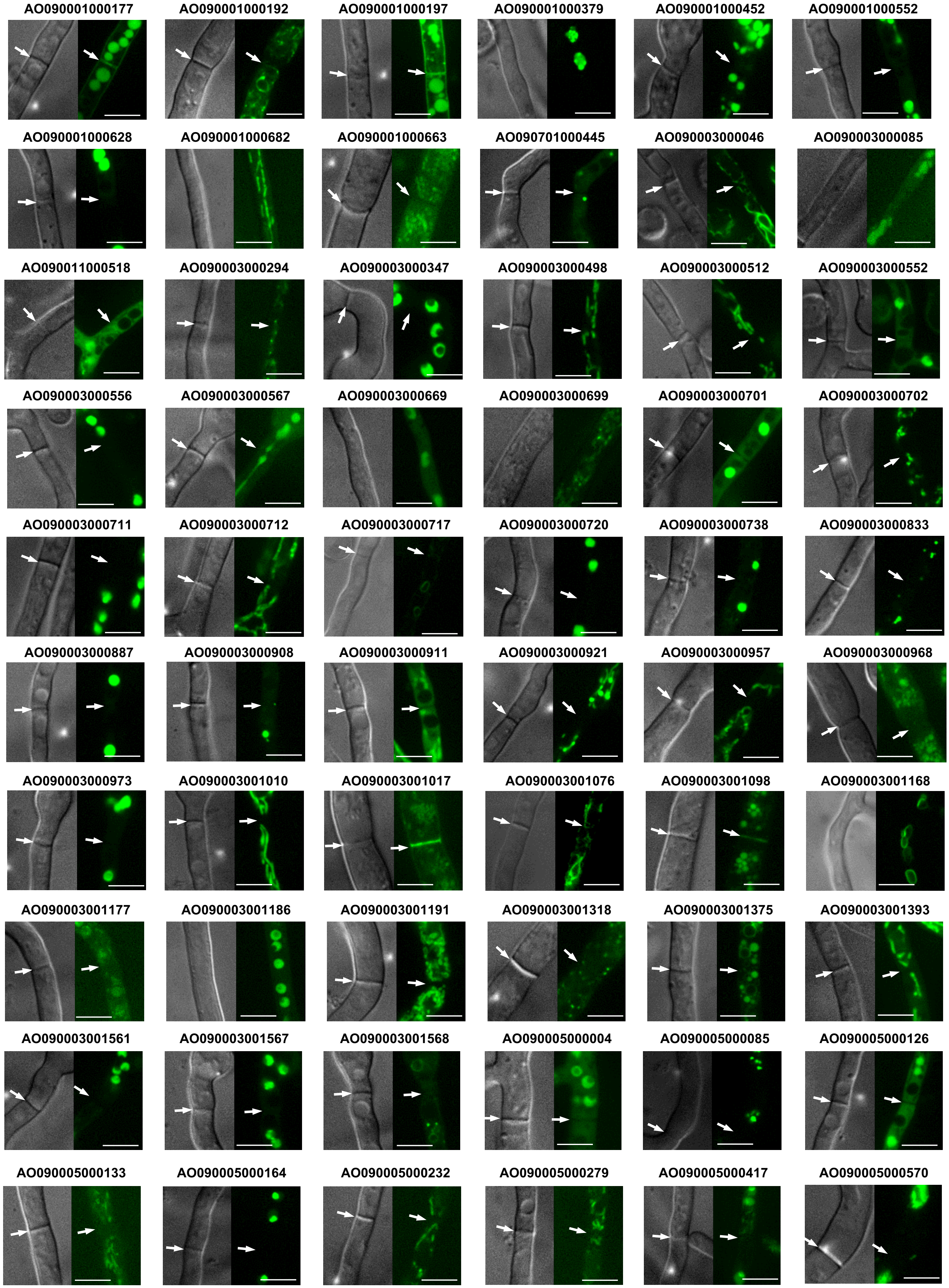
AO090001000177
AO090001000192
AO090001000197
AO090001000379
AO090001000452
AO090001000552
AO090001000628
AO090001000682
AO090001000663
AO090701000445
AO090003000046
AO090003000085
AO090011000518
AO090003000294
AO090003000347
AO090003000498
AO090003000512
AO090003000552
AO090003000556
AO090003000567
AO090003000669
AO090003000699
AO090003000701
AO090003000702
AO090003000711
AO090003000712
AO090003000717
AO090003000720
AO090003000738
AO090003000833
AO090003000887
AO090003000908
AO090003000911
AO090003000921
AO090003000957
AO090003000968
AO090003000973
AO090003001010
AO090003001017
AO090003001076
AO090003001098
AO090003001168
AO090003001177
AO090003001186
AO090003001191
AO090003001318
AO090003001375
AO090003001393
AO090003001561
AO090003001567
AO090003001568
AO090005000004
AO090005000085
AO090005000126
AO090005000133
AO090005000164
AO090005000232
AO090005000279
AO090005000417
AO090005000570

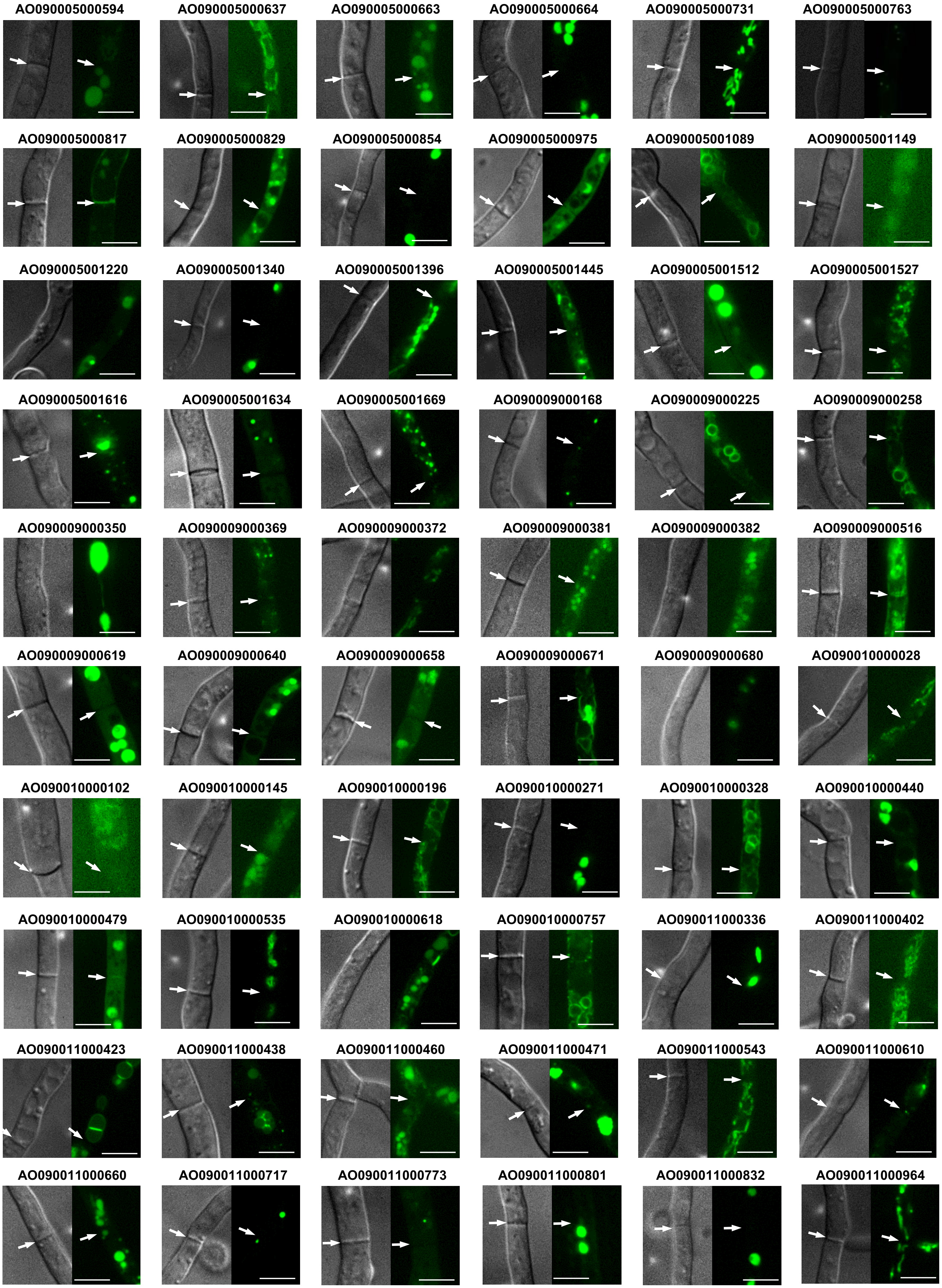
AO090005000594
AO090005000637
AO090005000663
AO090005000664
AO090005000731
AO090005000763
AO090005000817
AO090005000829
AO090005000854
AO090005000975
AO090005001089
AO090005001149
AO090005001220
AO090005001340
AO090005001396
AO090005001445
AO090005001512
AO090005001527
AO090005001616
AO090005001634
AO090005001669
AO090009000168
AO090009000225
AO090009000258
AO090009000350
AO090009000369
AO090009000372
AO090009000381
AO090009000382
AO090009000516
AO090009000619
AO090009000640
AO090009000658
AO090009000671
AO090009000680
AO090010000028
AO090010000102
AO090010000145
AO090010000196
AO090010000271
AO090010000328
AO090010000440
AO090010000479
AO090010000535
AO090010000618
AO090010000757
AO090011000336
AO090011000402
AO090011000423
AO090011000438
AO090011000460
AO090011000471
AO090011000543
AO090011000610
AO090011000660
AO090011000717
AO090011000773
AO090011000801
AO090011000832
AO090011000964

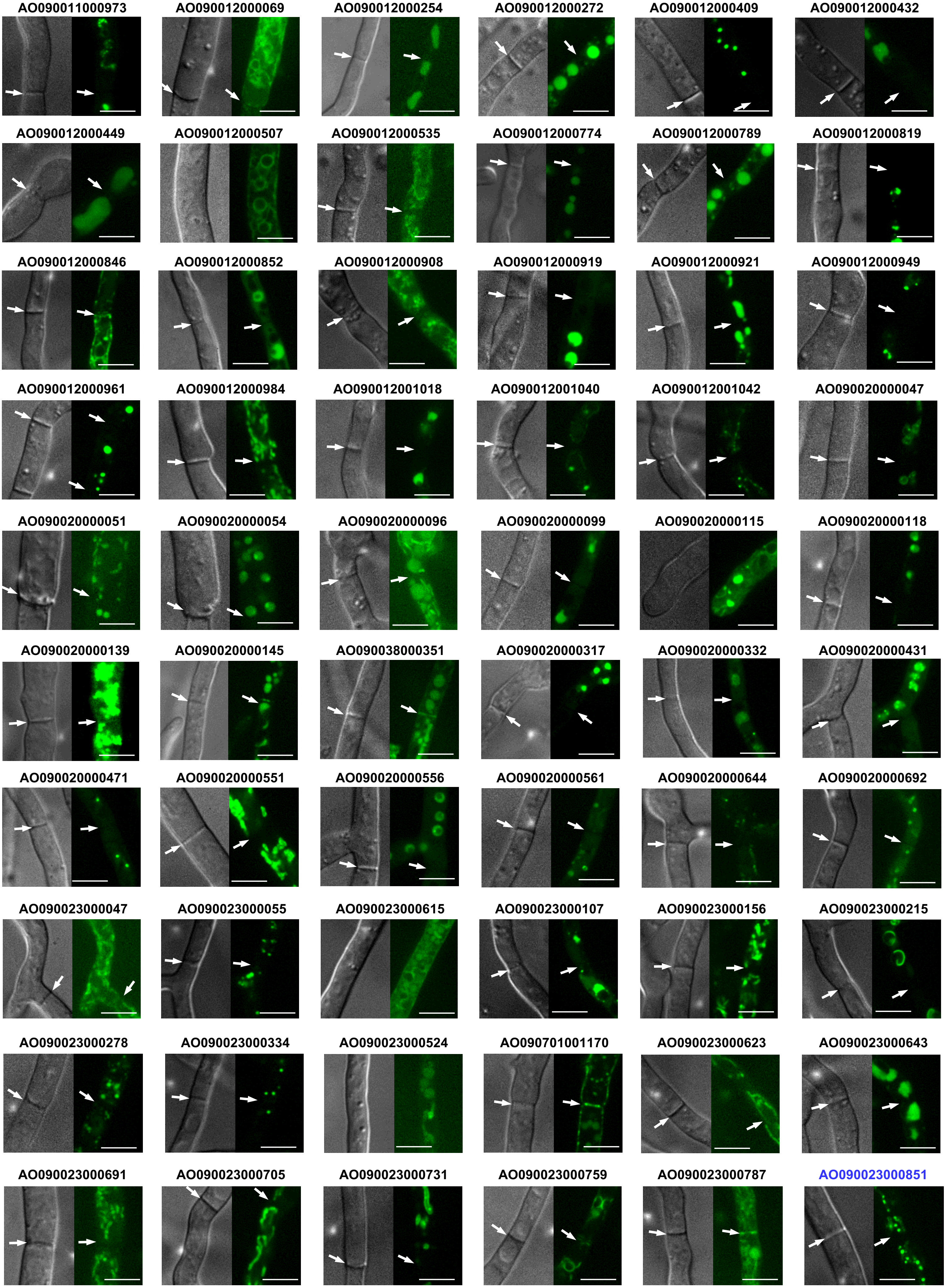
AO090011000973
AO090012000069
AO090012000254
AO090012000272
AO090012000409
AO090012000432
AO090012000449
AO090012000507
AO090012000535
AO090012000774
AO090012000789
AO090012000819
AO090012000846
AO090012000852
AO090012000908
AO090012000919
AO090012000921
AO090012000949
AO090012000961
AO090012000984
AO090012001018
AO090012001040
AO090012001042
AO090020000047
AO090020000051
AO090020000054
AO090020000096
AO090020000099
AO090020000115
AO090020000118
AO090020000139
AO090020000145
AO090038000351
AO090020000317
AO090020000332
AO090020000431
AO090020000471
AO090020000551
AO090020000556
AO090020000561
AO090020000644
AO090020000692
AO090023000047
AO090023000055
AO090023000615
AO090023000107
AO090023000156
AO090023000215
AO090023000278
AO090023000334
AO090023000524
AO090701001170
AO090023000623
AO090023000643
AO090023000691
AO090023000705
AO090023000731
AO090023000759
AO090023000787
AO090023000851

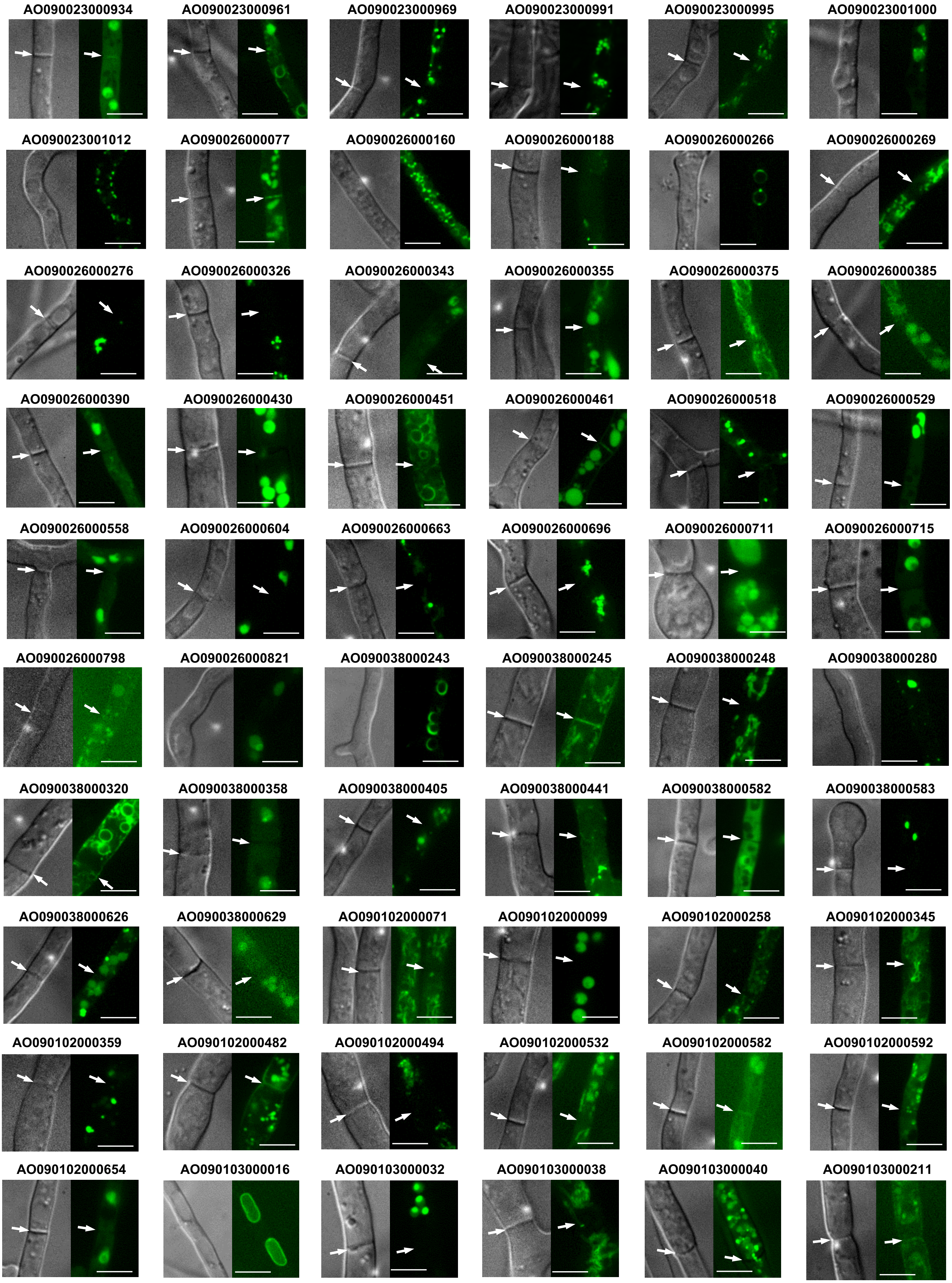
AO090023000934
AO090023000961
AO090023000969
AO090023000991
AO090023000995
AO090023001000
AO090023001012
AO090026000077
AO090026000160
AO090026000188
AO090026000266
AO090026000269
AO090026000276
AO090026000326
AO090026000343
AO090026000355
AO090026000375
AO090026000385
AO090026000390
AO090026000430
AO090026000451
AO090026000461
AO090026000518
AO090026000529
AO090026000558
AO090026000604
AO090026000663
AO090026000696
AO090026000711
AO090026000715
AO090026000798
AO090026000821
AO090038000243
AO090038000245
AO090038000248
AO090038000280
AO090038000320
AO090038000358
AO090038000405
AO090038000441
AO090038000582
AO090038000583
AO090038000626
AO090038000629
AO090102000071
AO090102000099
AO090102000258
AO090102000345
AO090102000359
AO090102000482
AO090102000494
AO090102000532
AO090102000582
AO090102000592
AO090102000654
AO090103000016
AO090103000032
AO090103000038
AO090103000040
AO090103000211

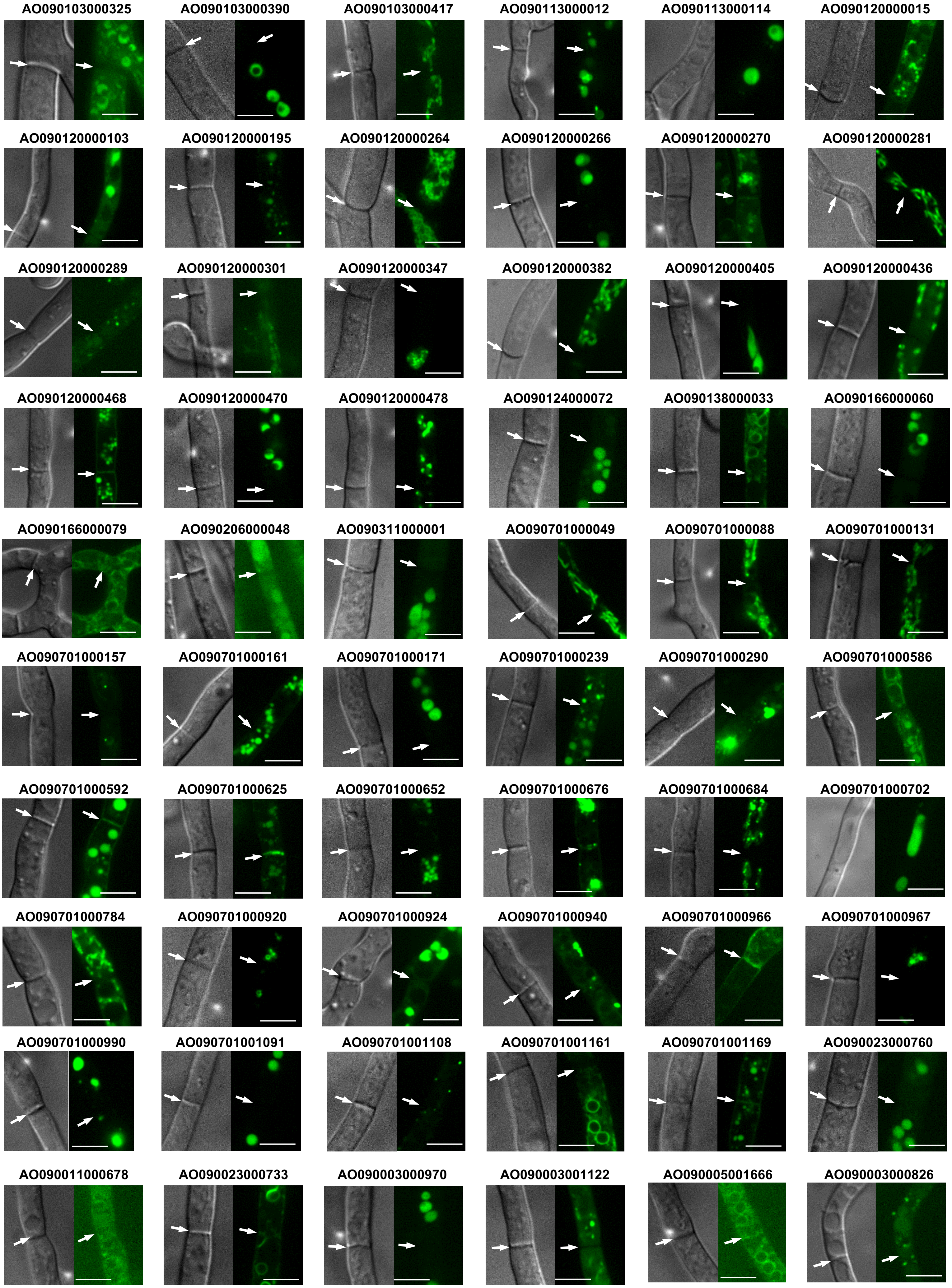
AO090103000325
AO090103000390
AO090103000417
AO090113000012
AO090113000114
AO090120000015
AO090120000103
AO090120000195
AO090120000264
AO090120000266
AO090120000270
AO090120000281
AO090120000289
AO090120000301
AO090120000347
AO090120000382
AO090120000405
AO090120000436
AO090120000468
AO090120000470
AO090120000478
AO090124000072
AO090138000033
AO090166000060
AO090166000079
AO090206000048
AO090311000001
AO090701000049
AO090701000088
AO090701000131
AO090701000157
AO090701000161
AO090701000171
AO090701000239
AO090701000290
AO090701000586
AO090701000592
AO090701000625
AO090701000652
AO090701000676
AO090701000684
AO090701000702
AO090701000784
AO090701000920
AO090701000924
AO090701000940
AO090701000966
AO090701000967
AO090701000990
AO090701001091
AO090701001108
AO090701001161
AO090701001169
AO090023000760
AO090011000678
AO090023000733
AO090003000970
AO090003001122
AO090005001666
AO090003000826

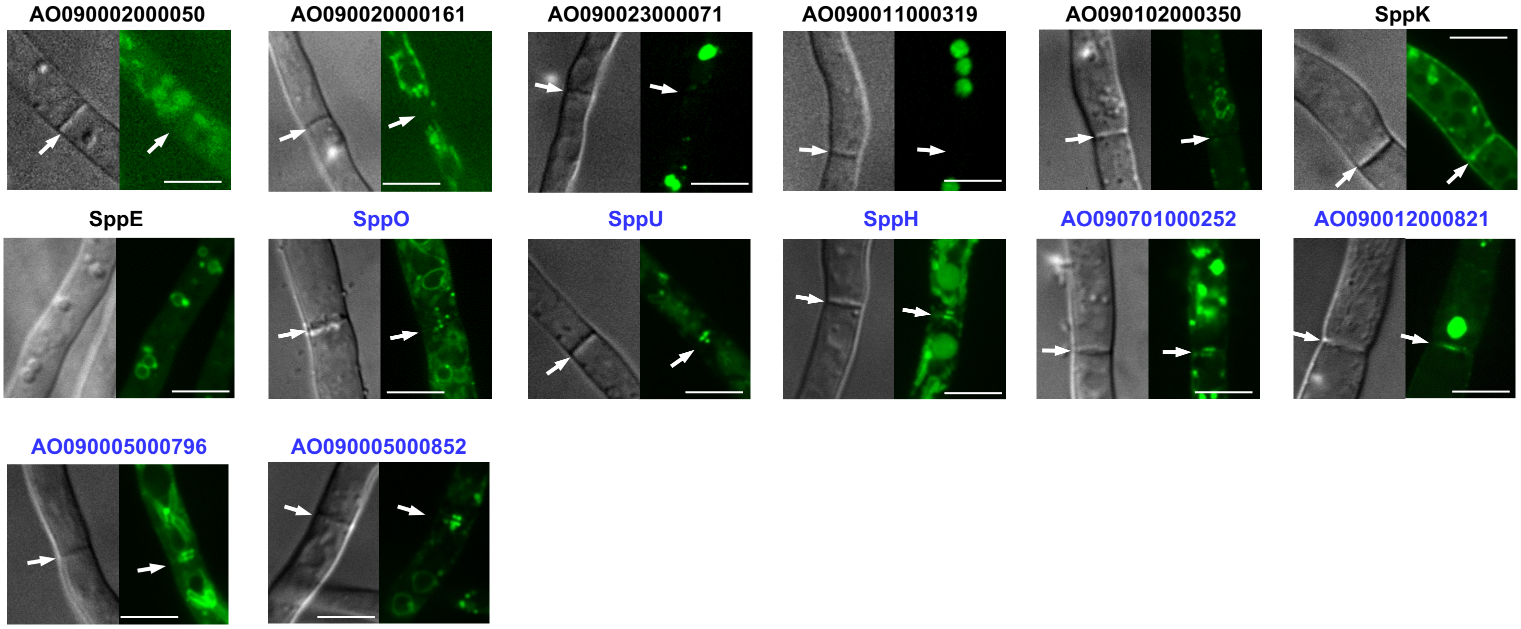


**Supplementary Figure 1: Organelle-like localization of 314 candidate proteins**. The panel labels are Gene IDs or SPP protein names, and the proteins localizing to the septum are shown in blue. Arrows indicate septa. Scale bars, 5 µm.

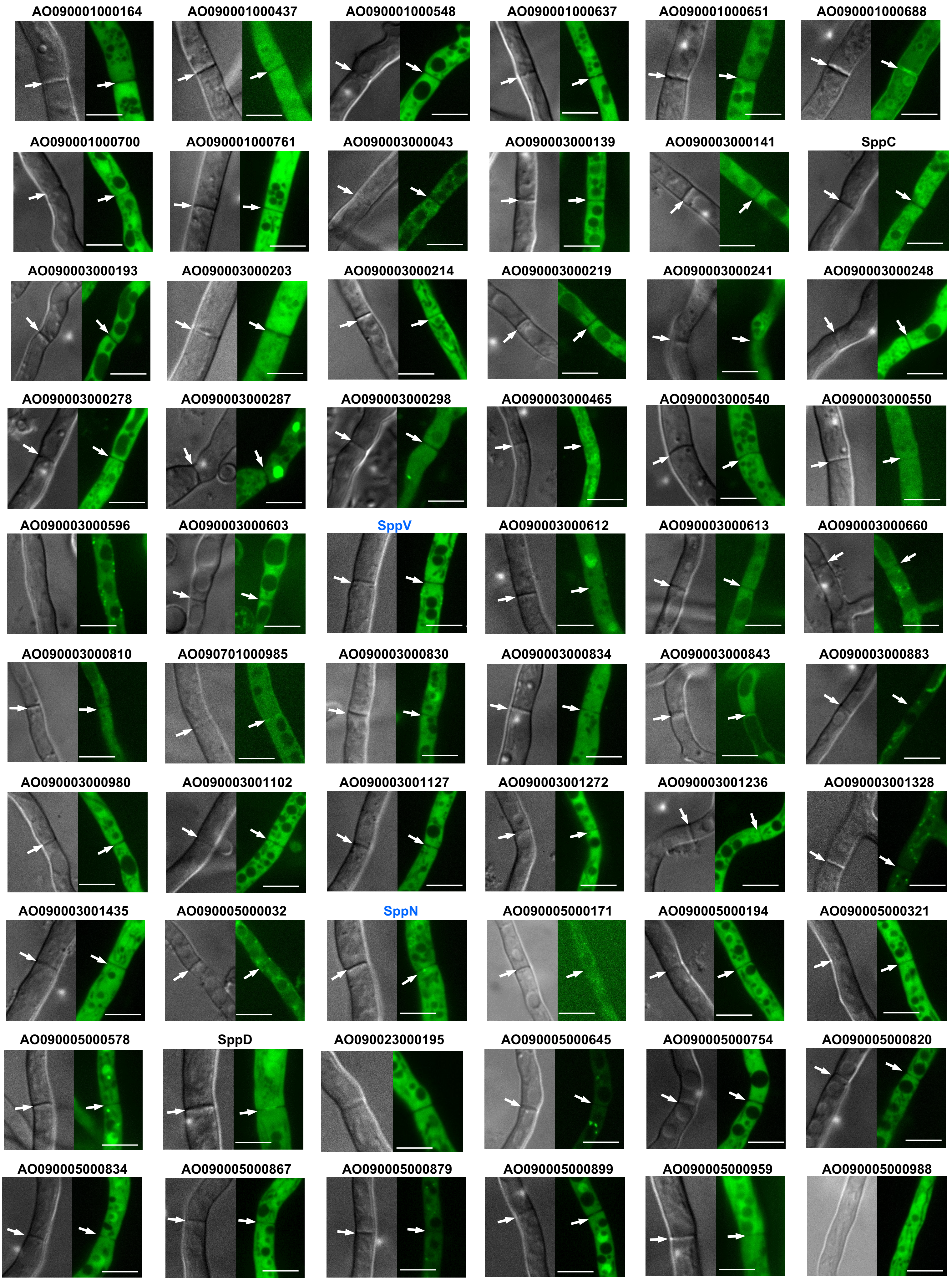
AO090001000164
AO090001000437
AO090001000548
AO090001000637
AO090001000651
AO090001000688
AO090001000700
AO090001000761
AO090003000043
AO090003000139
AO090003000141
SppC
AO090003000193
AO090003000203
AO090003000214
AO090003000219
AO090003000241
AO090003000248
AO090003000278
AO090003000287
AO090003000298
AO090003000465
AO090003000540
AO090003000550
AO090003000596
AO090003000603
SppV
AO090003000612
AO090003000613
AO090003000660
AO090003000810
AO090701000985
AO090003000830
AO090003000834
AO090003000843
AO090003000883
AO090003000980
AO090003001102
AO090003001127
AO090003001272
AO090003001236
AO090003001328
AO090003001435
AO090005000032
SppN
AO090005000171
AO090005000194
AO090005000321
AO090005000578
SppD
AO090023000195
AO090005000645
AO090005000754
AO090005000820
AO090005000834
AO090005000867
AO090005000879
AO090005000899
AO090005000959
AO090005000988

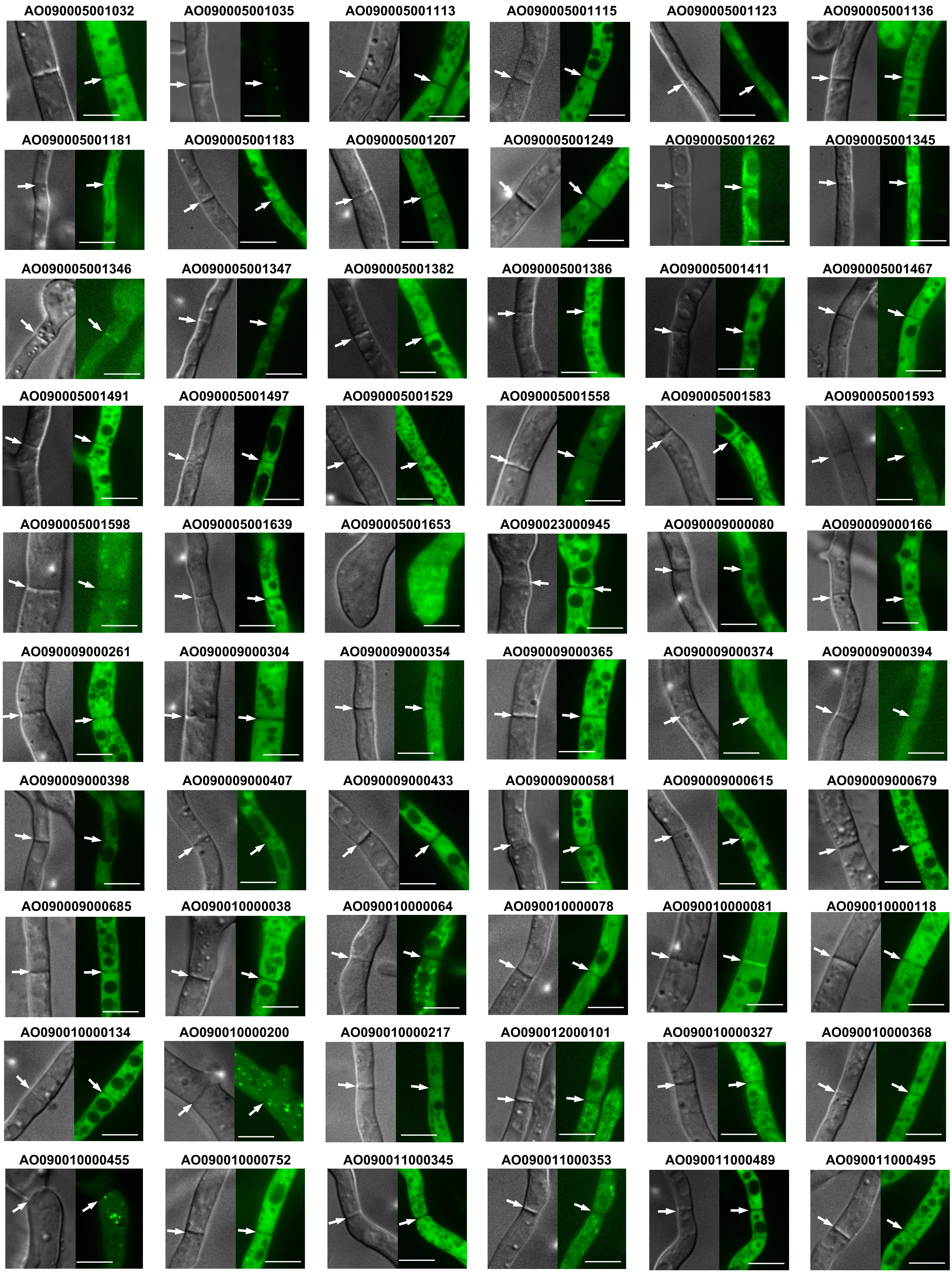
AO090005001032
AO090005001035
AO090005001113
AO090005001115
AO090005001123
AO090005001136
AO090005001181
AO090005001183
AO090005001207
AO090005001249
AO090005001262
AO090005001345
AO090005001346
AO090005001347
AO090005001382
AO090005001386
AO090005001411
AO090005001467
AO090005001491
AO090005001497
AO090005001529
AO090005001558
AO090005001583
AO090005001593
AO090005001598
AO090005001639
AO090005001653
AO090023000945
AO090009000080
AO090009000166
AO090009000261
AO090009000304
AO090009000354
AO090009000365
AO090009000374
AO090009000394
AO090009000398
AO090009000407
AO090009000433
AO090009000581
AO090009000615
AO090009000679
AO090009000685
AO090010000038
AO090010000064
AO090010000078
AO090010000081
AO090010000118
AO090010000134
AO090010000200
AO090010000217
AO090012000101
AO090010000327
AO090010000368
AO090010000455
AO090010000752
AO090011000345
AO090011000353
AO090011000489
AO090011000495

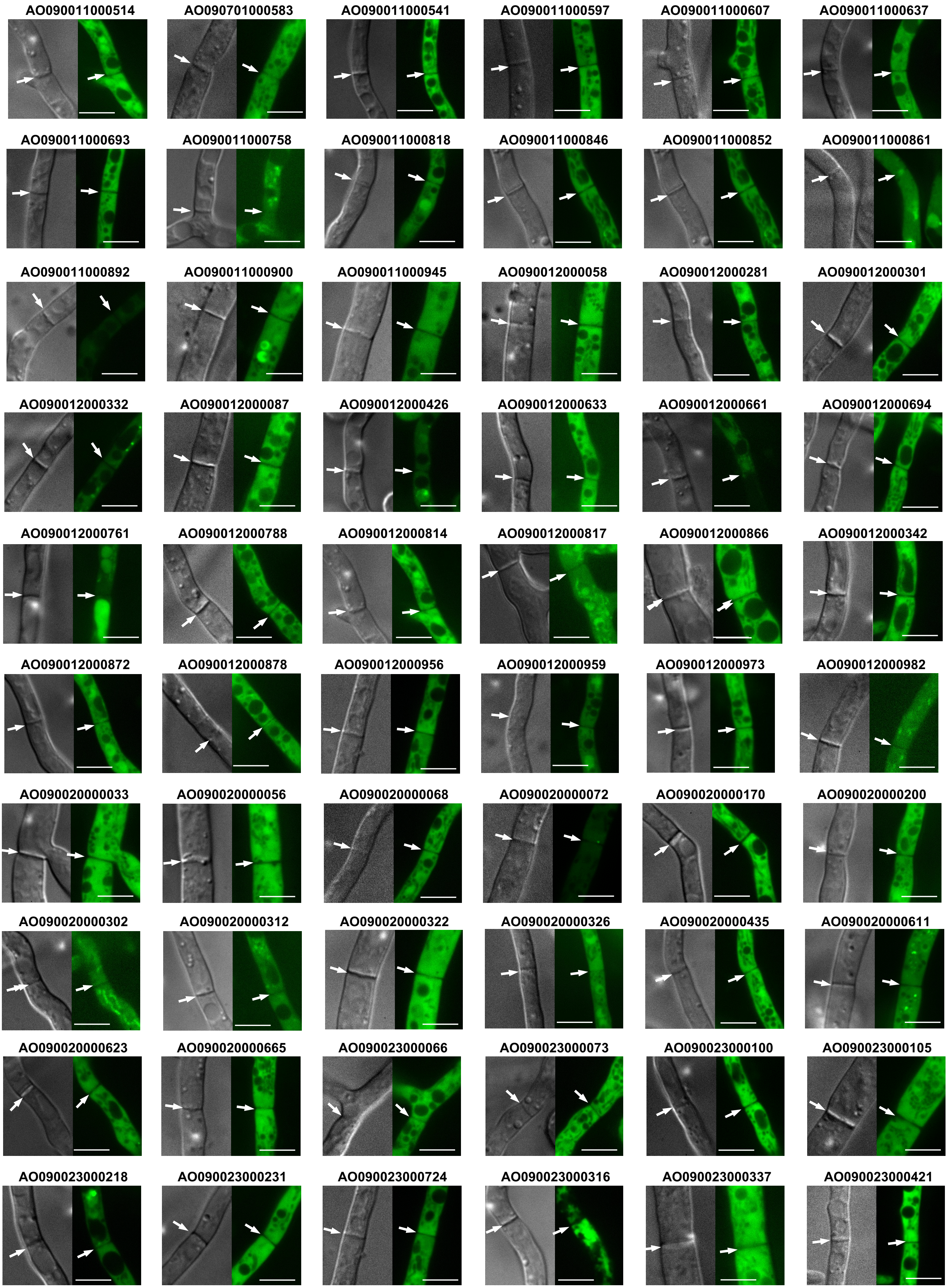
AO090011000514
AO090701000583
AO090011000541
AO090011000597
AO090011000607
AO090011000637
AO090011000693
AO090011000758
AO090011000818
AO090011000846
AO090011000852
AO090011000861
AO090011000892
AO090011000900
AO090011000945
AO090012000058
AO090012000281
AO090012000301
AO090012000332
AO090012000087
AO090012000426
AO090012000633
AO090012000661
AO090012000694
AO090012000761
AO090012000788
AO090012000814
AO090012000817
AO090012000866
AO090012000342
AO090012000872
AO090012000878
AO090012000956
AO090012000959
AO090012000973
AO090012000982
AO090020000033
AO090020000056
AO090020000068
AO090020000072
AO090020000170
AO090020000200
AO090020000302
AO090020000312
AO090020000322
AO090020000326
AO090020000435
AO090020000611
AO090020000623
AO090020000665
AO090023000066
AO090023000073
AO090023000100
AO090023000105
AO090023000218
AO090023000231
AO090023000724
AO090023000316
AO090023000337
AO090023000421

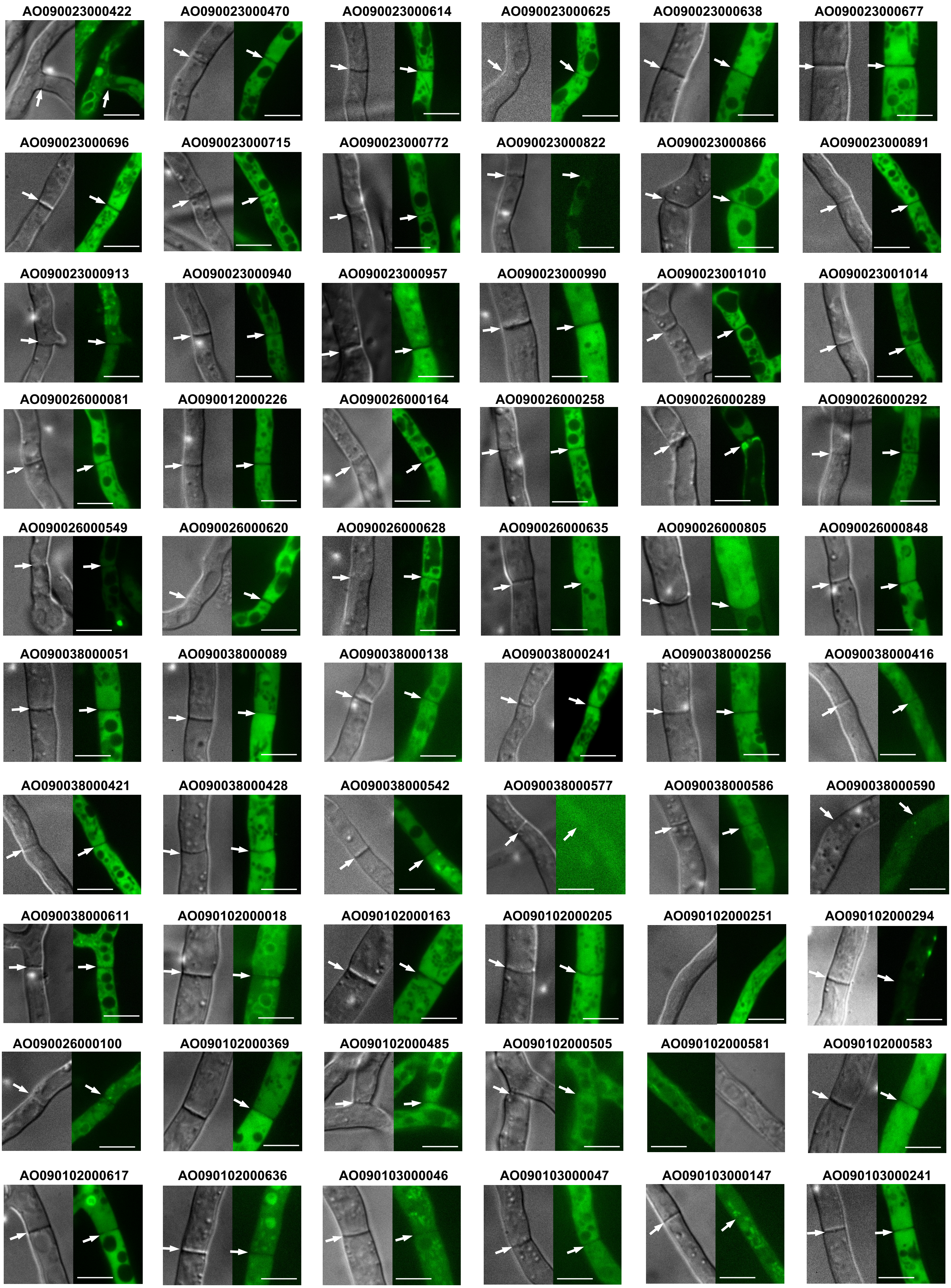
AO090023000422
AO090023000470
AO090023000614
AO090023000625
AO090023000638
AO090023000677
AO090023000696
AO090023000715
AO090023000772
AO090023000822
AO090023000866
AO090023000891
AO090023000913
AO090023000940
AO090023000957
AO090023000990
AO090023001010
AO090023001014
AO090026000081
AO090012000226
AO090026000164
AO090026000258
AO090026000289
AO090026000292
AO090026000549
AO090026000620
AO090026000628
AO090026000635
AO090026000805
AO090026000848
AO090038000051
AO090038000089
AO090038000138
AO090038000241
AO090038000256
AO090038000416
AO090038000421
AO090038000428
AO090038000542
AO090038000577
AO090038000586
AO090038000590
AO090038000611
AO090102000018
AO090102000163
AO090102000205
AO090102000251
AO090102000294
AO090026000100
AO090102000369
AO090102000485
AO090102000505
AO090102000581
AO090102000583
AO090102000617
AO090102000636
AO090103000046
AO090103000047
AO090103000147
AO090103000241

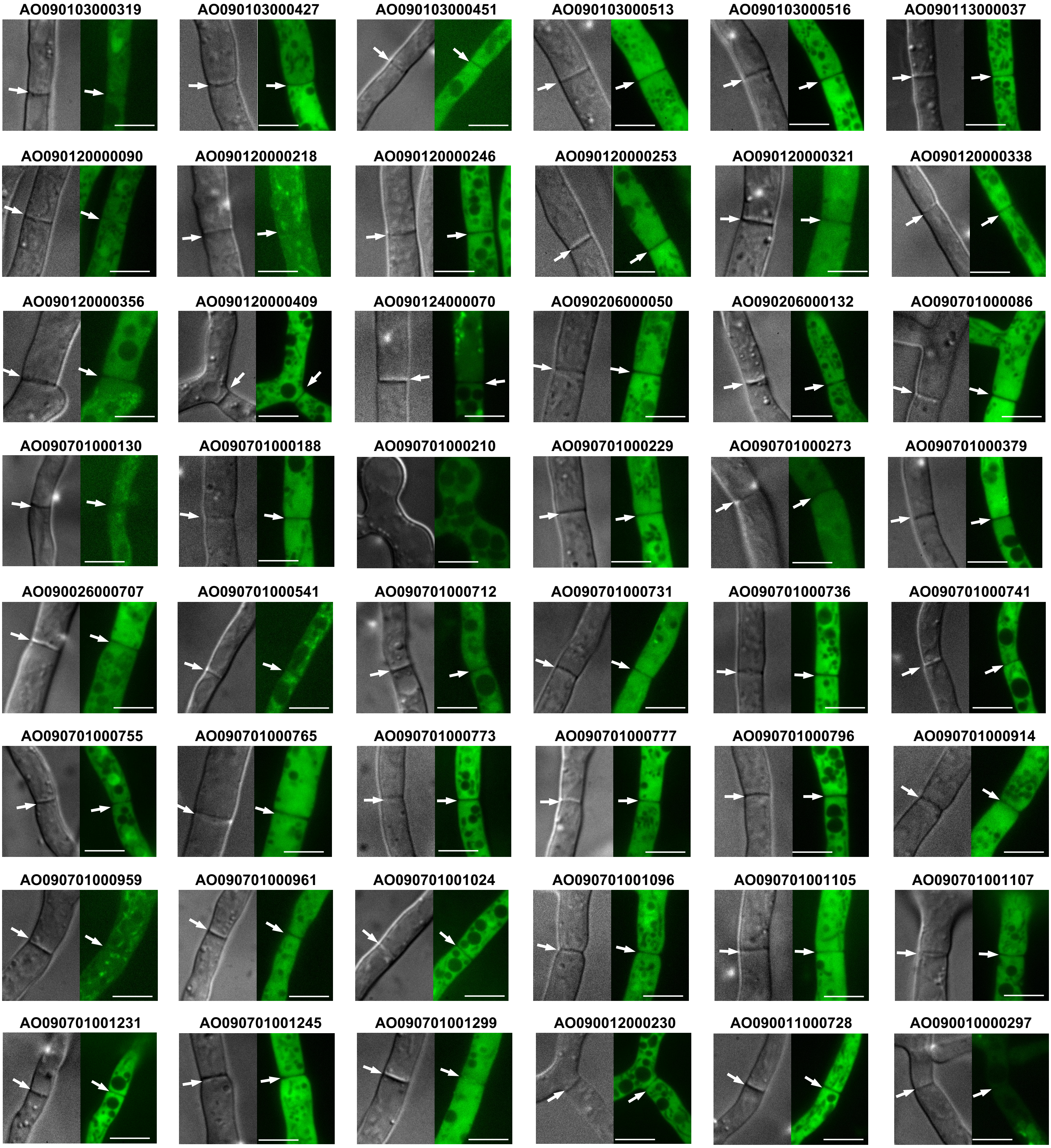


**Supplementary Figure 2: Cytoplasmic localization of 288 candidate proteins**. Gene IDs for the proteins are represented for the corresponding micrographs, and the proteins localizing to the septum are shown in blue. Arrows indicate septa. Scale bars, 5 μm.

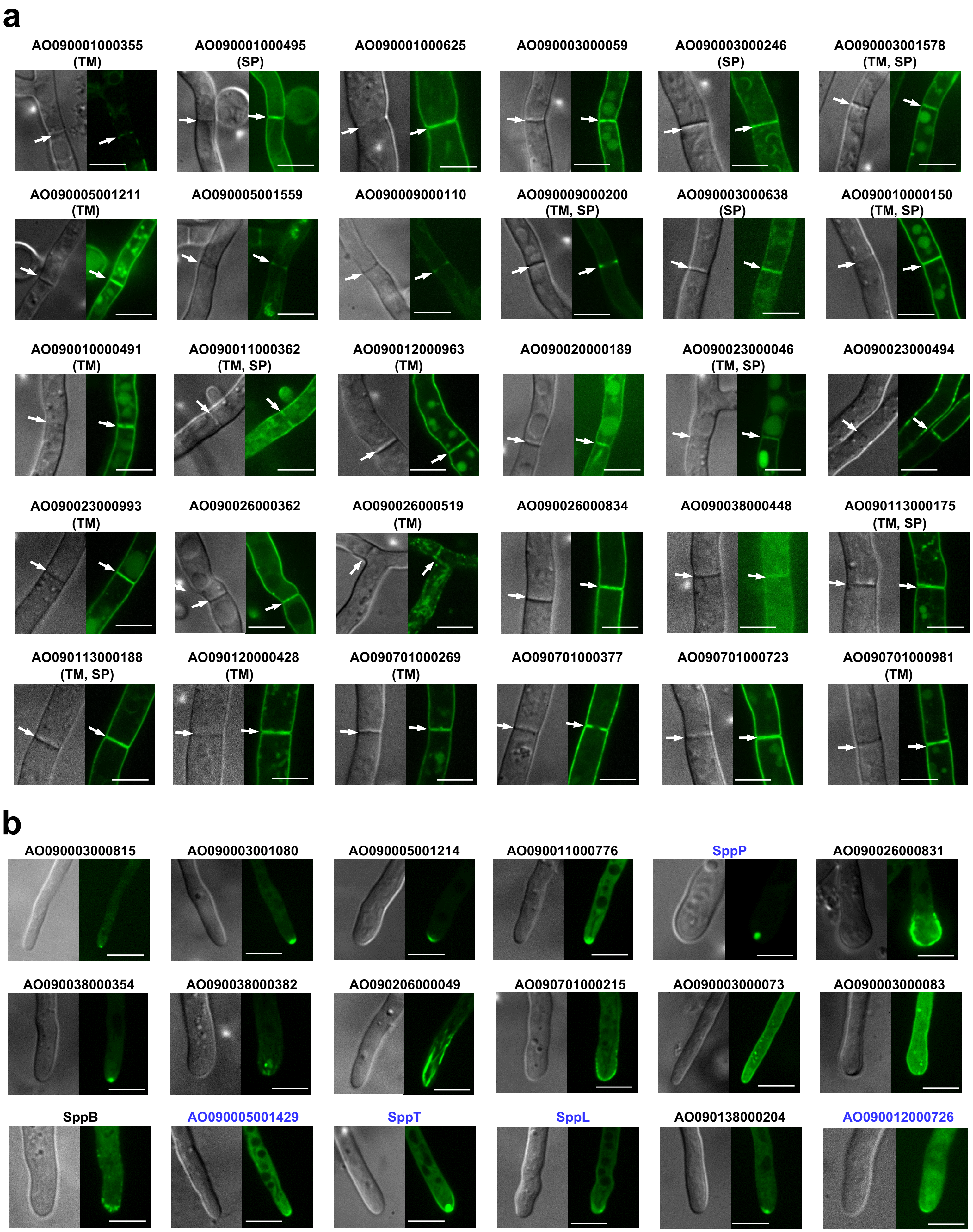


**Supplementary Figure 3: Peripheral and hyphal tip localization of candidate proteins**. **a** Peripheral localization of 30 proteins. TM and SP under gene IDs indicate transmembrane domain and signal peptide, respectively. **b** Hyphal tip localization of 18 candidate proteins. The proteins localizing to the septum under normal growth condition are shown in blue. Arrows indicate septa. Scale bars, 5 µm.

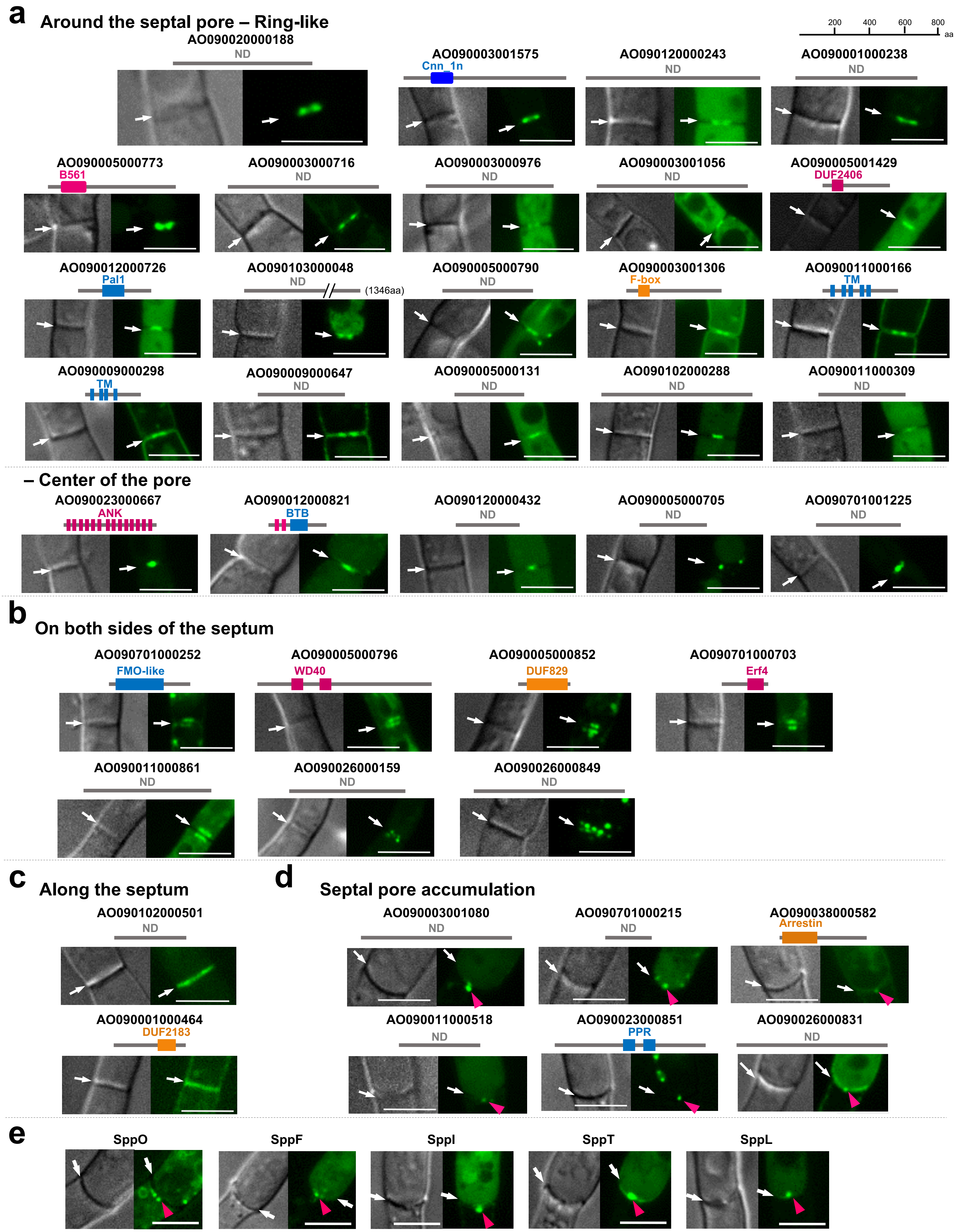


**Supplementary Figure 4: Septum and septal pore localization of candidate proteins.** **a** Protein localizations around the septal pore. **b** Protein localizations on both sides of the septum. **c** Protein localizations along the septum. **d** Accumulation of non-septal proteins at the septal pore upon hyphal wounding. **e** Accumulation of non-septal SPP proteins at the septal pore upon hyphal wounding induced by hypotonic shock. Arrows indicate septa, and arrowheads represent septal pore accumulation. Scale bars, 5 μm. Diagrams of protein domain structures are shown for individual proteins. ND; no predicted domains.

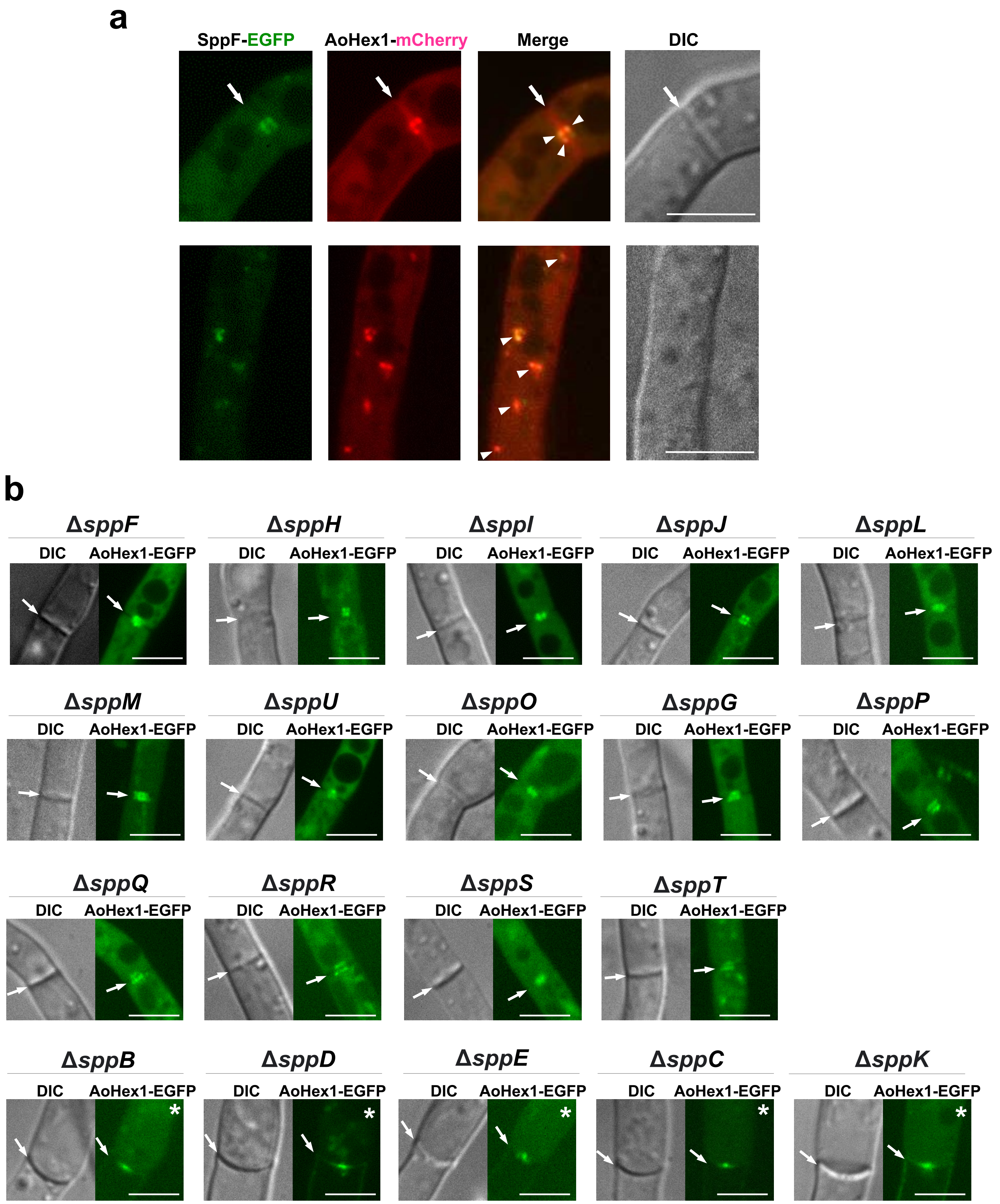


**Supplementary Figure 5: Relationships between SPP proteins and Woronin bodies. a** Woronin bodies visualized using AoHex1-mCherry and SppF with EGFP. Arrows indicate septa, and arrowheads indicate colocalization. Scale bars, 5 µm. **b** Woronin bodies visualized using AoHex1-EGFP expressed in *spp* deletion backgrounds. Arrows indicate septa, and asterisks indicate hyphae subjected to hyphal wounding induced by hypotonic shock. Scale bars, 5 µm.

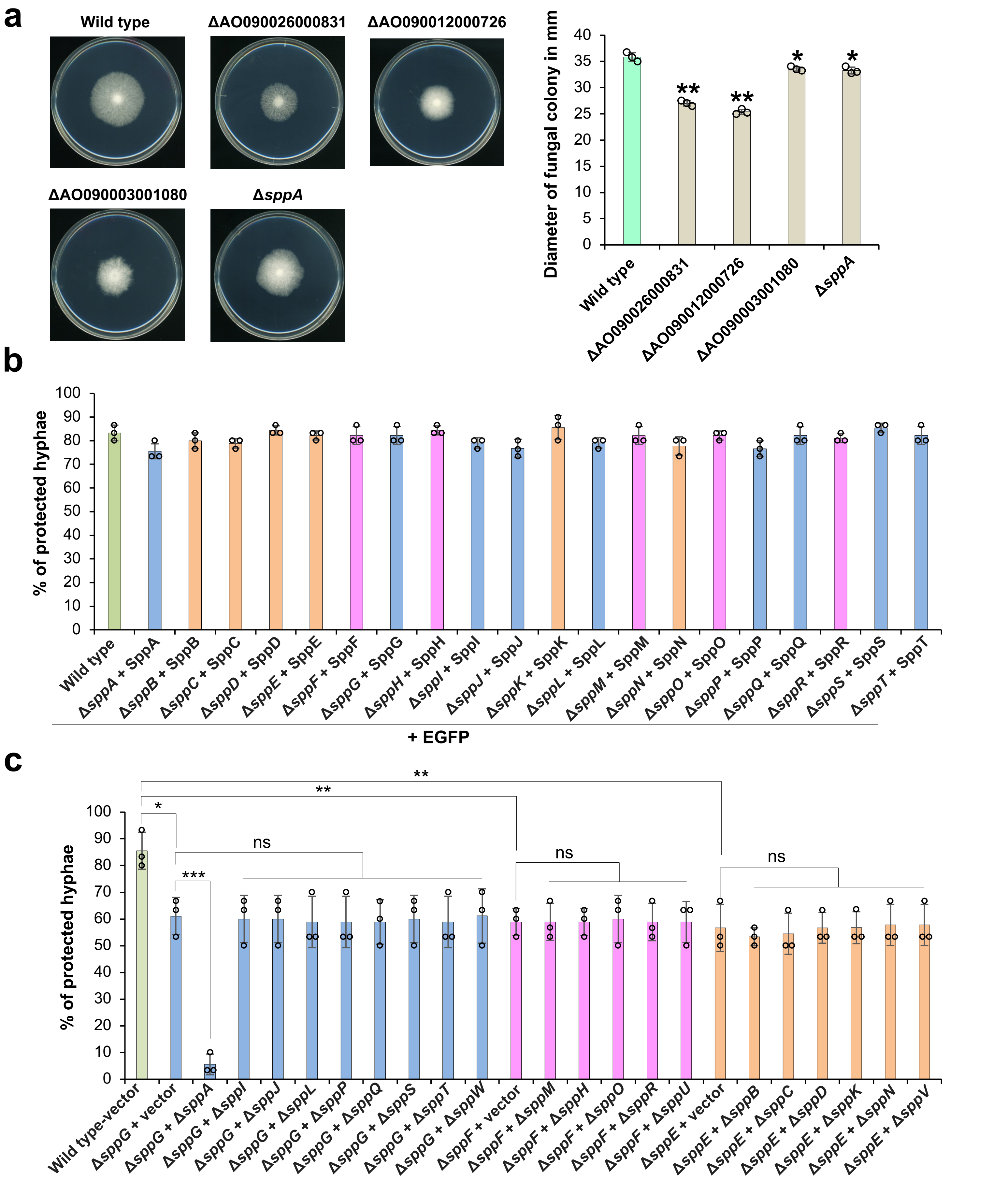


**Supplementary Figure 6: Functionality of EGFP-fused SPP proteins.** **a** Colony growth from conidial suspensions. Three independent experiments were performed. The data are presented as the mean of replicate experiments, and error bars represent standard deviations. Statistical significance was tested using two-tailed Student *t*-test: $*p < 0.05$, $**p < 0.01$. Source data are provided as a Source Data file. **b** Protection of flanking cells from cytoplasmic loss upon hyphal tip bursting under the expression of EGFP-tagged SPP proteins in deletion backgrounds. **c** Protection of flanking cells from cytoplasmic loss upon hyphal tip bursting in double deletion strains. For **b** and **c**, thirty randomly selected hyphae showing hyphal tip bursting were observed in each experiment. Three independent experiments were performed, and the percentage of hyphae protected from the excessive loss of cytoplasm is shown in the graph. The data are presented as the mean of replicate experiments, and error bars represent standard deviations. Statistical analysis was performed between the wild type and three representative single deletions representative for each localization category or between the double deletions and the corresponding representative single deletion. Statistical significance was tested using two-tailed Student *t*-test: $*p < 0.05$, $**p < 0.01$, ns = not significant. Source data are provided as a Source Data file.

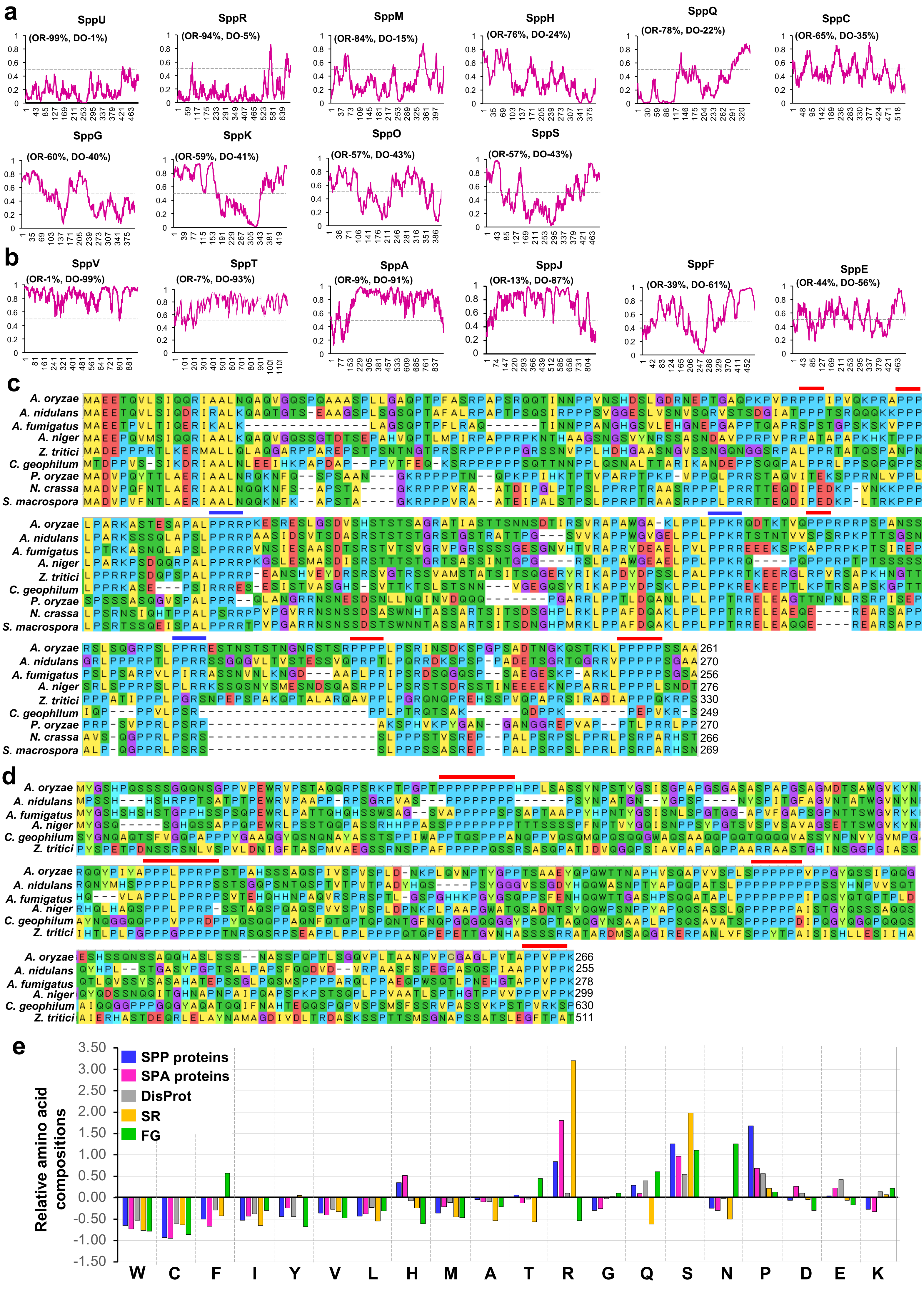
a
SppU
(OR-99%, DO-1%)
SppR
(OR-94%, DO-5%)
SppM
(OR-84%, DO-15%)
SppH
(OR-76%, DO-24%)
SppQ
(OR-78%, DO-22%)
SppC
(OR-65%, DO-35%)
SppG
(OR-60%, DO-40%)
SppK
(OR-59%, DO-41%)
SppO
(OR-57%, DO-43%)
SppS
(OR-57%, DO-43%)
b
SppV
(OR-1%, DO-99%)
SppT
(OR-7%, DO-93%)
SppA
(OR-9%, DO-91%)
SppJ
(OR-13%, DO-87%)
SppF
(OR-39%, DO-61%)
SppE
(OR-44%, DO-56%)
c
A. oryzae
A. nidulans
A. fumigatus
A. niger
Z. tritici
C. geophilum
P. oryzae
N. crassa
S. macrospora
261
270
256
276
330
249
270
266
269
d
A. oryzae
A. nidulans
A. fumigatus
A. niger
C. geophilum
Z. tritici
266
255
278
299
630
511
e
SPP proteins
SPA proteins
DisProt
SR
FG
Relative amino acid compositions
W C F I Y V L H M A T R G Q S N P D E K

**Supplementary Fig. 7: Prediction of disordered regions in SPP proteins. a** SPP proteins containing a higher portion of the ordered region. **b** SPP proteins containing a higher portion of the disordered region. In the graph for the prediction of disordered regions in IUPred2A, the y axis indicates the predicted probability of disorder, and the x-axis represents the amino acid sequence. OR; ordered, DO; disordered. **c, d** Multiple sequence alignment of the N-terminal disordered regions of SppB and SppN. Amino acid sequences of orthologous proteins were retrieved from NCBI using protein BLAST. Multiple sequence alignment was performed using ClustalW in MEGA X. Red lines above the sequence denote conserved poly-proline motifs. Blue lines denote conserved PPRR/PPKR motifs. **e** Relative amino acid composition in disordered regions of SPP and SPA proteins, disordered proteins of serine/arginine-rich (SR) splicing factors and phenylalanine/glycine repeats (FG) nucleoporins, and disordered proteins/regions from the DisProt database. Source data are provided as a Source Data file.

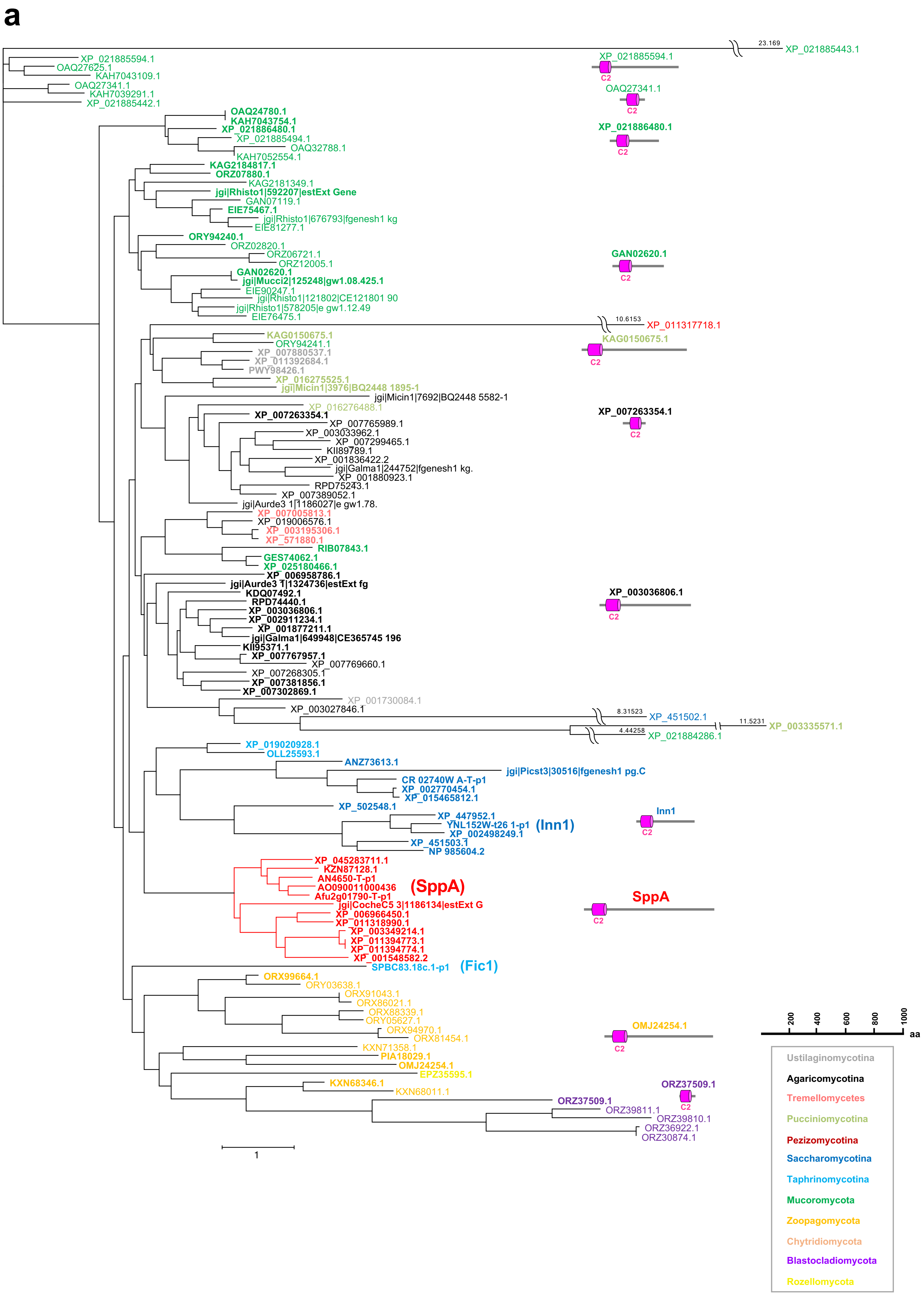
a
23.169
XP_021885443.1
XP_021885594.1
OAQ27625.1
KAH7043109.1
OAQ27341.1
KAH7039291.1
XP_021885442.1
OAQ24780.1
KAH7043754.1
XP_021886480.1
XP_021885494.1
OAQ32788.1
KAH7052554.1
KAG2184817.1
ORZ07880.1
KAG2181349.1
jgi|Rhisto1|592207|estExt Gene
GAN07119.1
EIE75467.1
jgi|Rhisto1|676793|fgenesh1 kg
EIE81277.1
ORY94240.1
ORZ02820.1
ORZ06721.1
ORZ12005.1
GAN02620.1
jgi|Mucci2|125248|gw1.08.425.1
EIE90247.1
jgi|Rhisto1|121802|CE121801 90
jgi|Rhisto1|578205|e gw1.12.49
EIE76475.1
10.6153
XP_011317718.1
KAG0150675.1
ORY94241.1
XP_007880537.1
XP_011392684.1
PWY98426.1
XP_016275525.1
jgi|Micin1|3976|BQ2448 1895-1
jgi|Micin1|7692|BQ2448 5582-1
XP_016276488.1
XP_007263354.1
XP_007765989.1
XP_003033962.1
XP_007299465.1
KII89789.1
XP_001836422.2
jgi|Galma1|244752|fgenesh1 kg.
XP_001880923.1
RPD75243.1
XP_007389052.1
jgi|Aurde3 1|1186027|e gw1.78.
XP_007005813.1
XP_019006576.1
XP_003195306.1
XP_571880.1
RIB07843.1
GES74062.1
XP_025180466.1
XP_006958786.1
jgi|Aurde3 1|1324736|estExt fg
KDQ07492.1
RPD74440.1
XP_003036806.1
XP_002911234.1
XP_001877211.1
jgi|Galma1|649948|CE365745 196
KII95371.1
XP_007767957.1
XP_007769660.1
XP_007268305.1
XP_007381856.1
XP_007302869.1
XP_001730084.1
XP_003027846.1
8.31523
XP_451502.1
11.5231
XP_003335571.1
4.44258
XP_021884286.1
XP_019020928.1
OLL25593.1
ANZ73613.1
jgi|Picst3|30516|fgenesh1 pg.C
CR 02740W A-T-p1
XP_002770454.1
XP_015465812.1
XP_502548.1
XP_447952.1
YNL152W-t26 1-p1
(Inn1)
XP_002498249.1
XP_451503.1
NP 985604.2
XP_045283711.1
KZN87128.1
AN4650-T-p1
(SppA)
AO090011000436
Afu2g01790-T-p1
jgi|CocheC5 3|1186134|estExt G
XP_006966450.1
XP_011318990.1
XP_003349214.1
XP_011394773.1
XP_011394774.1
XP_001548582.2
SPBC83.18c.1-p1
(Fic1)
ORX99664.1
ORY03638.1
ORX91043.1
ORX86021.1
ORX88339.1
ORY05627.1
ORX94970.1
ORX81454.1
KXN71358.1
PIA18029.1
OMJ24254.1
EPZ35595.1
KXN68346.1
KXN68011.1
ORZ37509.1
ORZ39811.1
ORZ39810.1
ORZ36922.1
ORZ30874.1
1
XP_021885594.1
C2
OAQ27341.1
C2
XP_021886480.1
C2
GAN02620.1
C2
KAG0150675.1
C2
XP_007263354.1
C2
XP_003036806.1
C2
Inn1
C2
SppA
C2
OMJ24254.1
C2
ORZ37509.1
C2
200
400
600
800
1000
aa
Ustilaginomycotina
Agaricomycotina
Tremellomycetes
Pucciniomycotina
Pezizomycotina
Saccharomycotina
Taphrinomycotina
Mucoromycota
Zoopagomycota
Chytridiomycota
Blastocladiomycota
Rozellomycota

b

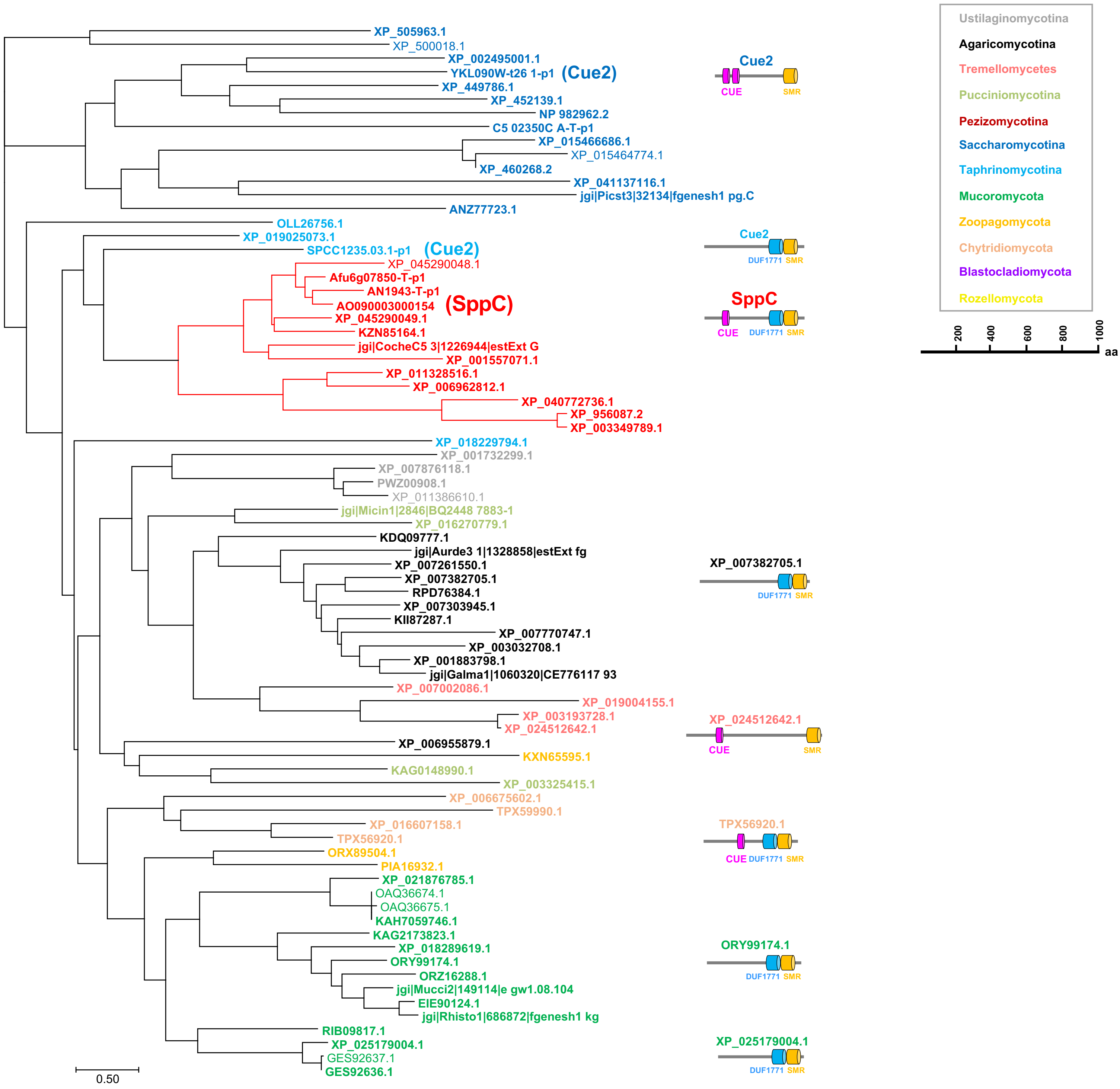
XP_505963.1
XP_500018.1
XP_002495001.1
YKL090W-t26 1-p1 (Cue2)
XP_449786.1
XP_452139.1
NP 982962.2
C5 02350C A-T-p1
XP_015466686.1
XP_015464774.1
XP_460268.2
XP_041137116.1
jgi|Picst3|32134|fgenesh1 pg.C
ANZ77723.1
OLL26756.1
XP_019025073.1
SPCC1235.03.1-p1 (Cue2)
XP_045290048.1
Afu6g07850-T-p1
AN1943-T-p1
AO090003000154 (SppC)
XP_045290049.1
KZN85164.1
jgi|CocheC5 3|1226944|estExt G
XP_001557071.1
XP_011328516.1
XP_006962812.1
XP_040772736.1
XP_956087.2
XP_003349789.1
XP_018229794.1
XP_001732299.1
XP_007876118.1
PWZ00908.1
XP_011386610.1
jgi|Micin1|2846|BQ2448 7883-1
XP_016270779.1
KDQ09777.1
jgi|Aurde3 1|1328858|estExt fg
XP_007261550.1
XP_007382705.1
RPD76384.1
XP_007303945.1
KII87287.1
XP_007770747.1
XP_003032708.1
XP_001883798.1
jgi|Galma1|1060320|CE776117 93
XP_007002086.1
XP_019004155.1
XP_003193728.1
XP_024512642.1
XP_006955879.1
KXN65595.1
KAG0148990.1
XP_003325415.1
XP_006675602.1
TPX59990.1
XP_016607158.1
TPX56920.1
ORX89504.1
PIA16932.1
XP_021876785.1
OAQ36674.1
OAQ36675.1
KAH7059746.1
KAG2173823.1
XP_018289619.1
ORY99174.1
ORZ16288.1
jgi|Mucci2|149114|e gw1.08.104
EIE90124.1
jgi|Rhisto1|686872|fgenesh1 kg
RIB09817.1
XP_025179004.1
GES92637.1
GES92636.1
0.50
Cue2
CUE
SMR
Cue2
DUF1771
SMR
SppC
CUE
DUF1771
SMR
XP_007382705.1
DUF1771
SMR
XP_024512642.1
CUE
SMR
TPX56920.1
CUE
DUF1771
SMR
ORY99174.1
DUF1771
SMR
XP_025179004.1
DUF1771
SMR
Ustilaginomycotina
Agaricomycotina
Tremellomycetes
Pucciniomycotina
Pezizomycotina
Saccharomycotina
Taphrinomycotina
Mucoromycota
Zoopagomycota
Chytridiomycota
Blastocladiomycota
Rozellomycota
200
400
600
800
1000
aa

c

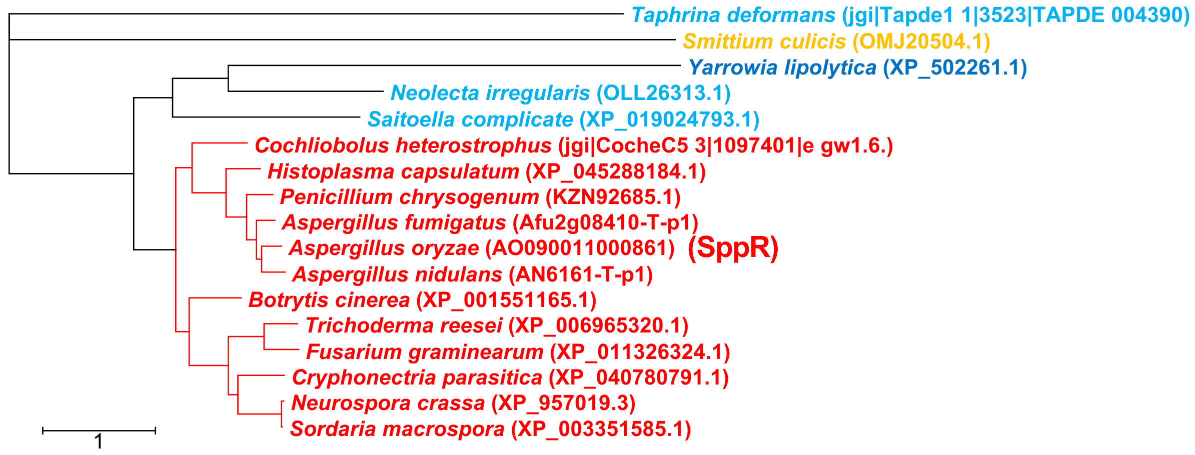
Taphrina deformans (jgi|Tapde1 1|3523|TAPDE 004390)
Smittium culicis (OMJ20504.1)
Yarrowia lipolytica (XP_502261.1)
Neolecta irregularis (OLL26313.1)
Saitoella complicate (XP_019024793.1)
Cochliobolus heterostrophus (jgi|CocheC5 3|1097401|e gw1.6.)
Histoplasma capsulatum (XP_045288184.1)
Penicillium chrysogenum (KZN92685.1)
Aspergillus fumigatus (Afu2g08410-T-p1)
Aspergillus oryzae (AO090011000861) (SppR)
Aspergillus nidulans (AN6161-T-p1)
Botrytis cinerea (XP_001551165.1)
Trichoderma reesei (XP_006965320.1)
Fusarium graminearum (XP_011326324.1)
Cryphonectria parasitica (XP_040780791.1)
Neurospora crassa (XP_957019.3)
Sordaria macrospora (XP_003351585.1)
1

**d**

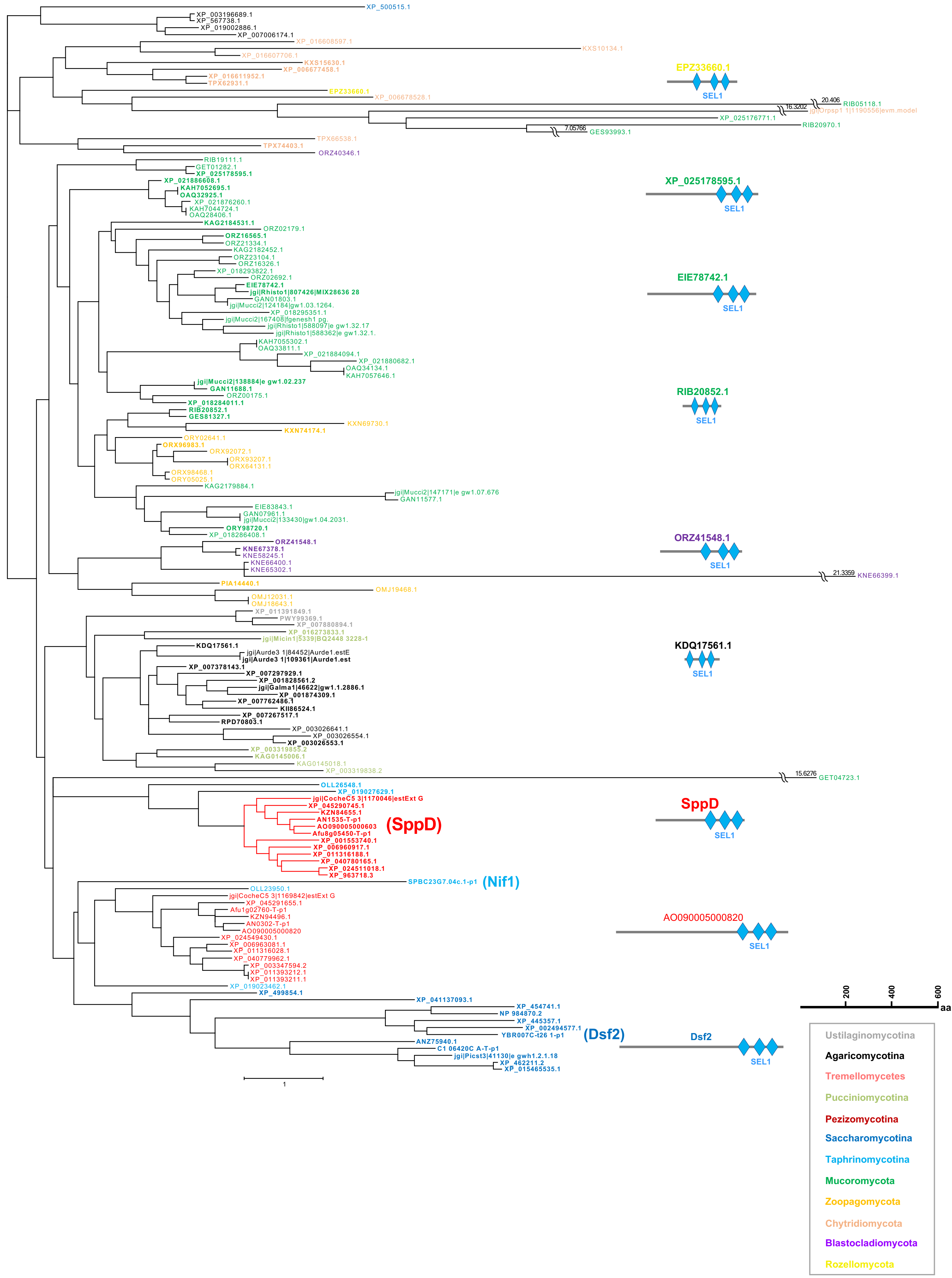

XP_500515.1
XP_003196689.1
XP_567738.1
XP_019002886.1
XP_007006174.1
XP_016608597.1
KXS10134.1
XP_016607706.1
KXS15630.1
XP_006677458.1
XP_016611952.1
TPX62931.1
EPZ33660.1
XP_006678528.1
20.406
RIB05118.1
16.3202
jgi|Orpsp1 1|1190556|evm.model
XP_025176771.1
RIB20970.1
7.05766
GES93993.1
TPX66538.1
TPX74403.1
ORZ40346.1
RIB19111.1
GET01282.1
XP_025178595.1
XP_021886608.1
KAH7052695.1
OAQ32925.1
XP_021876260.1
KAH7044724.1
OAQ28406.1
KAG2184531.1
ORZ02179.1
ORZ16565.1
ORZ21334.1
KAG2182452.1
ORZ23104.1
ORZ16326.1
XP_018293822.1
ORZ02692.1
EIE78742.1
jgi|Rhisto1|807426|MIX28636 28
GAN01803.1
jgi|Mucci2|124184|gw1.03.1264.
XP_018295351.1
jgi|Mucci2|167408|fgenesh1 pg.
jgi|Rhisto1|588097|e gw1.32.17
jgi|Rhisto1|588362|e gw1.32.1.
KAH7055302.1
OAQ33811.1
XP_021884094.1
XP_021880682.1
OAQ34134.1
KAH7057646.1
jgi|Mucci2|138884|e gw1.02.237
GAN11688.1
ORZ00175.1
XP_018284011.1
RIB20852.1
GES81327.1
KXN69730.1
KXN74174.1
ORY02641.1
ORX96983.1
ORX92072.1
ORX93207.1
ORX64131.1
ORX98468.1
ORY05025.1
KAG2179884.1
jgi|Mucci2|147171|e gw1.07.676
GAN11577.1
EIE83843.1
GAN07961.1
jgi|Mucci2|133430|gw1.04.2031.
ORY98720.1
XP_018286408.1
ORZ41548.1
KNE67378.1
KNE58245.1
KNE66400.1
KNE65302.1
21.3359
KNE66399.1
PIA14440.1
OMJ19468.1
OMJ12031.1
OMJ18643.1
XP_011391849.1
PWY99369.1
XP_007880894.1
XP_016273833.1
jgi|Micin1|5339|BQ2448 3228-1
KDQ17561.1
jgi|Aurde3 1|84452|Aurde1.estE
jgi|Aurde3 1|109361|Aurde1.est
XP_007378143.1
XP_007297929.1
XP_001828561.2
jgi|Galma1|46622|gw1.1.2886.1
XP_001874309.1
XP_007762486.1
KII86524.1
XP_007267517.1
RPD70803.1
XP_003026641.1
XP_003026554.1
XP_003026553.1
XP_003319855.2
KAG0145006.1
KAG0145018.1
XP_003319838.2
15.6276
GET04723.1
OLL26548.1
XP_019027629.1
jgi|CocheC5 3|1170046|estExt G
XP_045290745.1
KZN84655.1
AN1535-T-p1
AO090005000603
Afu8g05450-T-p1
XP_001553740.1
XP_006960917.1
XP_011316188.1
XP_040780165.1
XP_024511018.1
XP_963718.3
(SppD)
SPBC23G7.04c.1-p1
(Nif1)
OLL23950.1
jgi|CocheC5 3|1169842|estExt G
XP_045291655.1
Afu1g02760-T-p1
KZN94496.1
AN0302-T-p1
AO090005000820
XP_024549430.1
XP_006963081.1
XP_011316028.1
XP_040779962.1
XP_003347594.2
XP_011393212.1
XP_011393211.1
XP_019023462.1
XP_499854.1
XP_041137093.1
XP_454741.1
NP 984870.2
XP_445357.1
XP_002494577.1
YBR007C-t26 1-p1
(Dsf2)
ANZ75940.1
C1 06420C A-T-p1
jgi|Picst3|41130|e gwh1.2.1.18
XP_462211.2
XP_015465535.1
1
EPZ33660.1
SEL1
XP_025178595.1
SEL1
EIE78742.1
SEL1
RIB20852.1
SEL1
ORZ41548.1
SEL1
KDQ17561.1
SEL1
SppD
SEL1
AO090005000820
SEL1
Dsf2
SEL1
200
400
600
aa
Ustilaginomycotina
Agaricomycotina
Tremellomycetes
Pucciniomycotina
Pezizomycotina
Saccharomycotina
Taphrinomycotina
Mucoromycota
Zoopagomycota
Chytridiomycota
Blastocladiomycota
Rozellomycota

e

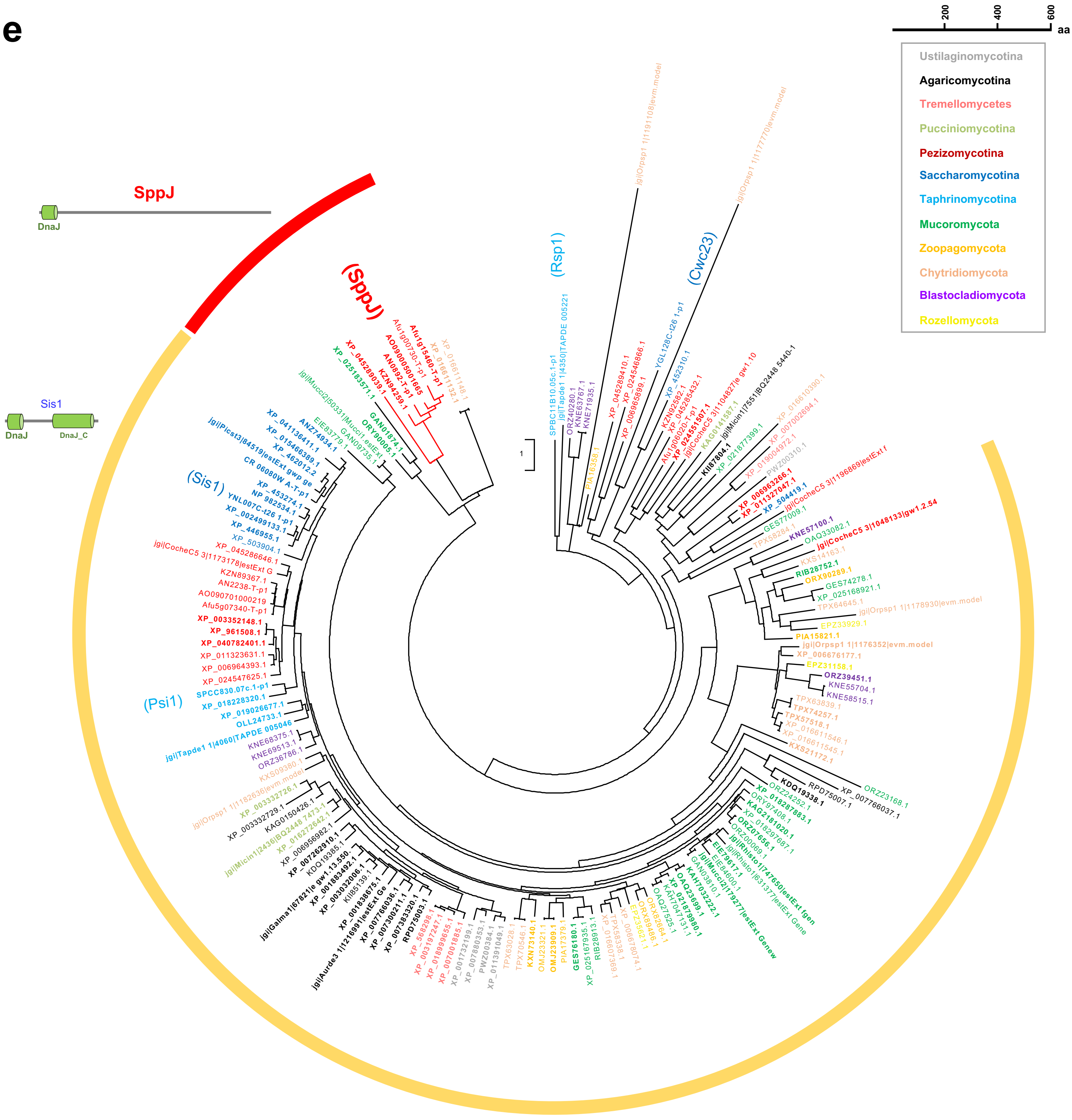
200
400
600
aa
Ustilaginomycotina
Agaricomycotina
Tremellomycetes
Pucciniomycotina
Pezizomycotina
Saccharomycotina
Taphrinomycotina
Mucoromycota
Zoopagomycota
Chytridiomycota
Blastocladiomycota
Rozellomycota
SppJ
DnaJ
Sis1
DnaJ
DnaJ_C
(SppJ)
(Rsp1)
(Cwc23)
(Sis1)
(Psi1)
1

f

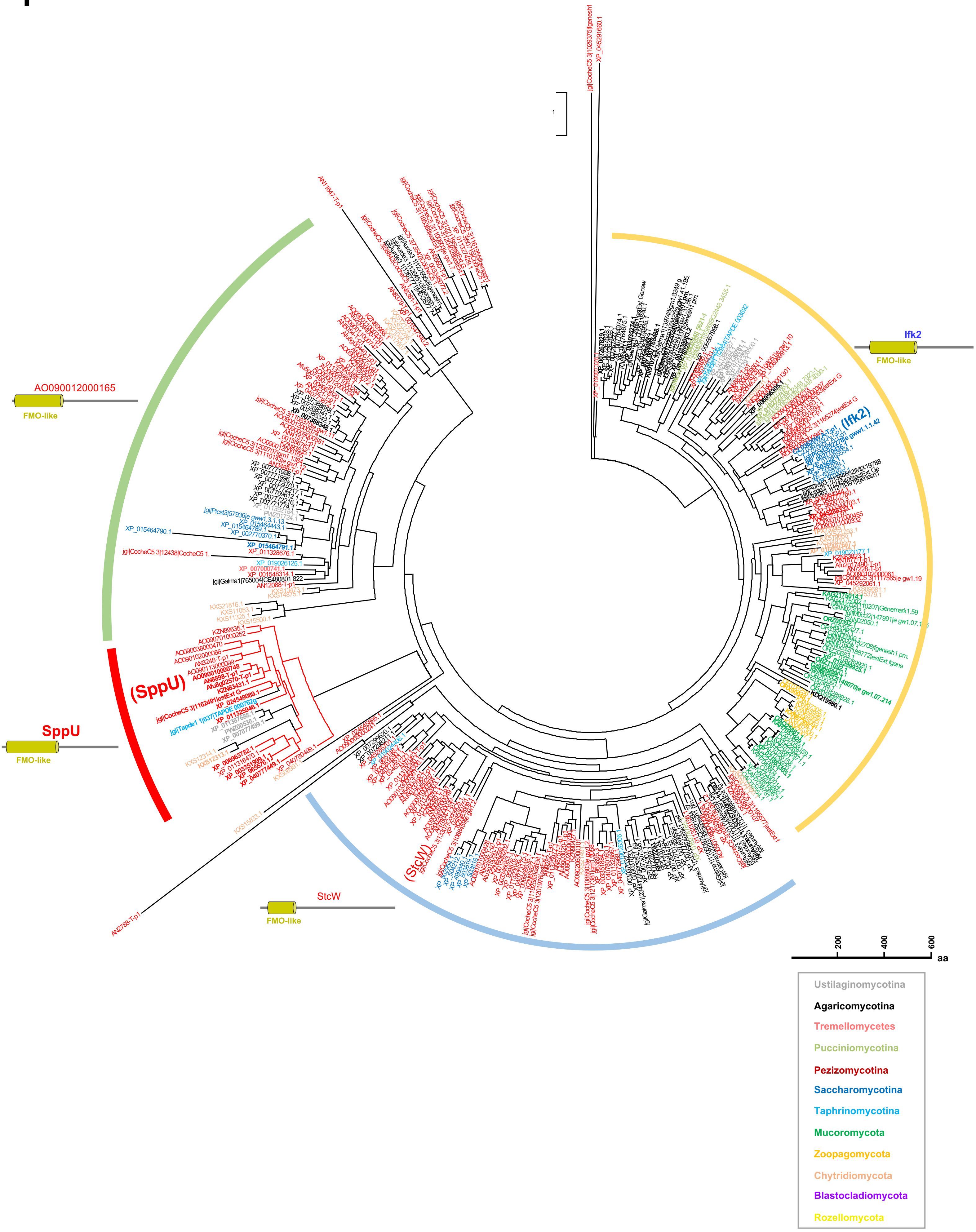

AO090012000165
FMO-like
SppU
FMO-like
(SppU)
StcW
FMO-like
(StcW)
Ifk2
FMO-like
(Ifk2)
1
200
400
600
aa
Ustilaginomycotina
Agaricomycotina
Tremellomycetes
Pucciniomycotina
Pezizomycotina
Saccharomycotina
Taphrinomycotina
Mucoromycota
Zoopagomycota
Chytridiomycota
Blastocladiomycota
Rozellomycota

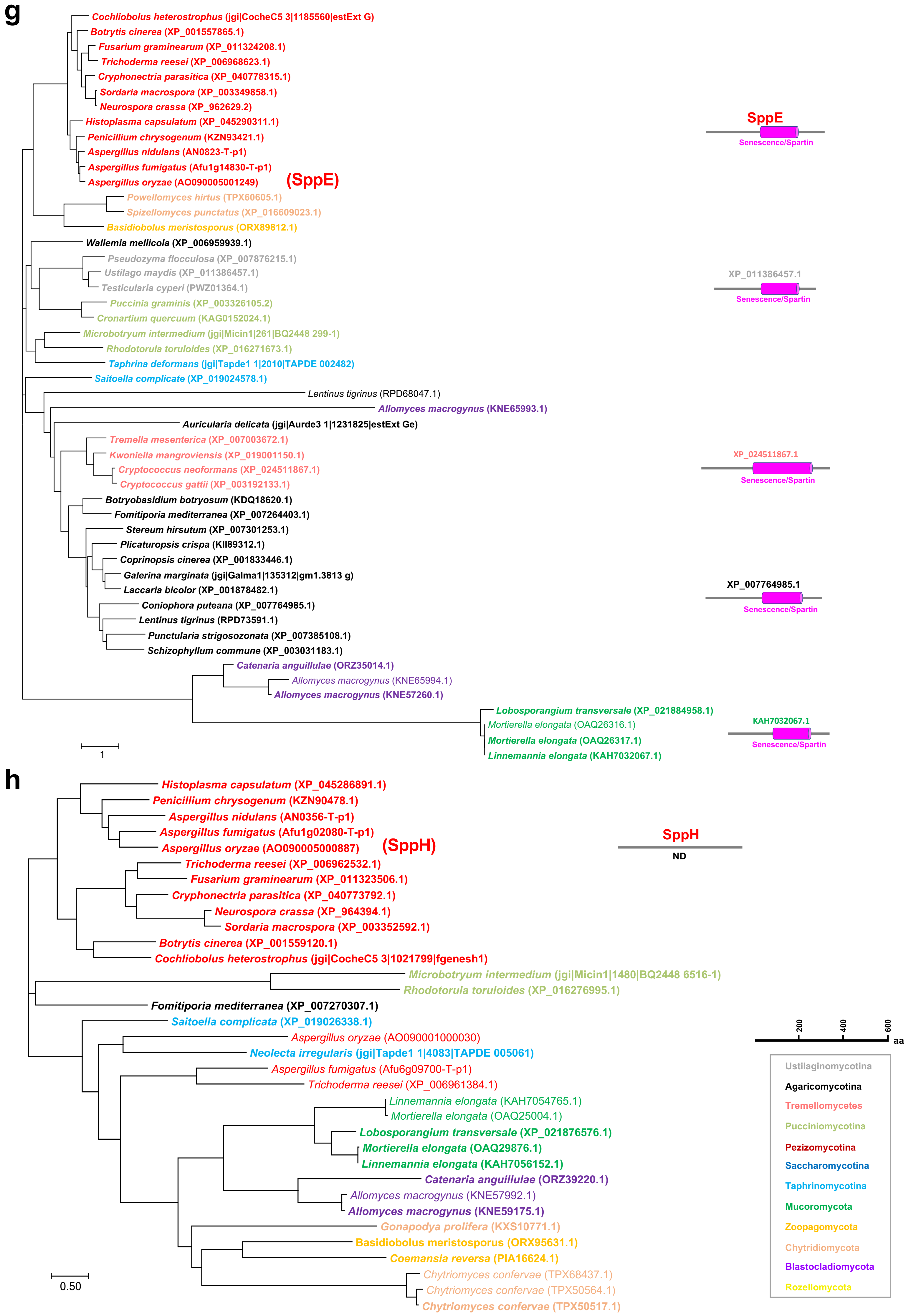

g
Cochliobolus heterostrophus (jgi|CocheC5 3|1185560|estExt G)
Botrytis cinerea (XP_001557865.1)
Fusarium graminearum (XP_011324208.1)
Trichoderma reesei (XP_006968623.1)
Cryphonectria parasitica (XP_040778315.1)
Sordaria macrospora (XP_003349858.1)
Neurospora crassa (XP_962629.2)
Histoplasma capsulatum (XP_045290311.1)
Penicillium chrysogenum (KZN93421.1)
Aspergillus nidulans (AN0823-T-p1)
Aspergillus fumigatus (Afu1g14830-T-p1)
Aspergillus oryzae (AO090005001249)
(SppE)
Powellomyces hirtus (TPX60605.1)
Spizellomyces punctatus (XP_016609023.1)
Basidiobolus meristosporus (ORX89812.1)
Wallemia mellicola (XP_006959939.1)
Pseudozyma flocculosa (XP_007876215.1)
Ustilago maydis (XP_011386457.1)
Testicularia cyperi (PWZ01364.1)
Puccinia graminis (XP_003326105.2)
Cronartium quercuum (KAG0152024.1)
Microbotryum intermedium (jgi|Micin1|261|BQ2448 299-1)
Rhodotorula toruloides (XP_016271673.1)
Taphrina deformans (jgi|Tapde1 1|2010|TAPDE 002482)
Saitoella complicate (XP_019024578.1)
Lentinus tigrinus (RPD68047.1)
Allomyces macrogynus (KNE65993.1)
Auricularia delicata (jgi|Aurde3 1|1231825|estExt Ge)
Tremella mesenterica (XP_007003672.1)
Kwoniella mangroviensis (XP_019001150.1)
Cryptococcus neoformans (XP_024511867.1)
Cryptococcus gattii (XP_003192133.1)
Botryobasidium botryosum (KDQ18620.1)
Fomitiporia mediterranea (XP_007264403.1)
Stereum hirsutum (XP_007301253.1)
Plicaturopsis crispa (KII89312.1)
Coprinopsis cinerea (XP_001833446.1)
Galerina marginata (jgi|Galma1|135312|gm1.3813 g)
Laccaria bicolor (XP_001878482.1)
Coniophora puteana (XP_007764985.1)
Lentinus tigrinus (RPD73591.1)
Punctularia strigosozonata (XP_007385108.1)
Schizophyllum commune (XP_003031183.1)
Catenaria anguillulae (ORZ35014.1)
Allomyces macrogynus (KNE65994.1)
Allomyces macrogynus (KNE57260.1)
Lobosporangium transversale (XP_021884958.1)
Mortierella elongata (OAQ26316.1)
Mortierella elongata (OAQ26317.1)
Linnemannia elongata (KAH7032067.1)
1
SppE
Senescence/Spartin
XP_011386457.1
Senescence/Spartin
XP_024511867.1
Senescence/Spartin
XP_007764985.1
Senescence/Spartin
KAH7032067.1
Senescence/Spartin
h
Histoplasma capsulatum (XP_045286891.1)
Penicillium chrysogenum (KZN90478.1)
Aspergillus nidulans (AN0356-T-p1)
Aspergillus fumigatus (Afu1g02080-T-p1)
Aspergillus oryzae (AO090005000887)
(SppH)
Trichoderma reesei (XP_006962532.1)
Fusarium graminearum (XP_011323506.1)
Cryphonectria parasitica (XP_040773792.1)
Neurospora crassa (XP_964394.1)
Sordaria macrospora (XP_003352592.1)
Botrytis cinerea (XP_001559120.1)
Cochliobolus heterostrophus (jgi|CocheC5 3|1021799|fgenesh1)
Microbotryum intermedium (jgi|Micin1|1480|BQ2448 6516-1)
Rhodotorula toruloides (XP_016276995.1)
Fomitiporia mediterranea (XP_007270307.1)
Saitoella complicata (XP_019026338.1)
Aspergillus oryzae (AO090001000030)
Neolecta irregularis (jgi|Tapde1 1|4083|TAPDE 005061)
Aspergillus fumigatus (Afu6g09700-T-p1)
Trichoderma reesei (XP_006961384.1)
Linnemannia elongata (KAH7054765.1)
Mortierella elongata (OAQ25004.1)
Lobosporangium transversale (XP_021876576.1)
Mortierella elongata (OAQ29876.1)
Linnemannia elongata (KAH7056152.1)
Catenaria anguillulae (ORZ39220.1)
Allomyces macrogynus (KNE57992.1)
Allomyces macrogynus (KNE59175.1)
Gonapodya prolifera (KXS10771.1)
Basidiobolus meristosporus (ORX95631.1)
Coemansia reversa (PIA16624.1)
Chytriomyces confervae (TPX68437.1)
Chytriomyces confervae (TPX50564.1)
Chytriomyces confervae (TPX50517.1)
0.50
SppH
ND
200
400
600
aa
Ustilaginomycotina
Agaricomycotina
Tremellomycetes
Pucciniomycotina
Pezizomycotina
Saccharomycotina
Taphrinomycotina
Mucoromycota
Zoopagomycota
Chytridiomycota
Blastocladiomycota
Rozellomycota

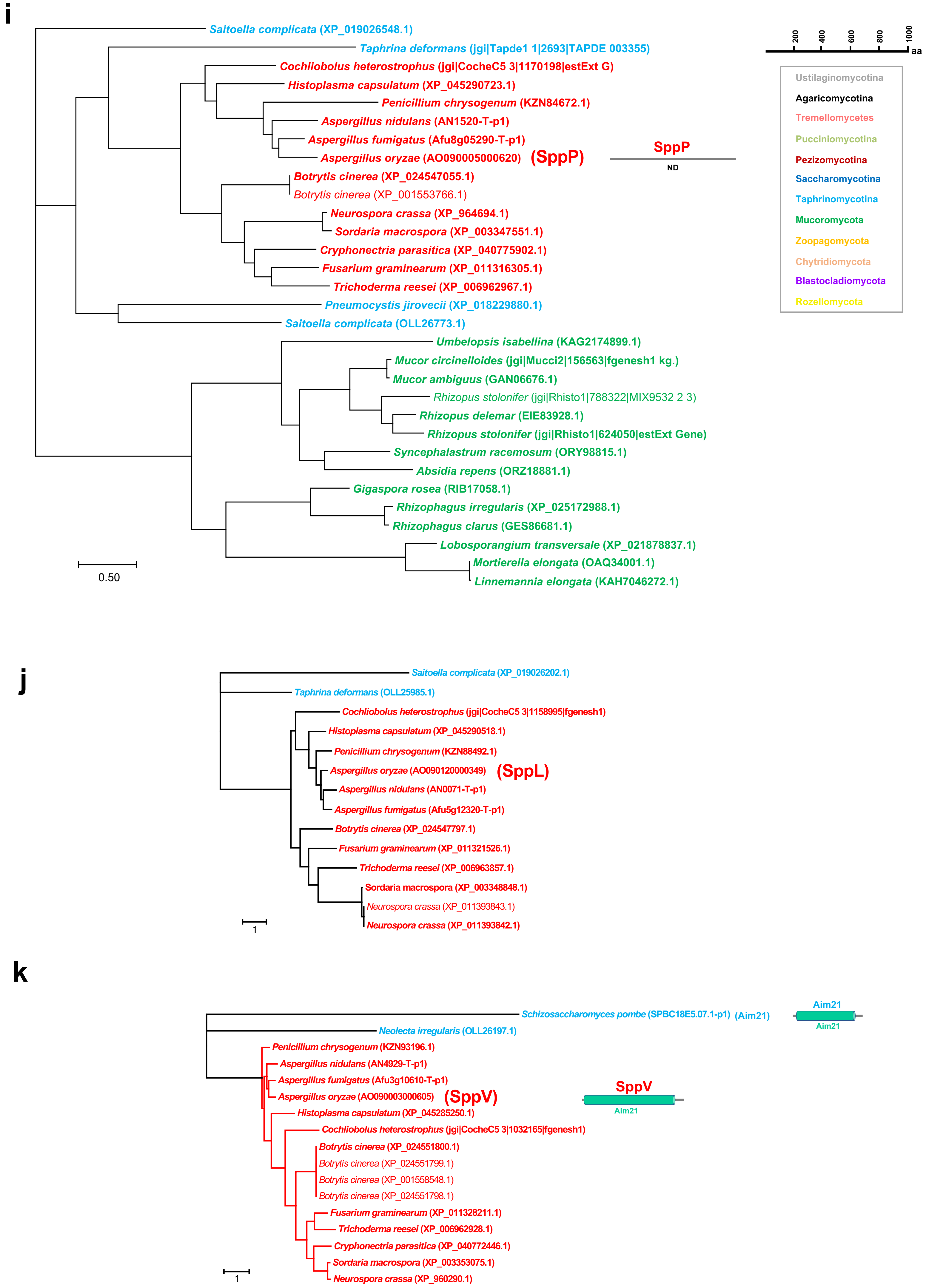
i
Saitoella complicata (XP_019026548.1)
Taphrina deformans (jgi|Tapde1 1|2693|TAPDE 003355)
Cochliobolus heterostrophus (jgi|CocheC5 3|1170198|estExt G)
Histoplasma capsulatum (XP_045290723.1)
Penicillium chrysogenum (KZN84672.1)
Aspergillus nidulans (AN1520-T-p1)
Aspergillus fumigatus (Afu8g05290-T-p1)
Aspergillus oryzae (AO090005000620) (SppP)
Botrytis cinerea (XP_024547055.1)
Botrytis cinerea (XP_001553766.1)
Neurospora crassa (XP_964694.1)
Sordaria macrospora (XP_003347551.1)
Cryphonectria parasitica (XP_040775902.1)
Fusarium graminearum (XP_011316305.1)
Trichoderma reesei (XP_006962967.1)
Pneumocystis jirovecii (XP_018229880.1)
Saitoella complicata (OLL26773.1)
Umbelopsis isabellina (KAG2174899.1)
Mucor circinelloides (jgi|Mucci2|156563|fgenesh1 kg.)
Mucor ambiguus (GAN06676.1)
Rhizopus stolonifer (jgi|Rhisto1|788322|MIX9532 2 3)
Rhizopus delemar (EIE83928.1)
Rhizopus stolonifer (jgi|Rhisto1|624050|estExt Gene)
Syncephalastrum racemosum (ORY98815.1)
Absidia repens (ORZ18881.1)
Gigaspora rosea (RIB17058.1)
Rhizophagus irregularis (XP_025172988.1)
Rhizophagus clarus (GES86681.1)
Lobosporangium transversale (XP_021878837.1)
Mortierella elongata (OAQ34001.1)
Linnemannia elongata (KAH7046272.1)
0.50
200
400
600
800
1000
aa
SppP
ND
Ustilaginomycotina
Agaricomycotina
Tremellomycetes
Pucciniomycotina
Pezizomycotina
Saccharomycotina
Taphrinomycotina
Mucoromycota
Zoopagomycota
Chytridiomycota
Blastocladiomycota
Rozellomycota
j
Saitoella complicata (XP_019026202.1)
Taphrina deformans (OLL25985.1)
Cochliobolus heterostrophus (jgi|CocheC5 3|1158995|fgenesh1)
Histoplasma capsulatum (XP_045290518.1)
Penicillium chrysogenum (KZN88492.1)
Aspergillus oryzae (AO090120000349) (SppL)
Aspergillus nidulans (AN0071-T-p1)
Aspergillus fumigatus (Afu5g12320-T-p1)
Botrytis cinerea (XP_024547797.1)
Fusarium graminearum (XP_011321526.1)
Trichoderma reesei (XP_006963857.1)
Sordaria macrospora (XP_003348848.1)
Neurospora crassa (XP_011393843.1)
Neurospora crassa (XP_011393842.1)
1
k
Schizosaccharomyces pombe (SPBC18E5.07.1-p1) (Aim21)
Neolecta irregularis (OLL26197.1)
Penicillium chrysogenum (KZN93196.1)
Aspergillus nidulans (AN4929-T-p1)
Aspergillus fumigatus (Afu3g10610-T-p1)
Aspergillus oryzae (AO090003000605) (SppV)
Histoplasma capsulatum (XP_045285250.1)
Cochliobolus heterostrophus (jgi|CocheC5 3|1032165|fgenesh1)
Botrytis cinerea (XP_024551800.1)
Botrytis cinerea (XP_024551799.1)
Botrytis cinerea (XP_001558548.1)
Botrytis cinerea (XP_024551798.1)
Fusarium graminearum (XP_011328211.1)
Trichoderma reesei (XP_006962928.1)
Cryphonectria parasitica (XP_040772446.1)
Sordaria macrospora (XP_003353075.1)
Neurospora crassa (XP_960290.1)
1
Aim21
Aim21
SppV
Aim21

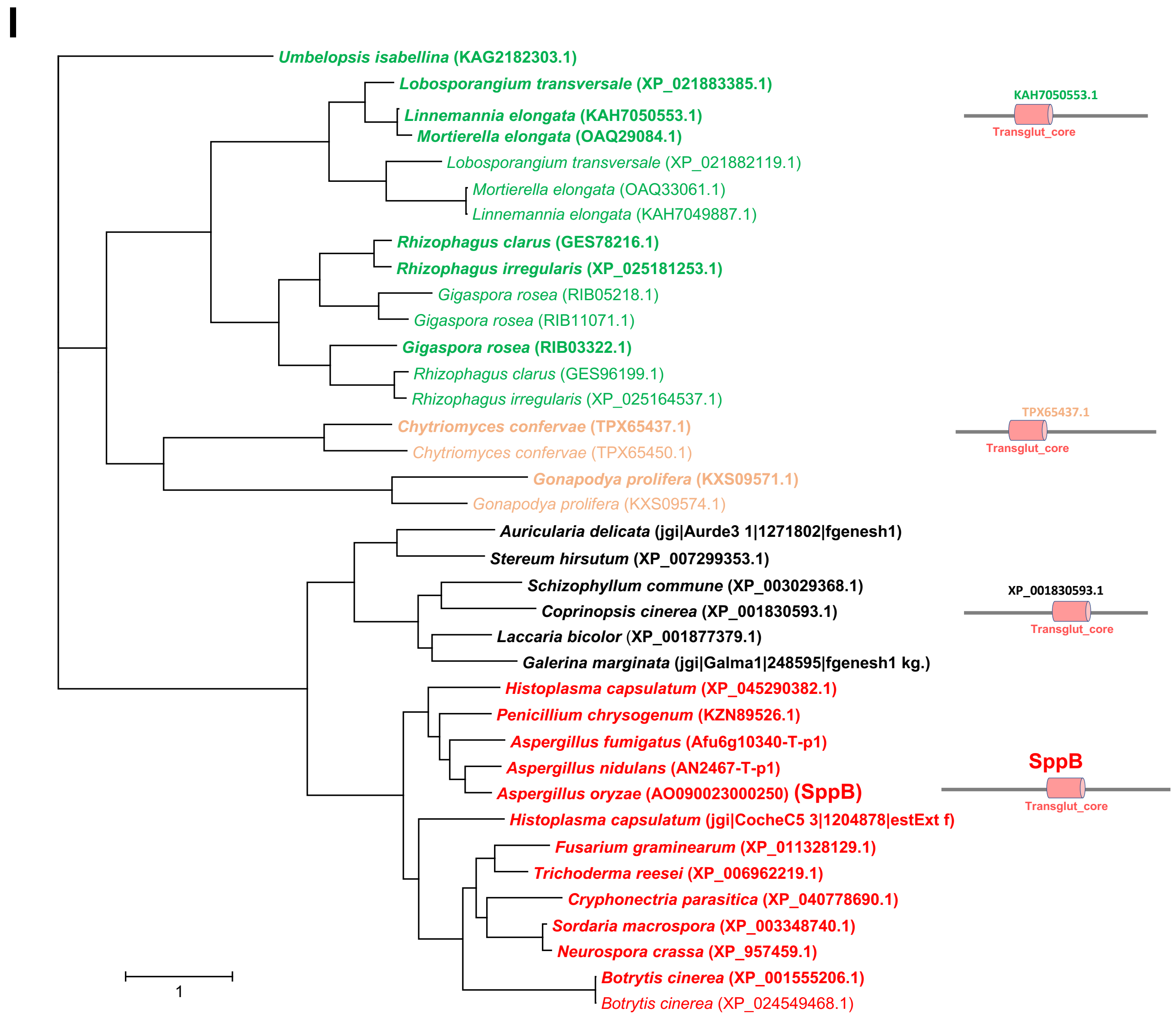


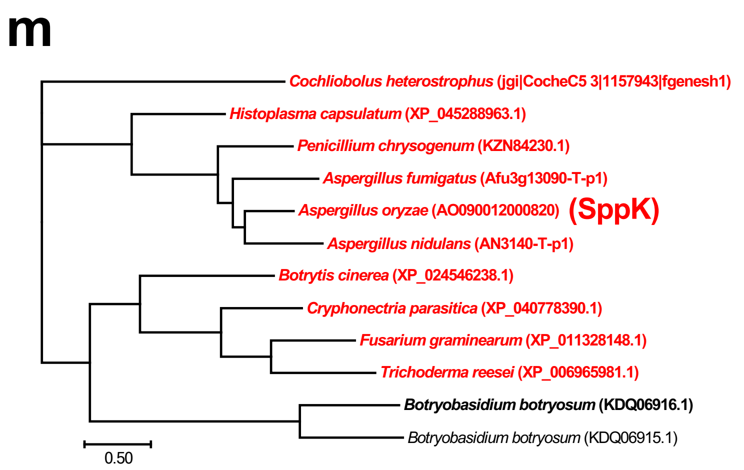


**Supplementary Figure 8: Phylogenies of 13 SPP proteins having orthologs outside of Pezizomycotina.** Maximum-likelihood trees were generated using MEGA 7 for proteins belonging to the same orthologous groups classified using OrthoFinder. Domains were predicted using the Simple modular architecture research tool (SMART). Diagrams of protein domain structures are shown with the respective clades. Bold letters represent proteins used for the substitution rate analysis in Fig. 7. **a-m** Phylogenies of proteins in the orthologous groups including SPP proteins.

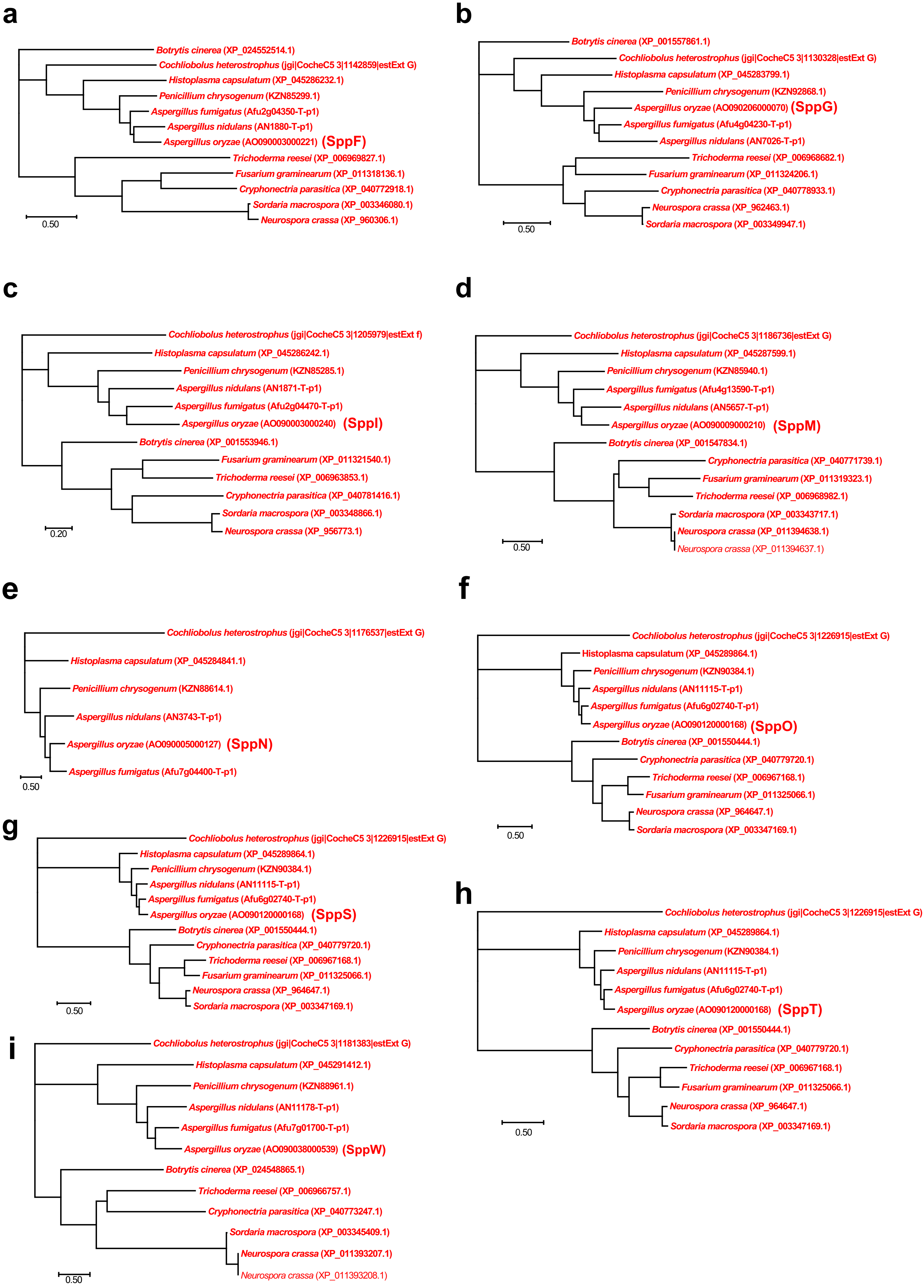

a
Botrytis cinerea (XP_024552514.1)
Cochliobolus heterostrophus (jgi|CocheC5 3|1142859|estExt G)
Histoplasma capsulatum (XP_045286232.1)
Penicillium chrysogenum (KZN85299.1)
Aspergillus fumigatus (Afu2g04350-T-p1)
Aspergillus nidulans (AN1880-T-p1)
Aspergillus oryzae (AO090003000221) (SppF)
Trichoderma reesei (XP_006969827.1)
Fusarium graminearum (XP_011318136.1)
Cryphonectria parasitica (XP_040772918.1)
Sordaria macrospora (XP_003346080.1)
Neurospora crassa (XP_960306.1)
0.50
b
Botrytis cinerea (XP_001557861.1)
Cochliobolus heterostrophus (jgi|CocheC5 3|1130328|estExt G)
Histoplasma capsulatum (XP_045283799.1)
Penicillium chrysogenum (KZN92868.1)
Aspergillus oryzae (AO090206000070) (SppG)
Aspergillus fumigatus (Afu4g04230-T-p1)
Aspergillus nidulans (AN7026-T-p1)
Trichoderma reesei (XP_006968682.1)
Fusarium graminearum (XP_011324206.1)
Cryphonectria parasitica (XP_040778933.1)
Neurospora crassa (XP_962463.1)
Sordaria macrospora (XP_003349947.1)
0.50
c
Cochliobolus heterostrophus (jgi|CocheC5 3|1205979|estExt f)
Histoplasma capsulatum (XP_045286242.1)
Penicillium chrysogenum (KZN85285.1)
Aspergillus nidulans (AN1871-T-p1)
Aspergillus fumigatus (Afu2g04470-T-p1)
Aspergillus oryzae (AO090003000240) (SppI)
Botrytis cinerea (XP_001553946.1)
Fusarium graminearum (XP_011321540.1)
Trichoderma reesei (XP_006963853.1)
Cryphonectria parasitica (XP_040781416.1)
Sordaria macrospora (XP_003348866.1)
Neurospora crassa (XP_956773.1)
0.20
d
Cochliobolus heterostrophus (jgi|CocheC5 3|1186736|estExt G)
Histoplasma capsulatum (XP_045287599.1)
Penicillium chrysogenum (KZN85940.1)
Aspergillus fumigatus (Afu4g13590-T-p1)
Aspergillus nidulans (AN5657-T-p1)
Aspergillus oryzae (AO090009000210) (SppM)
Botrytis cinerea (XP_001547834.1)
Cryphonectria parasitica (XP_040771739.1)
Fusarium graminearum (XP_011319323.1)
Trichoderma reesei (XP_006968982.1)
Sordaria macrospora (XP_003343717.1)
Neurospora crassa (XP_011394638.1)
Neurospora crassa (XP_011394637.1)
0.50
e
Cochliobolus heterostrophus (jgi|CocheC5 3|1176537|estExt G)
Histoplasma capsulatum (XP_045284841.1)
Penicillium chrysogenum (KZN88614.1)
Aspergillus nidulans (AN3743-T-p1)
Aspergillus oryzae (AO090005000127) (SppN)
Aspergillus fumigatus (Afu7g04400-T-p1)
0.50
f
Cochliobolus heterostrophus (jgi|CocheC5 3|1226915|estExt G)
Histoplasma capsulatum (XP_045289864.1)
Penicillium chrysogenum (KZN90384.1)
Aspergillus nidulans (AN11115-T-p1)
Aspergillus fumigatus (Afu6g02740-T-p1)
Aspergillus oryzae (AO090120000168) (SppO)
Botrytis cinerea (XP_001550444.1)
Cryphonectria parasitica (XP_040779720.1)
Trichoderma reesei (XP_006967168.1)
Fusarium graminearum (XP_011325066.1)
Neurospora crassa (XP_964647.1)
Sordaria macrospora (XP_003347169.1)
0.50
g
Cochliobolus heterostrophus (jgi|CocheC5 3|1226915|estExt G)
Histoplasma capsulatum (XP_045289864.1)
Penicillium chrysogenum (KZN90384.1)
Aspergillus nidulans (AN11115-T-p1)
Aspergillus fumigatus (Afu6g02740-T-p1)
Aspergillus oryzae (AO090120000168) (SppS)
Botrytis cinerea (XP_001550444.1)
Cryphonectria parasitica (XP_040779720.1)
Trichoderma reesei (XP_006967168.1)
Fusarium graminearum (XP_011325066.1)
Neurospora crassa (XP_964647.1)
Sordaria macrospora (XP_003347169.1)
0.50
h
Cochliobolus heterostrophus (jgi|CocheC5 3|1226915|estExt G)
Histoplasma capsulatum (XP_045289864.1)
Penicillium chrysogenum (KZN90384.1)
Aspergillus nidulans (AN11115-T-p1)
Aspergillus fumigatus (Afu6g02740-T-p1)
Aspergillus oryzae (AO090120000168) (SppT)
Botrytis cinerea (XP_001550444.1)
Cryphonectria parasitica (XP_040779720.1)
Trichoderma reesei (XP_006967168.1)
Fusarium graminearum (XP_011325066.1)
Neurospora crassa (XP_964647.1)
Sordaria macrospora (XP_003347169.1)
0.50
i
Cochliobolus heterostrophus (jgi|CocheC5 3|1181383|estExt G)
Histoplasma capsulatum (XP_045291412.1)
Penicillium chrysogenum (KZN88961.1)
Aspergillus nidulans (AN11178-T-p1)
Aspergillus fumigatus (Afu7g01700-T-p1)
Aspergillus oryzae (AO090038000539) (SppW)
Botrytis cinerea (XP_024548865.1)
Trichoderma reesei (XP_006966757.1)
Cryphonectria parasitica (XP_040773247.1)
Sordaria macrospora (XP_003345409.1)
Neurospora crassa (XP_011393207.1)
Neurospora crassa (XP_011393208.1)
0.50

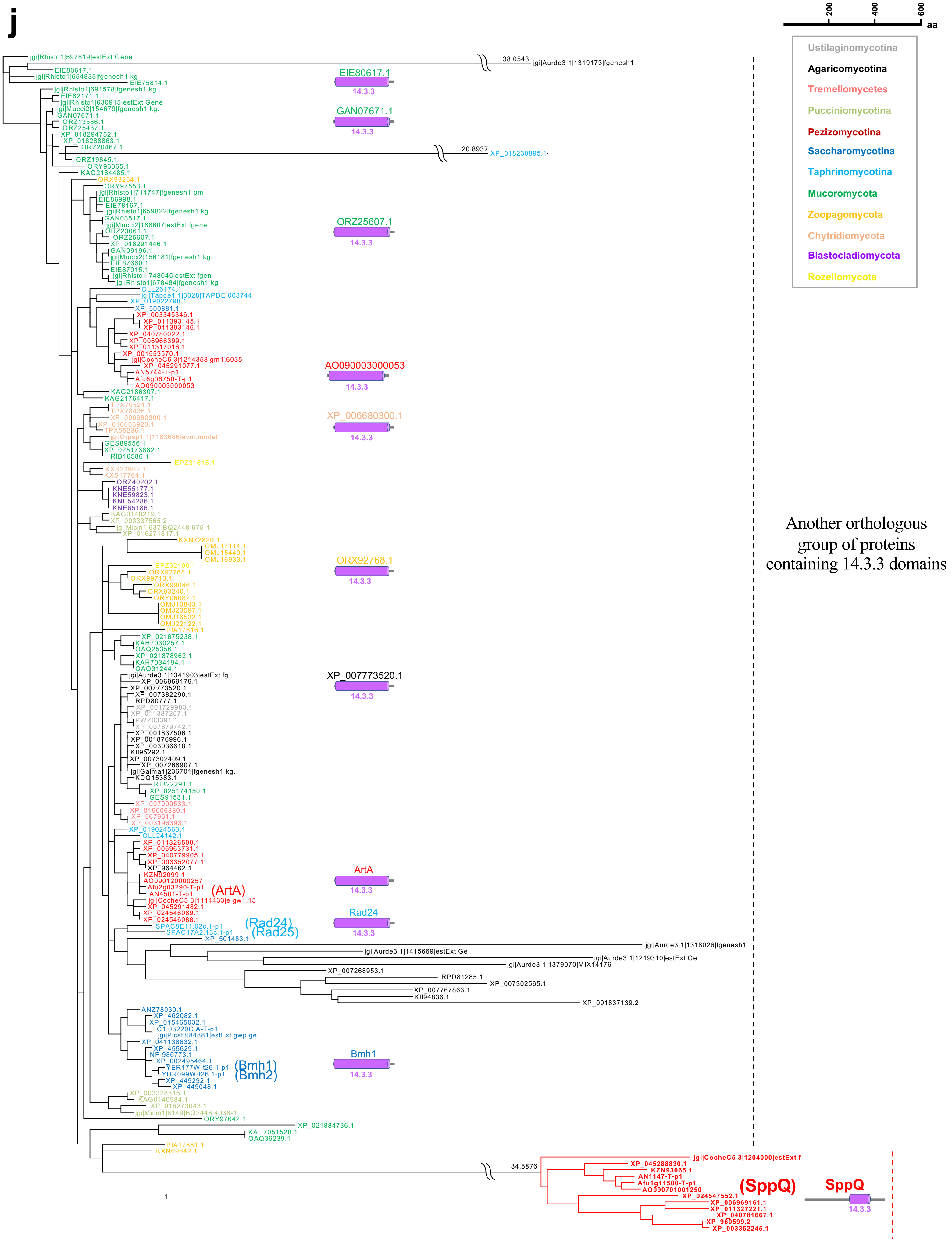


**Supplementary Figure 9: Phylogenies of Pezizomycotina-specific SPP proteins.** Maximum-likelihood trees were generated using MEGA 7 for proteins belonging to the orthologous groups classified using OrthoFinder. Domains were predicted using the Simple modular architecture research tool (SMART). Diagrams of protein domain structures are shown with the respective clades. Bold letters represent the proteins used for substitution rate analysis in Fig. 7. **a-i** Phylogenies of proteins in the same orthologous groups including SPP proteins. **j** Phylogeny of proteins in the orthologous group including SppQ. Note that the phylogeny also includes another orthologous group proteins containing 14.3.3 domains.

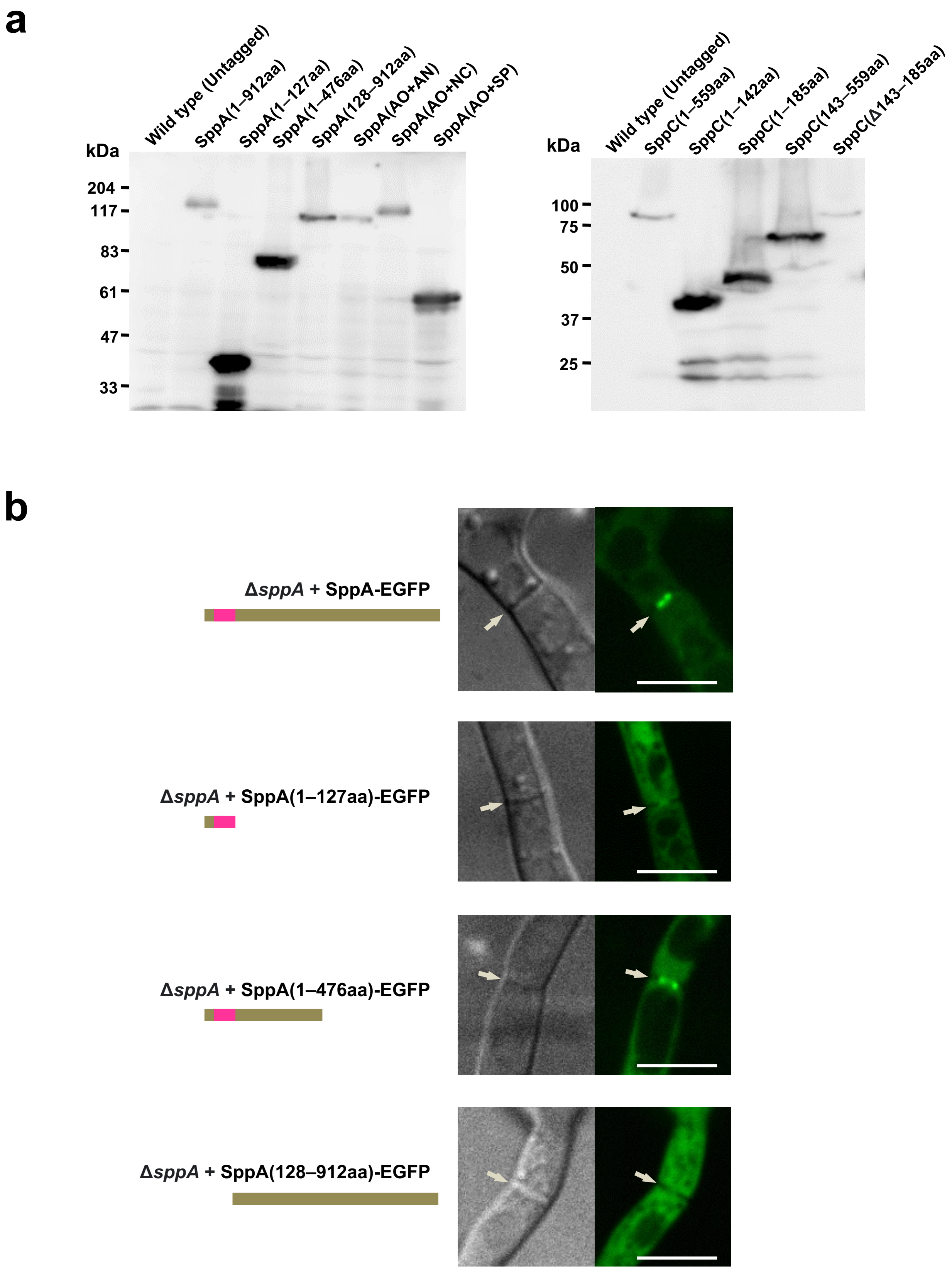


**Supplementary Figure 10: Expression of SppA and SppC truncations. a** Expression of SppA/SppC variants, including truncations and chimeric proteins, confirmed by western blotting. Crude proteins (20 µg) isolated from the individual strain grown in DPY liquid medium were loaded in each lane. Expression of SppA/C and truncated/chimeric variants fused with EGFP was confirmed by western blotting using anti-GFP antibody. Note that low-molecular weight variants showed high signal intensities owing to high transfer efficiency. Source data are provided as a Source Data file. **b** The N-terminus of SppA is essential for its localization around the septal pore. Single confocal fluorescence images are shown, and arrows indicate apical septa. Scale bars, 5 µm.

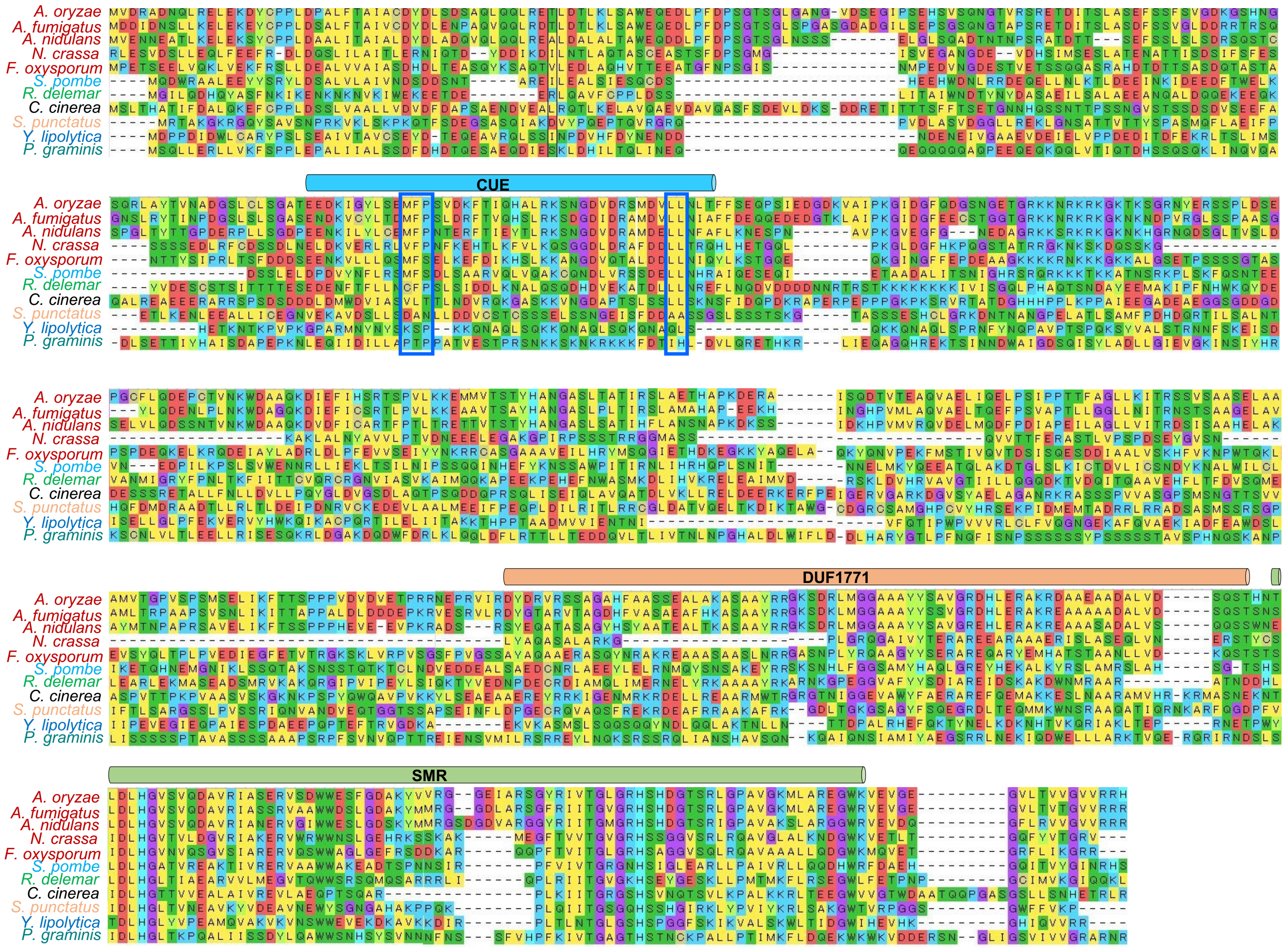

**Supplementary Figure 11: Multiple sequence alignment of SppC and orthologs**. Blue boxes represent two conserved motifs (MFP and LL), which are essential for ubiquitin-related function.
