# Supplementary material for: Large-scale identification of genes involved in septal pore plugging in multicellular fungi
Source: Nat Commun. 2023 Mar 17;14:1418. doi: 10.1038/s41467-023-36925-y (PMC10023807; doi:10.1038/s41467-023-36925-y)
Supplement: Supplementary file 2 — Description of Additional Supplementary Files [file 41467_2023_36925_MOESM2_ESM.pdf]

Description of Additional Supplementary Files:

Supplementary Data 1a: BLASTp-based comparison of genes with known function in cellular morphogenesis between septal porebearing and -lacking ascomycete species
Supplementary Data 1b: BLASTp-based comparison genes involved in septal pore regulation between septal pore-bearing and - lacking ascomycete species

Supplementary Data 2: 776 candidate septal pore proteins

Supplementary Data 3: Strains used for the current study

Supplementary Data 4: List of genes not producing apparent EGFP signal

Supplementary Data 5a: Substitution rates of SPP orthologous group proteins in fungi
Supplementary Data 5b: SPP orthologous group proteins used in substitution rate analysis
Supplementary Data 5c: Fungal proteins in the orthologous group containing each SPP protein
Supplementary Data 5d: List of 81 fungal proteomes used in orthologous group analysis

Supplementary Data 6: Primers used for the current study
