# Supplementary material for: Large-scale identification of genes involved in septal pore plugging in multicellular fungi
Source: Nat Commun. 2023 Mar 17;14:1418. doi: 10.1038/s41467-023-36925-y (PMC10023807; doi:10.1038/s41467-023-36925-y)
Supplement: Supplementary file 9 — Reporting Summary [file 41467_2023_36925_MOESM9_ESM.pdf]

nature portfolio

Corresponding author(s): Jun-ichi Maruyama

Last updated by author(s): Feb 16, 2023

Reporting Summary

Nature Portfolio wishes to improve the reproducibility of the work that we publish. This form provides structure for consistency and transparency in reporting. For further information on Nature Portfolio policies, see our Editorial Policies and the Editorial Policy Checklist.

Statistics

For all statistical analyses, confirm that the following items are present in the figure legend, table legend, main text, or Methods section.

n/a	Confirmed	
☐	☒	The exact sample size (*n*) for each experimental group/condition, given as a discrete number and unit of measurement
☐	☒	A statement on whether measurements were taken from distinct samples or whether the same sample was measured repeatedly
☐	☒	The statistical test(s) used AND whether they are one- or two-sided<br>*Only common tests should be described solely by name; describe more complex techniques in the Methods section.*
☒	☐	A description of all covariates tested
☐	☒	A description of any assumptions or corrections, such as tests of normality and adjustment for multiple comparisons
☐	☒	A full description of the statistical parameters including central tendency (e.g. means) or other basic estimates (e.g. regression coefficient) AND variation (e.g. standard deviation) or associated estimates of uncertainty (e.g. confidence intervals)
☐	☒	For null hypothesis testing, the test statistic (e.g. *F*, *t*, *r*) with confidence intervals, effect sizes, degrees of freedom and *P* value noted<br>*Give P values as exact values whenever suitable.*
☒	☐	For Bayesian analysis, information on the choice of priors and Markov chain Monte Carlo settings
☒	☐	For hierarchical and complex designs, identification of the appropriate level for tests and full reporting of outcomes
☒	☐	Estimates of effect sizes (e.g. Cohen's *d*, Pearson's *r*), indicating how they were calculated

Our web collection on statistics for biologists contains articles on many of the points above.

Software and code

Policy information about availability of computer code

Data collection	1. Confocal images were acquired using Andor iQ 1.9 (Andor Technology PLC) software.<br>2. Gels and blots were imaged using LAS-4000 image analyzer (GE Healthcare) software.
Data analysis	1. Microscopic images were analyzed using Andor iQ 1.9 software.<br>2. Images were cropped in Microsoft PowerPoint (version 16.70).<br>3. Figures were generated using Microsoft PowerPoint (version 16.70).<br>4. Statistical analysis was performed with Microsoft Excel (version 16.70).<br>5. Protein sequences were aligned with ClustalW 2.1 and output using BoxShade server version 3.21.<br>6. Orthologous group analysis was performed with orthofinder 2.3.14.<br>7. Maximum likelihood phylogenies were inferred using RAxML in ClustalW 2.1.<br>8. Phylogenetic trees were constructed with Molecular Evolutionary Genetics Tool Mega version 7.

For manuscripts utilizing custom algorithms or software that are central to the research but not yet described in published literature, software must be made available to editors and reviewers. We strongly encourage code deposition in a community repository (e.g. GitHub). See the Nature Portfolio guidelines for submitting code & software for further information.

Data

Policy information about availability of data

All manuscripts must include a data availability statement. This statement should provide the following information, where applicable:

- Accession codes, unique identifiers, or web links for publicly available datasets
- A description of any restrictions on data availability
- For clinical datasets or third party data, please ensure that the statement adheres to our policy

All data supporting the findings of the present study are available within this paper, Supplementary Information and Source Data.
Gene sequences of Aspergillus oryzae can be found in FungiDB (https://fungidb.org/fungidb/app), and the sequences of fungal orthologs can be found in NCBI (https://www.ncbi.nlm.nih.gov/).
All biological materials generated in this study are available from the corresponding author upon reasonable request.

Human research participants

Policy information about studies involving human research participants and Sex and Gender in Research.

Reporting on sex and gender	Not Applicable
Population characteristics	Not Applicable
Recruitment	Not Applicable
Ethics oversight	Not Applicable

Note that full information on the approval of the study protocol must also be provided in the manuscript.

Field-specific reporting

Please select the one below that is the best fit for your research. If you are not sure, read the appropriate sections before making your selection.

[x] Life sciences [ ] Behavioural & social sciences [ ] Ecological, evolutionary & environmental sciences

For a reference copy of the document with all sections, see nature.com/documents/nr-reporting-summary-flat.pdf

Life sciences study design

All studies must disclose on these points even when the disclosure is negative.

Sample size	No sample size calculation were performed. For quantitative analyses, at least 3 measurements were performed for each strain. For microscopic analyses, at least 4 independent images were taken for each strain. These choices of sample sizes are generally accepted in the corresponding field.
Data exclusions	No data were excluded.
Replication	Experiments were replicated at least 3 times.
Randomization	None of the experimental designs required randomization. As the strains used in this study were genetically designed, the specified samples were not randomly allocated into experimental groups.
Blinding	None of the experimental designs required blinding in this study, which does not include any clinical assessments.

Reporting for specific materials, systems and methods

We require information from authors about some types of materials, experimental systems and methods used in many studies. Here, indicate whether each material, system or method listed is relevant to your study. If you are not sure if a list item applies to your research, read the appropriate section before selecting a response.

Materials & experimental systems

n/a	Involved in the study	
☐	☒	Antibodies
☒	☐	Eukaryotic cell lines
☒	☐	Palaeontology and archaeology
☒	☐	Animals and other organisms
☒	☐	Clinical data
☒	☐	Dual use research of concern

Methods

n/a	Involved in the study	
☒	☐	ChIP-seq
☒	☐	Flow cytometry
☒	☐	MRI-based neuroimaging

Antibodies

Antibodies used

Living Colors A.V. (anti-GFP) monoclonal antibody (1:2,000 dilution, cat # 632380, Clontech)
Peroxidase-labeled anti-mouse IgG (H + L) (1:2,000 dilution, cat # PI-2000, Vector Laboratories)

Validation

Validations of the antibodies used in this study are available on the following manufacturers' websites.

Living Colors A.V. (anti-GFP) monoclonal antibody (https://www.takarabio.com/documents/Certificate%20of%20Analysis/632380/632380-632381-070313.pdf)
Peroxidase-labeled anti-mouse IgG (https://vectorlabs.com/products/antibodies/peroxidase-horse-anti-mouse-igg)
